# Dynamic reverse Cl^−^ driven integration of sludge conditioning and dewatering

**DOI:** 10.1038/s41467-025-57878-4

**Published:** 2025-03-19

**Authors:** Xiujia You, Hanmin Zhang, Hongjun Lin, Linhua Rao

**Affiliations:** 1 Key Laboratory of Industrial Ecology and Environmental Engineering (Ministry of Education, MOE), School of Environmental Science and Technology, Dalian University of Technology, Dalian, 116024 China; 2 Key Laboratory of Watershed Earth Surface Processes and Ecological Security, College of Geography and Environmental Sciences, Zhejiang Normal University, Jinhua, 321004 China

**Keywords:** Pollution remediation, Environmental impact, Energy and society, Engineering

## Abstract

Gel fouling is a major rate-limiting factor for forward osmosis (FO) dewatering of waste activated sludge (WAS). This study proposes a novel FO system, assisted by in-situ ultraviolet/electrooxidation (UV/E-Cl) driven by dynamic reverse chloride ions (Cl^−^), for simultaneous WAS conditioning and dewatering. Superior filtration performances were achieved, with water flux reaching 614% of the control and filtration resistance reduced by orders of magnitude, primarily due to the targeted attack on protein and polysaccharide fractions within extracellular polymeric substances (EPS). Density functional theory (DFT) simulations identified that protein-polysaccharide interactions prefer a specific linear configuration, driving cross-linked network formation. Interfacial thermodynamics demonstrated that UV/E-Cl decreased foulant adhesion energy on the membrane surface by 97.51% through cleaving cross-links. Crucially, this work provides the quantitative thermodynamic evidence that shifts in water occurrence states surrounding network pores from bound to free water dominate gel fouling mitigation, with chemical potential variation accounting for 90.71% of filtration resistance.

## Abstract (web summary)

Gel fouling is a major rate-limiting factor for forward osmosis (FO) dewatering of waste activated sludge (WAS). Here, authors propose a novel FO system, assisted by in situ ultraviolet /electrooxidation (UV/E-Cl) driven by dynamic reverse chloride ion (Cl − ), for simultaneous WAS conditioning and dewatering.

## Introduction

In China, waste activated sludge (WAS), an inevitable byproduct from biological wastewater treatment processes, has increased annually to approximately 60 million tons with over 95% water content^1,2^. This raises deep concerns about the required land footprint and associated environmental risks. Dewatering is generally the first step in low-carbon disposal and subsequent WAS management^1,3^. Forward osmosis (FO), with relatively lower energy consumption, better effluent quality, and superior water flux stability compared to traditional dewatering approaches, has shown considerable promise in dewatering applications^4,5^. Nevertheless, FO dewatering efficiency is inevitably limited by membrane fouling, especially gel fouling caused by extracellular polymeric substances (EPS) with highly hydrated colloidal properties in WAS^5–7^. It has been suggested that proteins and polysaccharides in EPS are key contributors to gel layer formation, attributed to their strong ability to mechanically trap interstitial bound water through specific cross-linked network structures and adsorb interfacial bound water through abundant hydrophilic sites (based on dipole-dipole or ion-dipole interactions)^6,7^. Although strategies such as ultrasound^8,9^, membrane modification^10,11^ and chemical additives^12^ have been explored to mitigate FO membrane fouling during WAS dewatering, there is no doubt that they are process complexity, poor continuity, and higher operational costs. Moreover, these techniques may not be as efficient as desired due to their non-specific targeting of proteins and polysaccharides.

Advanced oxidation processes (AOPs), focused on enhancing EPS depolymerization and eliminating its water-holding capacity, have been widely applied in sludge dewatering preconditioning^13–15^. In contrast to HO• and SO_4_^−^•, which are poorly selective and susceptible to quenching by dissolved organic matter and inorganic salts (e.g., CO_3_^2−^, Cl^−^)^16,17^, chlorine radical (Cl•) is noted for its high reduction potential (∼2.47 V) and ability to selectively attack electron-rich molecules at nearly diffusion-controlled rates (∼10^10 ^M^–1^ s^–1^)^18–20^. It is anticipated that Cl• could selectively react with electron-rich sites, such as glycosidic bonds (C-O-C) in polysaccharides and peptide bonds (CO-NH) in proteins^21,22^. This targeted attack is expected to effectively disrupt the structural integrity of polysaccharides and proteins, diminishing their water-holding capacity and hindering their diffusion to the membrane surface^23^. Despite the potential benefits of chlorine-based AOPs in controlling gel-like membrane fouling associated with polysaccharides and proteins, relevant research is limited, possibly due to concerns about the risks of frequent liquid chlorine transportation and management^24^. Given that reverse salt diffusion is inevitable in FO processes, we propose to harness this unwanted phenomenon and turn it into a driving force for in-situ Cl• generation. Accordingly, it may be feasible to construct a green, flexible, less material-restricted, and more cost-effective integrated module, i.e., reverse Cl^−^ driven in-situ UV/E-Cl (electrochemical oxidation of Cl^−^ combined with UV irradiation) assisted FO process. This scenario should achieve a “mutually-beneficial” goal in terms of simultaneous salinity consumption, membrane fouling mitigation, and sludge dewatering enhancement.

Except for exploring the performance of in-situ UV/E-Cl assisted FO process, it is critical to delve into the underlying mechanisms governing membrane fouling mitigation to fully realize the potential of this integrated system. This might be challenging due to the inherent complexity of the membrane fouling process and the lack of efficient tools. Indeed, overall membrane fouling can be simplified as a sequential process involving foulant adhesion and subsequent filtration through the foulant layer^25,26^. Foulant adhesion is a thermodynamic process that can be quantified using the extended Derjaguin-Landau-Verwey-Overbeek (XDLVO) theory to assess the interactions between foulants and the membrane surface^27,28^. Additionally, recent studies have indicated that the filtration process also possesses a thermodynamic nature and can be described by the extended Flory-Huggins lattice theory from the perspective of chemical potential changes during filtration through a gel layer^25,29^.

Here, we propose a novel in-situ UV/E-Cl assisted FO process for targeted control of gel-like membrane fouling during WAS dewatering. Combined with a series of physicochemical property characterizations, a unified thermodynamic framework consisting of XDLVO and extended Flory-Huggins lattice theory was introduced to clarify the correlation between foulant properties and fouling behaviors, thereby providing comprehensive insights into the membrane fouling mechanism. This work could deepen our understanding of gel fouling behaviors during FO process, and provide technical clues and necessary theoretical basis to optimize WAS dewatering in practical FO applications.

## Results

### Feasibility of reverse Cl^−^ driven in-situ UV/E-Cl

**Fig. 1:**
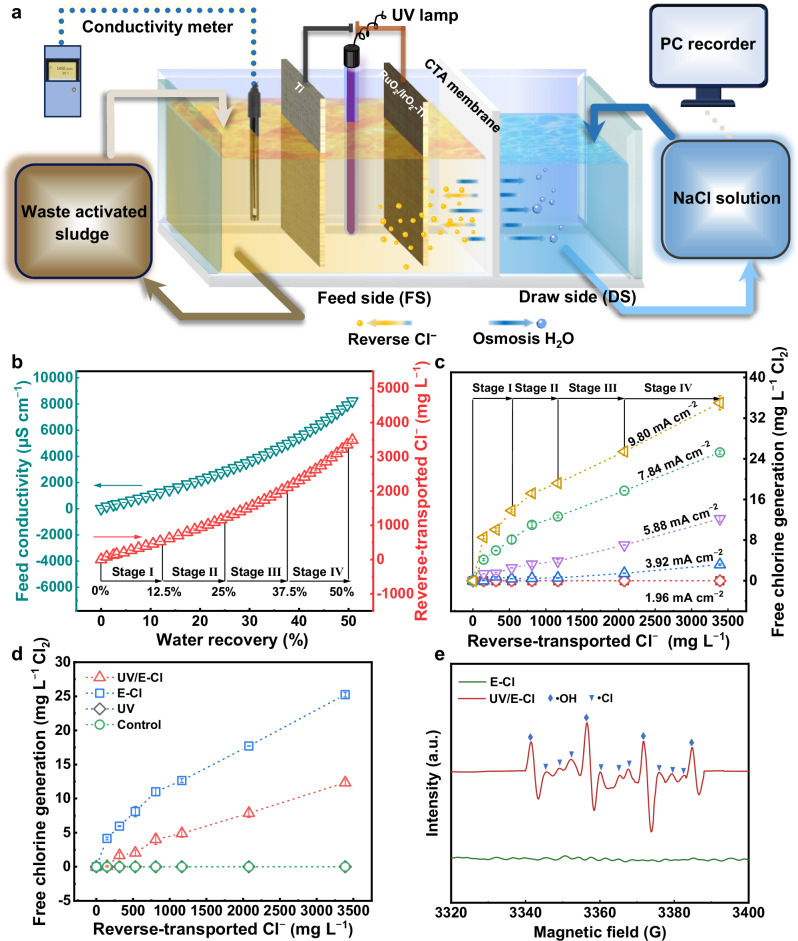
Feasibility analysis of in-situ UV/E-Cl process driven by dynamic reverse Cl^−^ in FO system. **a** Schematic diagram of the in-situ UV/E-Cl assisted FO system. A comprehensive description of the integrated components and their arrangement is provided in Supplementary Text 1. Four experimental groups were defined: control (no treatment), UV (UV irradiation alone), E-Cl (electrooxidation alone), and UV/E-Cl (combined process). **b** Change in feed conductivity and reverse Cl^−^ concentration with increasing water recovery during FO process. **c** Free chlorine generation during E-Cl process at current densities from 0 to 9.80 mA·cm^−2^, under corresponding reverse Cl^−^ concentrations. **d** Comparison of free chlorine generation during UV/E-Cl, E-Cl, control, and UV processes at a current density of 7.84 mA·cm^−2^. **e** EPR spectra of different radicals during E-Cl and UV/E-Cl processes, with ◊ and **▽** representing the typical peak profile of HO• and Cl•, respectively. Reaction conditions: working volume = 500 mL, duration = 10 min, UV power = 10 W, UV fluence rate = 0.44 mW·cm^−2^. Error bars denote standard deviation. Source data are provided as a Source Data file.

Figure 1a illustrates the schematic diagram of in-situ UV/E-Cl assisted FO system. During FO process, feed conductivity increased continuously, corresponding to an increase in reverse Cl^−^ concentration from 0 to 554, 1182, 2079 and 3371 mg·L^−1^, observed in stages I (0–12.5% water recovery), II (12.5–25% water recovery), III (25–37.5% water recovery) and IV (37.5–50% water recovery), respectively (Fig. 1b). The Cl^−^ accumulated in the feed is considerable enough to support free chlorine generation (Eqs. 1, 2) compared with conventional E-Cl process involving ∼100-600 mg·L^−1^ Cl^−^ ^24^. Free chlorine generation increased with increasing Cl^−^ concentration (0–3371 mg·L^−1^) and current densities (0–9.80 mA·cm^−2^) (Fig. 1c), indicating that Cl^−^ anodic oxidation to Cl_2_ was mass transfer limited, and that this sustained reverse Cl^−^ benefited free chlorine accumulation. However, a marginal dependency of Cl_2_ generation occurred at a higher current density of 9.80 mA·cm^−2^ owing to the accentuated side reaction with unlimited water^30^. Therefore, the current density of 7.84 mA·cm^−2^ was chosen for subsequent experiments. Apparently, the UV and control could not generate free chlorine (Fig. 1d). Compared with E-Cl, a ~ 60% chlorine decrease was observed during the UV/E-Cl process, suggesting that ∼60% of the free chlorine was subsequently photolyzed by UV irradiation. As confirmed by electron paramagnetic resonance (EPR) signal with typical thirteen-line spectra (Fig. 1e), Cl• and HO• were formed (Eqs. 3, 4)^31^. These results suggest that reverse Cl^−^ is feasible for Cl• generation during FO process.

1 $$2{{{{\rm{Cl}}}}}^{-}-2{{{{\rm{e}}}}}^{-}\to {{{\rm{C}}}}{{{{\rm{l}}}}}_{2}$$

2 $${{{\rm{C}}}}{{{{\rm{l}}}}}_{2}+{{{{\rm{H}}}}}_{2}{{{\rm{O}}}}\to {{{\rm{HOCl}}}}+{{{\rm{O}}}}{{{{\rm{Cl}}}}}^{-}+{{{{\rm{H}}}}}^{+}+{{{{\rm{Cl}}}}}^{-}$$

3 $${{{\rm{HOCl}}}}+{{{\rm{hv}}}}\to {{{\rm{HO}}}}{{\bullet }}+{{{\rm{Cl}}}}{{\bullet }}$$

4 $${{{\rm{O}}}}{{{{\rm{Cl}}}}}^{-}+{{{\rm{hv}}}}\to {{{{\rm{O}}}}}^{-}+{{{\rm{Cl}}}}{{\bullet }}$$

### WAS dewatering performance and EPS physicochemical properties analysis

**Fig. 2:**
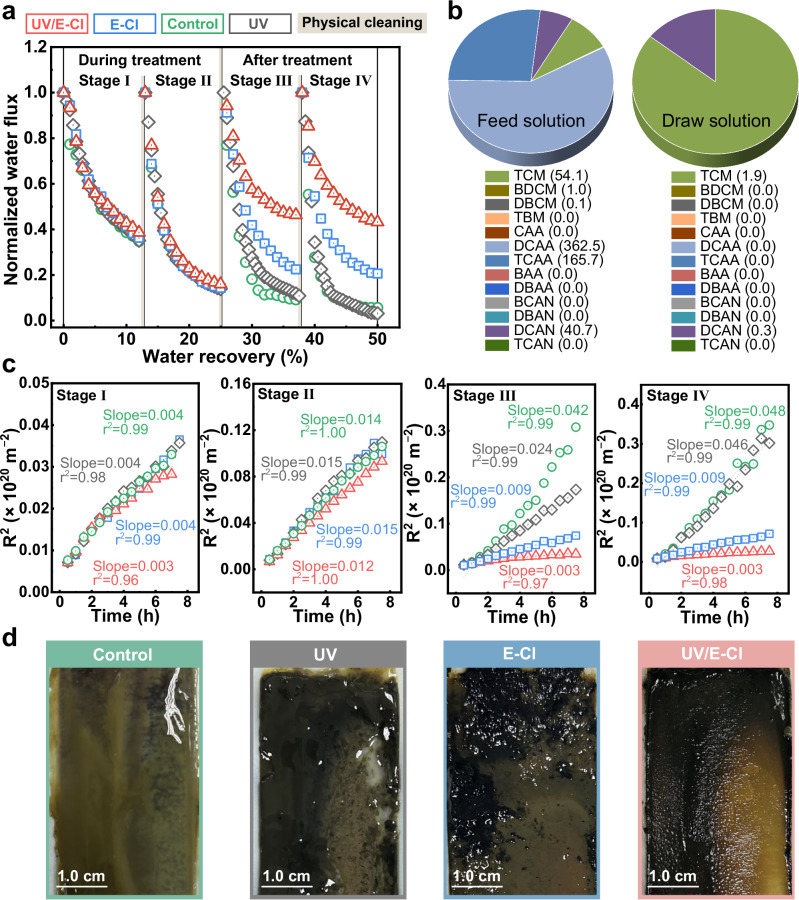
WAS dewatering performance assessment. **a** Normalized water flux with water recovery. Note that physical membrane cleaning was performed after each stage due to severe water flux decline in the control and UV processes. **b** DBPs concentrations in the feed and draw solutions. HANs: bromochloroacetonitrile (BCAN), dibromoacetonitrile (DBAN), dichloracetonitrile (DCAN), and trichloroacetonitrile (TCAN). THMs: chloroform (TCM), bromodichloromethane (BDCM), chlorodibromomethane (DBCM), and bromoform (TBM). HAAs: monochloroacetic acid (CAA), dichloroacetic acid (DCAA), trichloroacetic acid (TCAA), monobromoacetic acid (BAA), and dibromoacetic acid (DBAA). **c** Cake filtration model fitting for water flux, where *R* represents filtration resistance. **d** Direct observation of foulant layers. Experimental conditions: draw solution = 4 M NaCl, current density = 7.84 mA·cm^−2^, UV power = 10 W, UV fluence rate = 0.44 mW·cm^−2^, and exposure mode = 5’-ON/25’-OFF. Source data are provided as a Source Data file.

Details about the WAS source and characteristics are provided in Supplementary Table 1. The UV/E-Cl-assisted FO process exhibited a substantial improvement in dewatering flux (Supplementary Eq. 1), with significant average increases of 113%, 544%, and 514% in stages III and IV compared to the E-Cl, UV, and control systems, respectively (Fig. 2a). The limited performance in stages I and II was likely attributed to the insufficient reverse Cl^−^ for timely generation of free radicals (Fig. 1d)^24,31^. Moreover, the considerable WAS dewatering performance achieved using 1 M NaCl draw solution suggested the broad applicability of the UV/E-Cl-assisted FO process (details in Supplementary Fig. 1). To assess membrane performance after UV/E-Cl cycles, deionized water filtration and structural characterization were performed. Initial water flux and reverse salt accumulation were comparable for both pristine and used membranes (Supplementary Fig. 2a), and Fourier Transform Infrared (FTIR)/Scanning Electron Microscope (SEM) analyses revealed no tangible structural alterations (Supplementary Fig. 2b–f), indicating that the membrane integrity was not compromised by UV/E-Cl. The result is possibly attributable to the high organic loading protecting the membrane by acting as a free radical scavenger. The formation of chlorinated disinfection byproducts (DBPs) during WAS dewatering with UV/E-Cl was investigated (detailed methods in Supplementary Text 2.3). Negligible DBPs concentrations in the draw solution indicated effective DBPs rejection by the FO membrane (Fig. 2b draw solution). The dewatered feed sludge contained 55.1 μg·L^−1^ regulated trihalomethanes (THMs), 528.2 μg·L^−1^ regulated haloacetic acids (HAAs), and 40.7 μg·L^−1^ haloacetonitriles (HANs) (Fig. 2b feed solution). THMs and HANs were below WHO drinking water guidelines of 560 μg·L^−1^ and 90 μg·L^−1^, respectively^32^. Despite HAAs surpassed the 270 μg·L^−1^ drinking water guideline^33^, subsequent anaerobic digestion (removing 68.68% DBPs) is deemed sufficient to mitigate environmental risks^34^.

Fluxes were analyzed with a cake filtration model (Supplementary Eq. 3) to further evaluate membrane fouling behaviors. UV/E-Cl process achieved the greatest reduction in filtration resistances by orders of magnitude, with *R*^2^_stageIII_ = 3.04 × 10^18^ and *R*^2^_stageIV_ = 2.71 × 10^18^ (Fig. 2c), corresponding to slight resistance rates of 0.003 and 0.003 m^−1^, respectively. The excellent fits to cake filtration model (*r*² > 0.96) indicated that foulants were deposited on the membrane surface for all processes, without concern for membrane pores clogging. These results indicate that UV/E-Cl effectively mitigates membrane fouling during WAS dewatering process and suggest that understanding the mitigation mechanisms should focus on the foulant behaviors at the membrane surface. As shown in Fig. 2d, the trend of membrane fouling alleviation coincided with a significant transformation of the foulant layer from a highly hydrated, viscous, gel-like structure to a more particulate and drier cake-like layer. This observation aligned with the established literature^5–7,35,36^ consensus that, rather than suspended biological particles, organic polymers with hydrated colloidal characteristics, particularly EPS, primarily determine membrane fouling. Therefore, further EPS characterizations were focused on to provide clues for in-depth fouling behavior analysis.

**Fig. 3:**
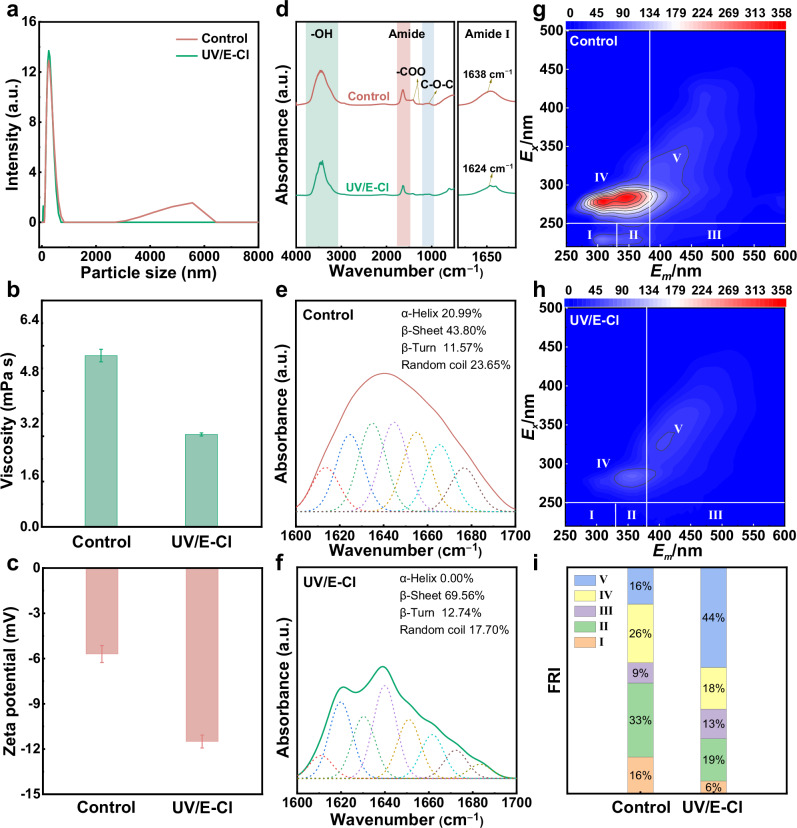
Effects on physicochemical properties of EPS. **a** Particle size distribution. **b** Apparent viscosity. **c** Zeta potential, **d** FTIR absorbance spectra (4000–500 cm^−1^). **e, f** Second derivatives resolution-enhanced curve-fitted deconvolved amide I spectra (1700–1600 cm^−1^) for the control and UV/E-Cl, respectively. Amide I secondary structure assignments: α-helix (1651–1660 cm^−1^), β-sheet (1618–1640 cm^−1^), random coil (1641–1650 cm^−1^), and β-turn (1667–1685 and 1691–1696 cm^−1^)^62^. **g, h** EEM spectra of the control and UV/E-Cl, respectively. Regions I and II (*E*_x_ < 250 nm, *E*_m_ < 350 nm) are associated with simple aromatic proteins such as tyrosine and tryptophan^63^. Region III (200 nm<*E*_x_ < 250 nm, *E*_m_ > 380 nm) represents fulvic acid-like substances^64^. Region IV (250 nm<*E*_x_ < 280 nm, *E*_m_ < 380 nm) is related to soluble microbial by-product-like material (including microbial polysaccharides)^65,66^. Region V (*E*_x_ > 280 nm, *E*_m_ > 380 nm) is related to humic acid-like organics^64^. **i** Fluorescence regional integration (FRI) distribution. Experimental conditions: draw solution = 4 M NaCl, current density = 7.84 mA·cm^−2^, UV power = 10 W, UV fluence rate = 0.44 mW·cm^−2^, and exposure mode = 5’-ON/25’-OFF. Error bars denote standard deviation. Source data are provided as a Source Data file.

EPS extraction and analysis were conducted on the concentrated sludge of the UV/E-Cl and control. The decrease in particle size from 471 nm to 272 nm (Fig. 3a), reduction in apparent viscosity from 5.30 mPa·s to 2.86 mPa·s (Fig. 3b), and substantial drop in zeta potential from −5.70 mV to −11.50 mV (Fig. 3c) indicated that UV/E-Cl strongly disrupted the structure of the EPS matrix. FTIR spectra provided insights into the EPS composition (Fig. 3d). The absorption bands at 1638 cm^−1^ and 1520 cm^−1^ correspond to the CO-NH group of amide I (C = O and C-N stretching) and amide II (N-H bending and C-N stretching) in proteinaceous material, respectively^37,38^. The peak at 1125 cm^−1^ signifies C-O-C vibrations in polysaccharides^37,38^. Notably, following treatment, the amide II band disappeared, and the amide I band exhibited a decrease in intensity with a blueshift from 1638 cm^−1^ to 1624 cm^−1^ (see enlargement of amide I for details). Amide I fitting revealed the disappearance of α-helix structure and a decrease in the α-helix/ (β-sheet + random coil) ratio from 31.12% to 0.00% (Fig. 3e, f). These changes strongly suggest that the protein fraction in the EPS underwent partial backbone disruption and helical unfolding^39^. Similarly, the observed decrease in band intensity of the C-O-C peak suggests that the structural integrity of polysaccharides was also compromised. The finding was further supported by excitation-emission matrix (EEM) spectroscopy, where the fluorescence of proteins-like and polysaccharide-like characteristic regions exhibited lower intensities (regions I (16% to 6%), II (33% to 19%) and IV (26% to 18%)) **(**Fig. 3g–i**)**.

UV/E-Cl process effectively disrupted the physical structure and chemical composition of EPS by targeting its major components—proteins and polysaccharides. A thorough understanding of these protein and polysaccharide fractions is crucial for unraveling the detailed membrane fouling influential mechanisms. However, the inherent structural complexity and variability of these components^40^ have severely limited our ability to gain insights into the membrane fouling behaviors at a more microscopic level, including their molecular-level interactions, their synergistic membrane fouling behavior, their functional role in hydration state differences, and thermodynamic simulations of whole-membrane fouling processes. Given the current limitations in accurately resolving the precise structures of proteins and polysaccharides, the use of model pollutants has emerged as an effective strategy for revealing general mechanisms^41–43^. Bovine serum albumin (BSA) and sodium alginate (SA) are frequently used as models for proteins and polysaccharides in EPS, respectively, due to their comparable structural properties and fouling behaviors^44,45^.

## Discussion

Dewatering experiments were further conducted using a combined solution of SA and BSA (SA-BSA mixtures, preparation details in Supplementary Text 3) as model foulant. Optimal water flux was achieved in stages III and IV, with stabilized fluxes approaching 138% of the E-Cl, 239% of the UV, and 198% of the control (Supplementary Fig. 3a). Moreover, all processes fitted the cake filtration model well (*r*^2^ > 0.92), and filtration resistances decreased significantly by orders of magnitude to *R*^2^_stageIII_ = 0.45 × 10^18^ and *R*^2^_stageIV_ = 0.38 × 10^18^ during UV/E-Cl (Supplementary Fig. 3b). The similar dewatering performance observed with both the SA-BSA mixtures and actual WAS under UV/E-Cl suggests the dominant role of proteins and polysaccharides in WAS dewatering behaviors, and demonstrates the relevance of SA-BSA model in adequately simulating the complex behavior of EPS under realistic dewatering scenarios.

**Fig. 4:**
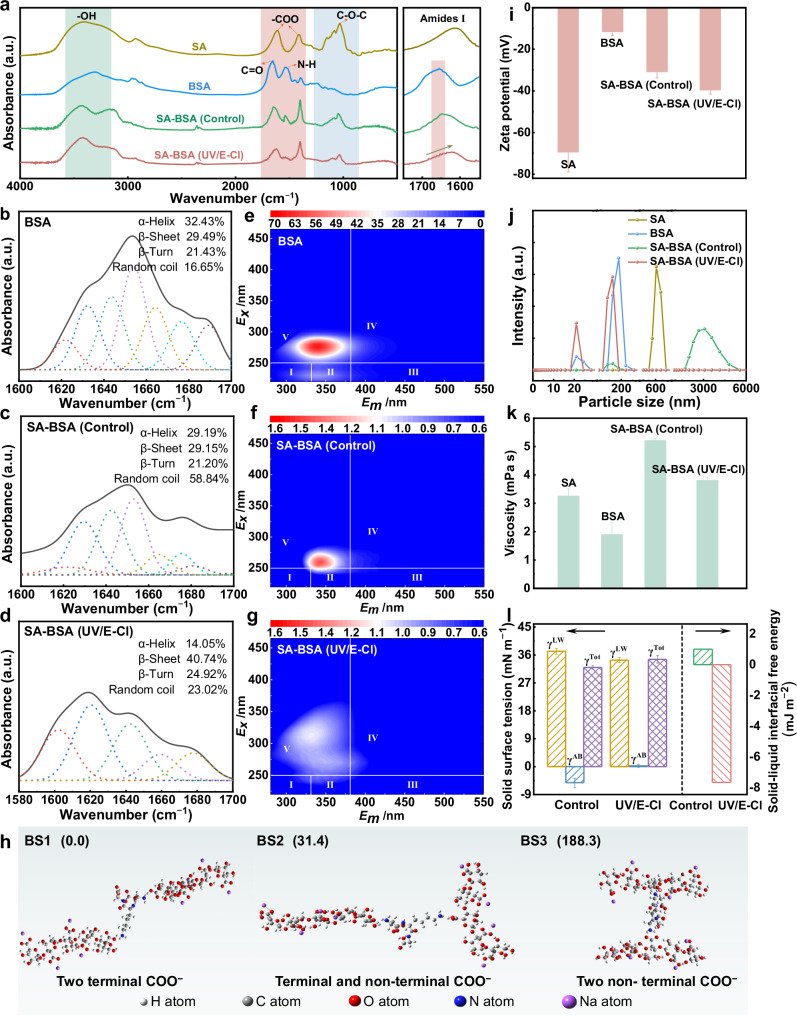
Molecular-level insights into proteins and polysaccharides using an SA-BSA model system. **a** FTIR spectra (4000–500 cm^−1^) of pure SA, pure BSA, SA-BSA mixtures in the control and SA-BSA mixtures in the UV/E-Cl. Second derivative resolution-enhanced curve-fitted deconvolved amide I spectra (1700–1600 cm^−1^) for **b** pure BSA, **c** SA-BSA mixtures in the control, and **d** SA-BSA mixtures in the UV/E-Cl. EEM spectra of **e** pure BSA, **f** SA-BSA mixtures in the control, and **g** SA-BSA mixtures in the UV/E-Cl. **h** DFT-optimized three binding models between SA and BSA chains, with calculated relative energies (kcal·mol^−1^) given in parentheses. **i** Zeta potential. **j** Particle size distribution. **k** Apparent viscosity. **l** Solid-phase surface tension (*γ*^*AB*^ is Lewis acid-base force, *γ*^*LW*^ is van der Waals force, *γ*^*Tot*^ is the sum of *γ*^*AB*^ and *γ*^*LW*^) and solid-liquid interfacial free energy analysis. Experimental conditions: feed solution = SA-BSA mixtures ([SA]_0_ = [BSA]_0_ = 500 mg·L^−1^), draw solution = 4 M NaCl, current density = 7.84 mA·cm^−2^, UV power = 10 W, UV fluence rate = 0.44 mW·cm^−2^, and intermittent exposure mode = 5’-ON/25’-OFF. Error bars denote standard deviation. Source data are provided as a Source Data file.

We first investigated the intermolecular interaction between BSA and SA to gain clues about their high membrane fouling behaviors. FTIR spectra provided further micro-level characterization of SA and BSA. Specifically, as detailed in the band assignments with specific wavenumbers, SA-BSA mixtures showed increased intensity in the -COO symmetric stretching, decreased intensity in the N-H bending, and a higher ratio of amide I to II (from 1.06 to 1.17) compared to pure SA and BSA (Fig. 4a). These spectral changes are indicative of electrostatic bridging between the amino groups (NH₃⁺) of BSA and the carboxyl groups (COO⁻) of SA^46^. Further support for the interaction between SA and BSA was provided by a partial conformational deformation of BSA. This was reflected by discernible differences in secondary structure shape and distribution^47^, and by shifts in EEM peak positions and intensities between the SA-BSA mixtures **(**Fig. 4c, f) and pure BSA (Fig. 4b, e). Density functional theory (DFT) simulations identified three molecular-level binding modes between SA and BSA, involving coordination of BSA NH_3_^+^ with SA terminal COO^−^ (BS1), with both terminal and non-terminal COO^−^ (BS2), or with non-terminal COO^−^ (BS3) (Fig. 4h). The relative energies (BS1 < BS2 < BS3) indicated that the linear conformation of BS1 was energetically preferred, which favors the elongation and subsequent cross-linking of polymer chains^48^. Consequently, SA-BSA mixtures showed a zeta potential (−30.89 mV) intermediate between pure SA and BSA (Fig. 4i), with a considerably larger floc size (~3053 nm) (Fig. 4j), and a higher apparent viscosity (5.21 mPa·s) (Fig. 4k), which, theoretically, enables the effective mechanical entrapment of water molecules^49^, and the subsequent formation of a highly hydrated, gel-like layer (as visualized in Fig. 2d).

UV/E-Cl primarily targeted the structural characteristics of proteins and polysaccharides, rather than mineralization, as confirmed by the limited reduction in dissolved organic carbon (DOC) (Supplementary Fig. 4). In more detail, UV/E-Cl effectively decomposed their bridging structures by attacking monomers and weakening their interactions, with FTIR result showing a loss of characteristic skeletal structures (C-O-C and CO-NH) and bridging mediators (-COO and N-H) (Fig. 4a). To determine the contributions of HO• and Cl• in UV/E-Cl degradation of BSA and SA (Fig. 1e), nitrobenzene (NB) and benzoic acid (BA) were used as radical probes to assess competition kinetics (Supplementary Text 2.6 and Supplementary Fig. 5)^50^. Results indicated that Cl• contributed over 90% to both BSA and SA degradation (Supplementary Table 2). The secondary-order rate constant for Cl• with SA (*k*_Cl•, SA_) was ∼2.76 × 10^10 ^M^–1^ s^–1^, and for Cl• with BSA (*k*_Cl•, BSA_) was ∼1.2 times of *k*_Cl•, SA_, at ∼3.36 × 10^10^ M^–1^ s^–1^. These high rates are comparable to the reaction of Cl• with other organics, such as aniline (4.0 × 10^10 ^M^–1^ s^–1^)^51^, indicating its efficiency for proteins and polysaccharides degradation. By comparison, the higher reactivity of Cl• towards proteins suggests a greater role for proteins in membrane fouling mitigation. As a result, UV/E-Cl extensively fragmented SA-BSA mixtures into smaller particles (~20 nm or 165 nm) (Fig. 4j) and reduced viscosity (3.80 mPa·s) (Fig. 4k). This fragmentation was more negative zeta potential (−39.63 mV) (Fig. 4i) due to the liberation of -COO^−^ groups following the disruption of intermolecular interactions. This, in turn, facilitated the dispersion of EPS fragments, and the release of trapped water molecules. Additionally, the fragments exhibited increased interfacial hydrophobicity (negative solid-liquid interfacial free energy), which was linked to a reduced solid-phase polarity (positive shift and value decrease in surface tension polar item (*γ*^*AB*^))^52^ **(**Fig. 4l**)**. This loss of polarity can be attributable to reduced hydrophilic groups (O-H, -COO and N-H), as observed in FTIR analysis (Fig. 4a), and the exposure of internal hydrophobic sites due to partial protein unfolding, which was reflected in secondary structure changes and fluorescence peak blueshift (from 342 nm to 333 nm) (Fig. 4d, g)^39^.

Overall, UV/E-Cl treatment facilitated the release of both interstitial water mechanically trapped in the bridging structures and interfacial water attached to the hydrophilic sites to weaken solid-phase water-holding capacity, which is theoretically beneficial in mitigating gel-like fouling^53^. To provide quantitative evidence for the synergistic membrane fouling behavior of proteins and polysaccharides and further clarify underlying fouling mitigation mechanisms, a comprehensive investigation into the fouling behaviors of SA-BSA mixtures in terms of adhesion and filtration processes on the membrane surface is required.

### Adhesion behaviors on the membrane surface

**Fig. 5:**
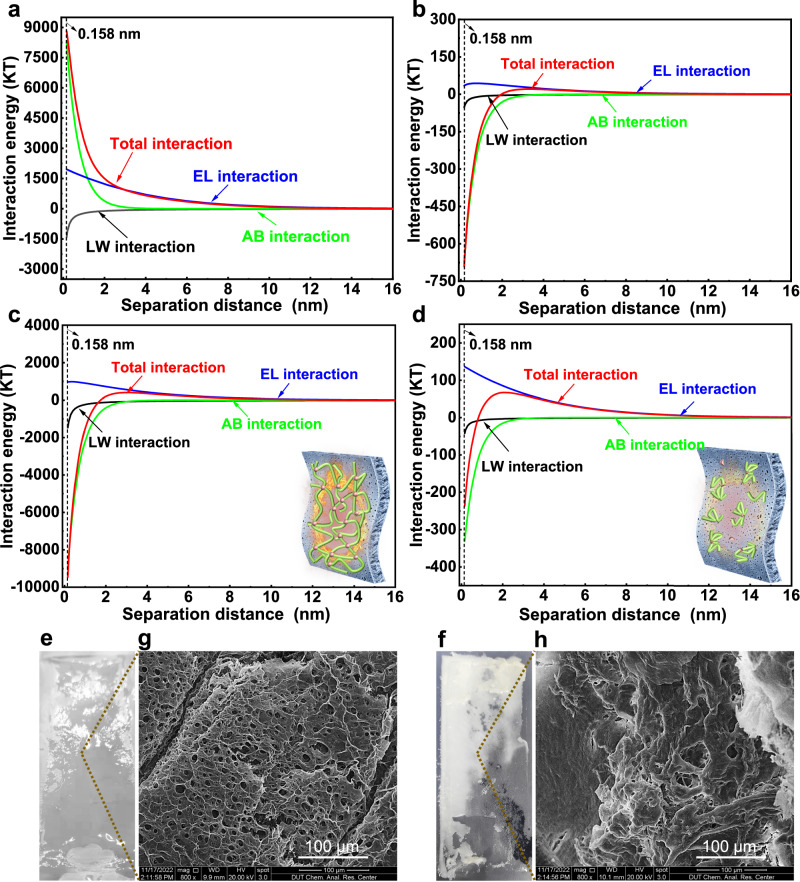
Interfacial adhesion processes characterization. **a, b** Cohesive energy within SA-BSA mixtures in the control and UV/E-Cl, respectively. **c, d** Adhesion energy of SA-BSA mixtures on the membrane active layer in the control and UV/E-Cl, respectively. **e, f** Direct observations of foulant layers in the control and UV/E-Cl, respectively. **g**, **h** SEM images of foulant layers in the control and UV/E-Cl, respectively. Source data are provided as a Source Data file.

Firstly, we evaluated cohesive interactions within the SA-BSA mixtures after stage II using surface characteristics data (Supplementary Table 3). Total cohesive energies were dominated by AB interaction, and there was a shift from repulsion (8812.85 kT, Fig. 5a) to attraction (−690.41 kT, Fig. 5b) after UV/E-Cl treatment. This suggests that hydrophobic interactions dominate the occurrence of solids self-aggregates or even precipitation, which might weaken foulants deposition tendency on the membrane surface, unlike SA-BSA mixtures in the control with extended bridging structure that can hook onto the membrane surface like a tentacle. Adhesion energy in UV/E-Cl (−235.95 kT, Fig. 5d) was only 2.49% of the control (−9457.92 kT, Fig. 5c). Due to the similarity in zeta potential and contact angle parameters, the poor foulants adhesion to membranes in UV/E-Cl can be attributed to particle size as small as 3.05% of SA-BSA mixtures in the control^54^ (Fig. 4j) (Eqs. 9–11).

Direct observation of foulant layers showed a hydrated state in the control (Fig. 5e), while dry and rough in UV/E-Cl (Fig. 5f). The difference in appearance was strongly consistent with the hydration state shifts of the foulant layers observed in WAS experiments (Fig. 2d). SEM identified a uniform cross-linked network structure in the control (Fig. 5g), corroborating the above speculation of DFT simulation results (Fig. 4h). Conversely, UV/E-Cl treatment significantly disrupted the network into a block-fragmented and stacked arrangement (Fig. 5h). Clearly, both the foulant layers in the control and UV/E-Cl possess rich pore channels for water outflow, which contradicts the order-of-magnitude difference in filtration resistance (Fig. 2c). This highlights the need for further investigation into the filtration behaviors through these pores to understand the underlying, more complex fouling mechanism^25,26^.

### Filtration behaviors through the foulant layer

**Fig. 6:**
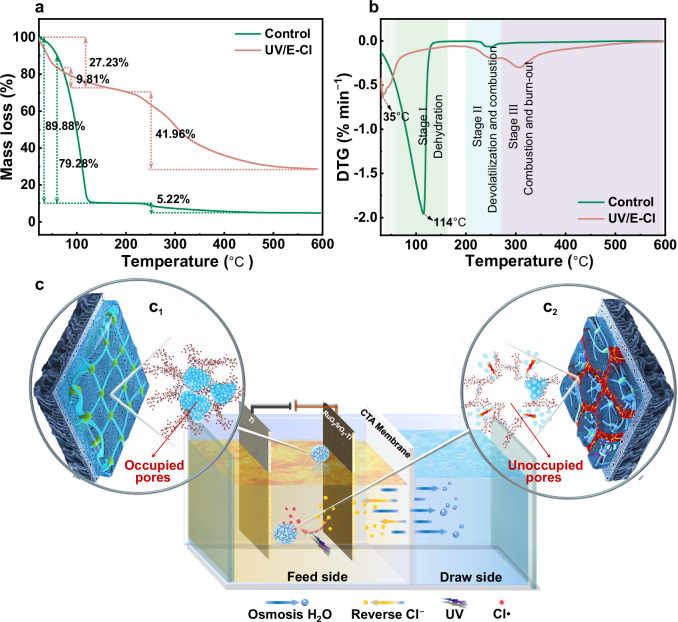
Identification of water occurrence states within the pores of foulant layers. **a** TGA and **b** DTG analysis of foulant layers. **c** Corresponding schematic diagram of foulant layers states. Subscripts 1 and 2 represent the control and UV/E-Cl, respectively. Source data are provided as a Source Data file.

Considering the intrinsic water phase surrounding the solid microenvironment in the hydrated foulant layer, it is necessary to focus on water occurrence states. As depicted in thermogravimetric analysis (TGA), the hydrated foulant layer water content was 89.88% in the control, while in the UV/E-Cl system it was only 27.23% (Fig. 6a). Derivative thermogravimetry (DTG) showed that maximum water evaporated rates in the control and UV/E-Cl occurred at 114 °C and 35 °C, respectively (Fig. 6b). It has been proposed that free water is released from 40 °C to 60 °C, and bound water evaporation requires 60–160 °C^55^. Accordingly, bound water in the foulant layers occupied 79.28 and 9.81% in the control and UV/E-Cl, respectively. The aforementioned shift in water occurrence state was quantitatively confirmed with 87.63% of bound water converted to free water. Therefore, in the control, a homogeneous gel-like layer was formed with the pores occupied by abundant bound water (Fig. 6c1). After UV/E-Cl treatment, a loose and dried cake-like foulant layer with unfilled pores was formed (Fig. 6c2).

**Fig. 7:**
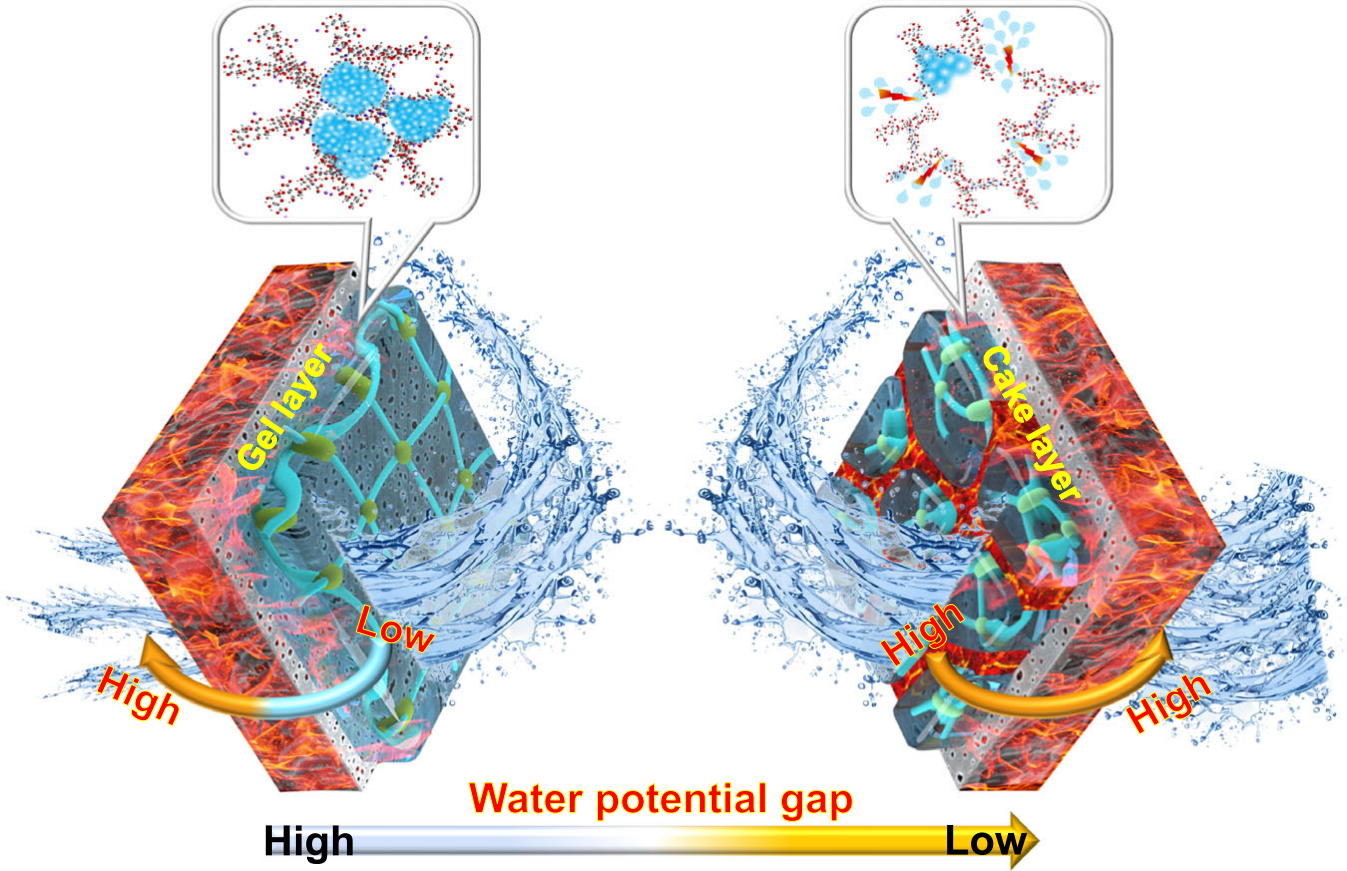
Schematic thermodynamic mechanism. Schematic thermodynamic mechanism underlying the membrane gel fouling mitigation by UV/E-Cl process.

Accordingly, the following thermodynamic mechanism underlying membrane fouling is proposed (Fig. 7). Based on traditional filtration theory—Carman-Kozeny equation, passing through a highly porous cake layer corresponds to a low hydraulic resistance^56^. Therefore, with UV/E-Cl treatment, copious free water released from the solid-phase can be driven by osmotic pressure difference (Δ*π*) to easily pass through the abundant connecting channels in the foulant layer, resulting in a lower filtration resistance. Clearly, traditional theory cannot describe the filtration process associated with the gel layer in which the pores are occupied by bound water, so we introduced Flory-Huggins lattice theory. This theory proposes that hydrophilic polymer hydration and swelling behaviors are spontaneous, accompanied by an increase in system entropy and a decrease in chemical potential^57,58^. The chemical potential variation (Δ*μ*) can be described as follows:

5 $$\Delta \mu={\left(\frac{\partial \Delta G}{\partial {n}_{1}}\right)}_{T,p,{n}_{2}}={rT}\left[{{{\mathrm{ln}}}}\left(1-{\varphi }_{2}\right)+\left(1-{N}^{-1}\right){\varphi }_{2}+\chi {\varphi }_{2}^{2}\right]$$

Where *N* is the degree of polymerization. For cross-linked polymers, *N* = ∞, and Δ*μ* tends to be rather high. Filtration through a gel layer can be viewed as an inverse gel formation process, in which bound water is dragged out of the gel into a free state, leaving the pores in polymers unoccupied. Such a sustainable filtration process relies on an extra force (Δ*P*) to offset the huge chemical potential gap (Δ*μ*) between the bound water with a low chemical potential and the free water with a high chemical potential^59^:

6 $$\Delta \mu=-V\Delta P$$

Where *V* is the solvent molar volume (m^3^·mol^−1^). Combining Eqs. 5, 6 with Supplementary Eq. 2, filtration resistance (*R*) associated with the chemical potential gap (Δ*μ*) is given by:

7 $$R=-\frac{\Delta \mu }{V\sigma J}=-\frac{{rT}\left[{{{\mathrm{ln}}}}\left(1-{\varphi }_{2}\right)+\left(1-{N}^{-1}\right){\varphi }_{2}+\chi {\varphi }_{2}^{2}\right]}{V\sigma J}$$

Table 1 lists the parameter values for theoretical *R* simulation. As calculated, theoretical *R* was 2.83 × 10^9 ^m^−1^, quite similar to the experimental *R* of 3.12 × 10^9 ^m^−1^ (accounting for 90.71%), suggesting that the chemical potential difference induced by dragging bound water into free water dominates filtration resistance. This simulation result provides conclusive thermodynamic evidence for the poor macroscopic filtration performance of the gel layer, and elucidates the fundamental mechanism underlying the membrane fouling mitigation with UV/E-Cl.

**Table 1:** Parameter values for *R* simulation

Parameter	Value	Unit	Source
*r*	8.314	kg m^2^ s^−2^ mol^−1^ K^−1^	theoretical value
*T*	298	K	experimental condition
*V*	1.80 × 10^−5^	m^3^ mol^−1^	theoretical value
*σ*	1.00 × 10^−3^	kg m^−1^ s^−1^	theoretical value
*J*	7.14 × 10^−7^	m^3^ m^−2^ s^−1^	experimental condition
*χ*	0.497	/	ref. ^61^
*φ*_*2*_	0.002	/	experimental condition

The proposed dynamic reverse Cl^−^-driven in-situ UV/E-Cl assisted FO process demonstrates exceptional performance in mitigating membrane gel fouling and enhancing WAS dewatering. This work provides quantitative thermodynamic evidence supporting the finding that “water occurrence states dominate membrane gel fouling”. To advance the process performance and facilitate the translation to larger-scale practical applications, future work should optimize reactor parameters to mimic realistic scenarios. This includes investigating the effects of electrode material and distance, UV intensity, and total effective treatment time, or assessing the impact of sludge composition, pH, and temperature. Exploring the synergistic effects of in-situ UV/E-Cl with other oxidants, like hydrogen peroxide cathodogenesis and free chlorine anodogenesis for free radical co-generation, is also valuable. For draw solution concentration, while 4 M NaCl improved dewatering by 62% over 1 M NaCl (Supplementary Fig. 6), primarily due to more Cl• precursor-Cl reverse accumulation (89.31%, Supplementary Fig. 7 and Supplementary Table 4), the readily available 1 M NaCl (seawater equivalent) offers a sustainable and cost-effective alternative given the resource demands of high-concentration NaCl. Strategic use of readily accessible high-salinity sources, such as reverse osmosis concentrates, is also an option.

Notably, although this work was carried out with WAS system, the recognition of water occurrence states as dominant holds universal significance for gel fouling mitigation and membrane processes operation, regardless of wastewater type, membrane model type, or reactor scale. Consequently, future applications of the UV/E-Cl assisted FO process could be expanded to various types of sludges, such as agricultural sludge and aquaculture sludge with high organic loads and substantial bound water content. Furthermore, the process could be explored for its potential in other systems, including algae solutions dewatering, waste leachate treatment, and agricultural wastewater treatment.

## Methods

### Materials and chemicals

Sodium alginate (SA) and bovine serum albumin (BSA) were obtained from Shanghai Aladdin Biochemical Technology Co., Ltd., and Beijing Solarbio Technology Co., Ltd., respectively. Sodium chloride (NaCl, 99.5%), sodium thiosulfate (Na_2_S_2_O_3_, 99%), nitrobenzene (NB, 99%), and benzoic acid (BA, 99%) were procured from Sinopharm Chemical Reagent Co., Ltd. 5,5-Dimethyl-1-pyrroline N-oxide (DMPO) was purchased from Shanghai Aladdin Biochemical Technology Co., Ltd. N,N-diethyl-p-phenylenediamine (DPD) reagent was sourced from Italy Hanna Instruments.

### Analytical methods

Water samples were withdrawn at predetermined time intervals and stored at 4 °C after rapid quenching by Na_2_S_2_O_3_ (Na_2_S_2_O_3_: Cl = 1). Please refer to Supplementary Text 2 for details on free chlorine (HOCl/OCl^−^) measurement, free radical identification, physicochemical characterization, DBPs measurement, EPS extraction procedures, DFT simulations, and competition kinetics analysis.

### Extended Derjaguin-Landau-Verwey-Overbeek (XDLVO) theory

According to XDLVO theory, the interaction energy balance for aqueous systems is expressed as follows^27,28^:

8 $${U}_{{{{{\rm{s}}}}}_{1}{{{\rm{w}}}}{{{{\rm{s}}}}}_{2}}^{{{{\rm{Tot}}}}}(h)={U}_{{{{{\rm{s}}}}}_{1}{{{\rm{w}}}}{{{{\rm{s}}}}}_{2}}^{{{{\rm{LW}}}}}(h)+{U}_{{{{{\rm{s}}}}}_{1}{{{\rm{w}}}}{{{{\rm{s}}}}}_{2}}^{{{{\rm{AB}}}}}(h)+{U}_{{{{{\rm{s}}}}}_{1}{{{\rm{w}}}}{{{{\rm{s}}}}}_{2}}^{{{{\rm{EL}}}}}(h)$$

Where *U*^Tot^ is the total interaction energy between two surfaces (s_1_ and s_2_) immersed in water environments (w) at a separation distance (*h*). When both s_1_ and s_2_ represent foulants, the interaction energy describes the cohesive energy between foulants. When s_1_ and s_2_ represent membrane and foulants, the interaction energy characterizes the adhesion tendency of foulants to the membrane surface; *U*^LW^, *U*^AB^, *U*^EL^ are the van der Waals interaction, Lewis acid-base interaction, and electrostatic interaction, respectively. The *U*^LW^, *U*^AB^, *U*^EL^ can be expressed as:

9 $${U}_{{{{{\rm{s}}}}}_{1}{{{\rm{w}}}}{{{{\rm{s}}}}}_{2}}^{{{{\rm{LW}}}}}(h)=2{{{\rm{\pi }}}}\Delta {G}_{{h}_{0}}^{{{{\rm{LW}}}}}\frac{{h}_{0}^{2}r}{h}$$

10 $${U}_{{{{{\rm{s}}}}}_{1}{{{\rm{w}}}}{{{{\rm{s}}}}}_{2}}^{{{{\rm{AB}}}}}(h)=2{{{\rm{\pi }}}}r\lambda \Delta {G}_{{h}_{0}}^{{{{\rm{AB}}}}}\exp \left[\frac{{h}_{0}-h}{\lambda }\right]$$

11 $${U}_{{{{{\rm{s}}}}}_{1}{{{\rm{w}}}}{{{{\rm{s}}}}}_{2}}^{{{{\rm{EL}}}}}(h)=2{{{\rm{\pi }}}}{\varepsilon }_{{{{\rm{r}}}}}{\varepsilon }_{0}r\left({\xi }_{{{{{\rm{s}}}}}_{1}}{\xi }_{{{{{\rm{s}}}}}_{2}}{{\mathrm{ln}}}\,\left(\frac{1+{{{{\rm{e}}}}}^{-\kappa h}}{1-{{{{\rm{e}}}}}^{-\kappa h}}\right)+\left({\xi }_{{{{{\rm{s}}}}}_{1}}^{2}+{\xi }_{{{{{\rm{s}}}}}_{2}}^{2}\right){{{\mathrm{ln}}}}\,\left(1-{{{{\rm{e}}}}}^{-2\kappa h}\right)\right)$$

Where *h*_*0*_ is the minimum distance between two surfaces, assumed to be 0.158 nm^60^, *r* is the foulants radius, *λ* is the characteristic decay length of the AB interaction, *ε*_*r*_*ε*_0_ denotes the solution permittivity, ${\xi }_{{{{{\rm{s}}}}}_{1}}$ and ${\xi }_{{{{{\rm{s}}}}}_{2}}$ are the surface zeta potentials, *κ* is the inverse Debye screening length, $\varDelta {G}_{{h}_{0}}^{{{{\rm{LW}}}}}$ and $\varDelta {G}_{{h}_{0}}^{{{{\rm{AB}}}}}$ are the LW and AB adhesion free energy per unit at the minimum distance *h*_0_, respectively.

12 $$\Delta {G}_{{h}_{0}}^{{{{\rm{LW}}}}}=-2\left(\sqrt{{\gamma }_{{{{{\rm{s}}}}}_{1}}^{{{{\rm{LW}}}}}}-\sqrt{{\gamma }_{{{{\rm{w}}}}}^{{{{\rm{LW}}}}}}\right)\left(\sqrt{{\gamma }_{{{{{\rm{s}}}}}_{2}}^{{{{\rm{LW}}}}}}-\sqrt{{\gamma }_{{{{\rm{w}}}}}^{{{{\rm{LW}}}}}}\right)$$

13 $$\Delta {G}_{{h}_{0}}^{{{{\rm{AB}}}}}=	2\left[\sqrt{{\gamma }_{{{{\rm{w}}}}}^{+}}\left(\sqrt{{\gamma }_{{{{{\rm{s}}}}}_{1}}^{-}}+\sqrt{{\gamma }_{{{{{\rm{s}}}}}_{2}}^{-}}-\sqrt{{\gamma }_{{{{\rm{w}}}}}^{-}}\right)+\sqrt{{\gamma }_{{{{\rm{w}}}}}^{-}}\left(\sqrt{{\gamma }_{{{{{\rm{s}}}}}_{1}}^{+}}+\sqrt{{\gamma }_{{{{{\rm{s}}}}}_{2}}^{+}}-\sqrt{{\gamma }_{{{{\rm{w}}}}}^{+}}\right) \right. \\ 	-\left.\sqrt{{\gamma }_{{{{{\rm{s}}}}}_{1}}^{-}{\gamma }_{{{{{\rm{s}}}}}_{2}}^{+}}-\sqrt{{\gamma }_{{{{{\rm{s}}}}}_{1}}^{+}{\gamma }_{{{{{\rm{s}}}}}_{2}}^{-}}\right]$$

Where *γ*^LW^, *γ*^*+*^ and *γ*^−^ are van der Waals, electron acceptor and electron donor components of surface tension, respectively (as detailed in Supplementary Text 4).

### Extended Flory-Huggins lattice theory

Flory-Huggins lattice theory was initially proposed by Paul Flory and Maurice Huggins to describe the thermodynamic changes during the formation of a polymer solution by mixing the pure polymer and pure water^57,58^. This theory assumes that solution is a rigid lattice frame in which molecules are arranged like crystals. Each solvent molecule occupies one lattice site, and a polymer occupies X connected lattice sites (X is the volume ratio of polymer to solvent molecule). Accordingly, the Gibbs free energy change (Δ*G*_mix_) during polymer solution formation can be described as:

14 $$\Delta {G}_{{mix}}=\Delta {H}_{{mix}}-T\Delta {S}_{{mix}}={rT}\left({n}_{1}{{\mathrm{ln}}}{\varphi }_{1}+{n}_{2}{{\mathrm{ln}}}{\varphi }_{2}+n{\varphi }_{2}\chi \right)$$

Where *r* and *T* are universal gas constant and absolute temperature, respectively. *χ* is Flory-Huggins interaction parameter. *n* and *φ* denote molar number and volume fraction, respectively. Subscripts 1 and 2 represent the solvent and polymer, respectively.

## Supplementary information

Supplementary Information

Transparent Peer Review file

## Data Availability

The data that supports the findings of the study are available within the manuscript, supplementary information, source data file, and from the corresponding authors upon request. Source Data file has been deposited in Open Science Framework under accession code 10.17605/OSF.IO/GJK26^67^.
