# Supplementary material for: Dynamic reverse Cl− driven integration of sludge conditioning and dewatering
Source: Nat Commun. 2025 Mar 19;16:2717. doi: 10.1038/s41467-025-57878-4 (PMC11923106; doi:10.1038/s41467-025-57878-4)
Supplement: Supplementary file 1 — Supplementary Information [file 41467_2025_57878_MOESM1_ESM.pdf]

Supplementary Information

Dynamic reverse Cl^- driven integration of sludge conditioning and dewatering

Xiujia You [1], Hanmin Zhang [1*], Hongjun Lin [2], Linhua Rao [1]

[1] Key Laboratory of Industrial Ecology and Environmental Engineering (Ministry of Education, MOE), School of Environmental Science and Technology, Dalian University of Technology, Dalian, 116024, China

[2] Key Laboratory of Watershed Earth Surface Processes and Ecological Security, College of Geography and Environmental Sciences, Zhejiang Normal University, Jinhua, 321004, China

[*] Corresponding author: zhhanmin@126.com

List of Contents

Supplementary Text 1. Components and arrangement of the in-situ UV/E-Cl assisted FO system 5

Supplementary Text 2. Supplementary analytical methods 6

Supplementary Text 3. SA-BSA solution preparation procedure 10

Supplementary Text 4. Determination of surface tension parameters 11

Supplementary Fig. 1. WAS dewatering performance assessment. a Normalized water flux with water recovery. **b** Direct observation of foulant layers. **c** Cake filtration model fitting for water flux, where *R* represents filtration resistance. Experimental conditions: draw solution = 1 M NaCl, current density = 7.84 $mA·cm^{-2}$, UV power = 10 W, UV fluence rate = 0.44 $mW·cm^{-2}$, and intermittent exposure mode = 5'-ON/25'-OFF. 12

Supplementary Fig. 2. Characterization of membrane integrity after UV/E-Cl cycles. a Comparison of water flux and feed conductivity between the pristine membrane and the used membrane after 4-cycle UV/E-Cl processes using deionized water as feed solution and 4 M NaCl as draw solution. **b** FTIR absorbance spectra (4000-500 cm^{-1}) for the selective layer and the support layer of the pristine membrane and the used membrane. SEM images showing **c-d** the selective layer of a pristine membrane and the used membrane, respectively, and **e-f** the support layer of a pristine membrane and the used membrane, respectively. 13

Supplementary Fig. 3. Dewatering performance validation using SA-BSA mixtures as model EPS. a Normalized water flux with water recovery. **b** Cake filtration model fitting for water flux, where *R* represents filtration resistance. Experimental conditions: feed solution = SA-BSA mixtures ($[SA]_0$ = $[BSA]_0$ = 500 $mg·L^{-1}$), draw solution = 4 M NaCl, current density = 7.84 $mA·cm^{-2}$, UV power = 10 W, UV fluence rate = 0.44 $mW·cm^{-2}$, and intermittent exposure mode = 5'-ON/25'-OFF. 14

Supplementary Fig. 4. DOC removal performance for SA-BSA mixtures. Experimental conditions: feed solution = SA-BSA mixtures ($[SA]_0$ = $[BSA]_0$ = 500

mg·L^{-1}), draw solution = 4 M NaCl, current density = 7.84 mA·cm^{-2}, UV power = 10 W, UV fluence rate = 0.44 mW·cm^{-2}, and intermittent exposure mode = 5’-ON/25’-OFF. 15

Supplementary Fig. 5. Identification of dominant reactive species during SA-BSA system. **a** Degradation kinetics of coexisting NB, BA, BSA and SA in the UV/E-Cl process refer to stage I. Conditions: 25 °C, $[HClO]_0$ = 0.30 mM, $[Cl^-]_0$ = 264 mg·L^{-1}, $[BSA]_0$ = $[SA]_0$ = 50 mg·L^{-1}, $[BA]_0$ = $[NB]_0$ = 0.01 mM, UV fluence rate = 0.44 mW·cm^{-2}. **b** Degradation kinetics of coexisting NB, BA, BSA and SA in the UV/E-Cl process refer to stage II. Conditions: 25 °C, $[HClO]_0$ = 0.60 mM $[Cl^-]_0$ = 823 mg·L^{-1}, $[BSA]_0$ = $[SA]_0$ = 50 mg·L^{-1}, $[BA]_0$ = $[NB]_0$ = 0.01 mM, UV fluence rate = 0.44 mW·cm^{-2}. **c** Competition kinetics between BA, BSA and SA in the UV/H_2O_2 system. Condition: 25 °C, $[H_2O_2]_0$ = 0.30 mM, $[Cl^-]_0$ = 264 mg·L^{-1}, $[BSA]_0$ = $[SA]_0$ = 50 mg·L^{-1}, $[BA]_0$ = $[NB]_0$ = 0.01 mM, UV fluence rate = 0.44 mW·cm^{-2}. **d** Competition kinetics between BA, BSA and SA in the UV/H_2O_2 system. Condition: 25 °C, $[H_2O_2]_0$ = 0.60 mM, $[Cl^-]_0$ = 823 mg·L^{-1}, $[BSA]_0$ = $[SA]_0$ = 50 mg·L^{-1}, $[BA]_0$ = $[NB]_0$ = 0.01 mM, UV fluence rate = 0.44 mW·cm^{-1}. 16

Supplementary Fig. 6. Comparison of WAS dewatering performance under different NaCl concentrations as draw solutions. Dewatering time required to achieve 50% water recovery using the UV/E-Cl process with a 1 M NaCl and b 4 M NaCl as draw solutions. Experimental conditions: current density = 7.84 mA·cm^{-2}, UV power = 10 W, UV fluence rate = 0.44 mW·cm^{-2}, and intermittent exposure mode = 5’-ON/25’-OFF. 17

Supplementary Fig. 7. Reverse-transported Cl^- concentration during 1 M NaCl and 4 M NaCl as draw solution. 18

Supplementary Table 1. Key characteristics of WAS collected from the secondary sedimentation of Xiajiahe wastewater treatment plant in Dalian, China. 19

Supplementary Table 2. Competition kinetics results during BSA and SA degradation by UV/E-Cl using NB and BA as radical probes. Conditions: 25°C, $[BSA]_0$ = $[SA]_0$ = 50 mg·L^{-1}, $[BA]_0$ = $[NB]_0$ = 0.01 mM. 20

Supplementary Table 3. The contact angles and zeta potentials of membrane and SA-BSA mixtures. 21

Supplementary Table 4. Evaluation of the specific role of NaCl in the experiments 22

Supplementary Table 5. Surface tension parameters of three probe liquids. 23

Supplementary Text 1. Components and arrangement of the in-situ UV/E-Cl assisted FO system

The electrochemical cell comprised a commercial ruthenium/iridium-oxide coated titanium (RuO_2/IrO_2-Ti, Fenggang, Suzhou) anode (3.0 cm × 8.5 cm), selected for its low cost and excellent selectivity for Cl^-, and a titanium (Ti, Fenggang) cathode (3.0 cm × 8.5 cm). A 10 W low-pressure UV lamp (GPH212T5L/4, Heraeus), emitting at 254 nm, was integrated into the system. The average UV fluence rate was measured to be 0.44 $mW{\cdot}cm^{-2}$ using iodide/iodate chemical actinometry[1]. The anode, cathode and UV lamp were positioned with a 1 cm gap along the centerline of the FS. A commercial cellulose triacetate (CTA) membrane (Fluid Technology Solutions, Inc.), chosen for its high chlorine resistance[2], was oriented with its active layer (0.0035 m^2 effective filtration area) toward the FS. Both the feed solution (2000 mL) and draw solution (1500 mL) were circulated at a linear velocity of 1.2 $cm{\cdot}s^{-1}$. A conductivity probe (Multi 3430, Germany) was located in the FS to continuously monitor conductivity and calculate the reverse Cl^- concentration[3]. The volume increase in DS was recorded via a PC connected to a digital scale.

Water flux (J, $L{\cdot}m^{-2}{\cdot}h^{-1}$) was calculated according to the following:

$$J = \frac{\Delta V}{A_m \Delta t} \tag{1}$$

Where ΔV (L) is the volume increment in DS at a period of time Δt (h), A_m (m^2) is the effective membrane surface area. The filtration resistance (R, m^{-1}) was evaluated according to Darcy's law[4]:

$$R = \frac{\Delta \pi}{\sigma J} \tag{2}$$

Where $\Delta\pi$ (bar) is the osmotic pressure difference between DS and FS; σ (Pa·s) is the dynamic viscosity of permeate. Plotting R vs. t could analyze fouling behaviors[5]:

$$R^2 = \frac{K_c \times \Delta \pi^2}{\sigma^2} t \tag{3}$$

Where K_c represents the cake filtration model constant.

Supplementary Text 2. Supplementary analytical methods

2.1 Free chlorine and radical measurement

Free chlorine ($HOCl/OCl^-$) was analyzed by a HANNA pocket colorimeters (HI96734, Italy) using N,N-diethyl-p-phenylenediamine (DPD) method[6]. Samples were collected at predetermined time intervals and tested after 2 minutes of gentle shaking with HI93734-01 reagent. Measurements were taken in triplicate. The free radicals were detected by an electron paramagnetic resonance (EPR) spectroscope (A200, Bruker) using 5,5-Dimethyl-1-pyrroline N-oxide (DMPO) as trapping agent.

2.2 Characterization methods

The dissolved organic carbon (DOC) concentration was measured by a total organic carbon (TOC) analyzer (Multi N/C 2100s, Analytik Jena). The hydrodynamic diameter and zeta potential of solution were measured by Malvern Zetasizer (Nano ZS90, Malvern). The solution viscosity was determined by a rotational viscosity meter (DV2T, Brookfield). The functional groups and fluorescent components were identified by FTIR (Nicolet 6700, ThermoFisher) and EEM spectrometer (F-7100, Hitachi), respectively. The microstructure of foulant layers deposited on membrane surface was analyzed by SEM (Quanta 450, FEI). The thermal stability of foulant layers was measured with a thermogravimetric analyzer (Q500, TA).

2.3 DBPs measurement

Feed and draw solutions were initially passed through solid-phase extraction columns to remove salts. For nitrogenous DBPs (N-DBPs), four haloacetonitriles (HANs, i.e., bromochloroacetonitrile (BCAN), dibromoacetonitrile (DBAN), dichloracetonitrile (DCAN), and trichloroacetonitrile (TCAN)) were targeted[7, 8]. For carbonaceous DBPs (C-DBPs), four regulated trihalomethanes (THMs, i.e., chloroform (TCM), bromodichloromethane (BDCM), chlorodibromomethane (DBCM) and bromoform (TBM)) and five regulated haloacetic acids (HAAs, i.e., monochloroacetic acid (CAA), dichloroacetic acid (DCAA), trichloroacetic acid (TCAA), monobromoacetic acid (BAA), and dibromoacetic acid (DBAA)) were targeted[9]. HANs (BCAN, DBAN, DCAN, and TCAN) and THMs (TCM, BDCM, DBCM and TBM) were quantified by purge and trap (Eclipse4760, OI) gas chromatography-mass

spectrometry (QP2010PLUS, Shimadzu) equipped with a capillary column (MXT-624 60 m × 0.25 mm × 1.4 μm, Shimadzu), based on USEPA Method 524.2[10]. HAAs (CAA, DCAA, TCAA, BAA, and DBAA) were measured by high-performance liquid chromatography-tandem mass spectrometry (HPLC/MS/MS) (LCMS-8050, Shimadzu) with an AQ-C18 column (2.1 mm × 50 mm, 1.9 μm, Shimadzu)[11].

2.4 EPS extraction procedures

A heat extraction method as described previously[12] was modified to extract EPS in WAS. The heating method was convenient with a relatively low cellular lysis and high extraction yield[13]. Sludge suspension sample of 25 mL was taken for analysis, and centrifuged at 8000 rpm for 5 min. The sludge pellet was re-suspended into 0.05% NaCl solution to its original volume, and then was heated to 60 °C in a water bath for 30 min. After that, the solution was centrifuged at 10,000 rpm for 15 min. The supernatant after filtration through 0.45 μm membrane was used as EPS solution.

2.5 Density functional theory (DFT) calculation

DFT simulations were performed to explore the interactions between SA and BSA for providing molecular level clues to membrane fouling behaviors. A single alginate chain consisting of 5 uronic rings and an amino acid fragment with a trimer of lysine, aspartic acid and glycine were constructed by GaussView 6.0 as the SA and BSA models, respectively[14, 15]. Molecular geometries between two alginate chains and a BSA fragment were optimized by B3LYP hybrid functional combined with 6-31G(d,p) basis set to obtain more accurate interaction energies. DFT-D3 dispersion corrections were taken into account in all calculations.

2.6 Competition kinetics analysis

To further distinguish the respective contributions of HO• and Cl• during UV/E-Cl process, radical probes, nitrobenzene (NB) and benzoic acid (BA), were employed for competition kinetics studies. UV/E-Cl processes were conducted in two typical cases, one with HClO and Cl^- concentrations of 0.30 mM and 264 $mg\cdot L^{-1}$ regarding stage I (Fig 1 b) and the other with HClO and Cl^- concentrations of 0.60 mM and 823 $mg\cdot L^{-1}$ regarding stage II (Fig 1 b). The initial concentrations of BSA, SA, NB and BA were 50 $mg\cdot L^{-1}$, 50 $mg\cdot L^{-1}$, 0.01 mM and 0.01 mM, respectively. The UV fluence rate

was 0.44 mW·cm^{-2}. The rate constants of BA reaction with HO• and Cl• are $k_{HO•,\ BA}$ = 5.9×10^9 M^{-1} s^{-1} and $k_{Cl•,\ BA}$ = 1.8×10^{10} M^{-1} s^{-1}, respectively. NB reacts rapidly with HO• with rate constants of $k_{HO•,\ NB}$ = 3.9×10^9 M^{-1} s^{-1}, but very slowly with Cl• ($k_{Cl•,\ NB} < 10^6$ M^{-1} s^{-1})[10, 16]. Accordingly, the steady-state concentrations of HO• ([HO•]$_{ss}$) and Cl• ([Cl•]$_{ss}$) during BSA and SA degradation by UV/E-Cl using NB and BA as radical probes can be given by:

$$k_{NB} = k_{HO•,NB}[HO•]_{ss} \quad (4)$$

$$k_{BA} = k_{HO•,BA}[HO•]_{ss} + k_{Cl•,BA}[Cl•]_{ss} \quad (5)$$

$$k_{BSA} = k_{HO•,BSA}[HO•]_{ss} + k_{Cl•,BSA}[Cl•]_{ss} \quad (6)$$

$$k_{SA} = k_{HO•,SA}[HO•]_{ss} + k_{Cl•,SA}[Cl•]_{ss} \quad (7)$$

where k_{NB}, k_{BA}, k_{BSA} and k_{SA} are the pseudo-first-order rate constants of the degradation of the coexisting NB, BA, BSA and SA in the UV/E-Cl process, respectively. The concentrations of BA and NB were analyzed using high performance liquid chromatography (HPLC, E2695, Waters, USA) equipped with a PDA detector[17]. SA concentration was determined by Waters high performance liquid chromatography (HPLC) system equipped with a TSK-gel G4000 PWXL column (7.8 mm I.D. × 300 mm) (Tosoh Bioscience, Tokyo, Japan)[18]. BSA concentration was determined based on Bradford assay[19].

To determine the second-order rate constant of HO• reacting with BSA ($k_{HO•,\ BSA}$) and with SA ($k_{HO•,\ SA}$), BA was chosen as a reference compound in the UV/H_2O_2 process, with the presence of NB to scavenge HO•. The UV/H_2O_2 process were carried out in two typical cases, one with H_2O_2 and Cl^- concentrations of 0.30 mM and 264 mg·L^{-1} set with reference to stage I and the other with H_2O_2 and Cl^- concentrations of 0.60 mM and 823 mg·L^{-1} set with reference to stage II. The initial concentrations of BSA, SA, BA and NB were 50 mg·L^{-1}, 50 mg·L^{-1}, 0.01 mM and 0.01 mM, respectively. The second-order rate constants of HO• with BSA and SA were calculated from Eqs. 8-9.

$$\ln\frac{[BSA]}{[BSA]_0}=\frac{k_{HO\bullet,BSA}}{k_{HO\bullet,BA}}\times\ln\frac{[BA]}{[BA]_0} \quad (8)$$

$$\ln\frac{[SA]}{[SA]_0}=\frac{k_{HO\bullet,SA}}{k_{HO\bullet,BA}}\times\ln\frac{[BA]}{[BA]_0} \quad (9)$$

Combining Eqs. 8-9 and Eqs. 6-7, the second-order rate constants of Cl• reacting with BSA ($k_{Cl\bullet, BSA}$) and with SA ($k_{Cl\bullet, SA}$) were derived.

Supplementary Text 3. SA-BSA solution preparation procedure

Generally, proteins and polysaccharides account for 7-32% of the mixed liquor suspended solids (MLSS) in sludge[20, 21]. Given that sludge MLSS typically ranges from 3000[22, 23] to 8000[23-25] $mg \cdot L^{-1}$, the total concentration of proteins and polysaccharides is expected to fall within the range of 210 to 2560 $mg \cdot L^{-1}$. In our work, the concentrations of proteins and polysaccharides were 609 ± 11.58 $mg \cdot L^{-1}$ and 583 ± 11.58 $mg \cdot L^{-1}$, respectively. Therefore, a SA-BSA solution with a total mass concentration of 1000 $mg \cdot L^{-1}$ and a mass ratio of 1:1 was prepared freshly as follows: 2000 mg SA and 2000 mg BSA powders were dissolved separately in 1.0 L deionized water with magnetic stirring at 100 rpm for at least 5 h. Then the above SA and BAS solutions were mixed and stirred at 50 rpm for 24 h to form a fully cross-linked SA-BSA mixture.

Supplementary Text 4. Determination of surface tension parameters

The surface tension components of foulants and membrane can be calculated by using extended Young's equation[26, 27]:

$$(1+\cos\theta)\gamma_w^{Tot} = 2\left(\sqrt{\gamma_s^{LW}\gamma_w^{LW}} + \sqrt{\gamma_s^{+}\gamma_w^{-}} + \sqrt{\gamma_s^{-}\gamma_w^{+}}\right) \tag{10}$$

$$\gamma^{Tot} = \gamma^{LW} + \gamma^{AB} \tag{11}$$

$$\gamma^{AB} = 2\sqrt{\gamma^{+}\gamma^{-}} \tag{12}$$

Where θ is the contact angle (°) measured by a contact angle goniometer (C602, Kino) using three probe liquids according to the standard sessile droplet method[28]. γ^{AB} is Lewis acid-base components of surface tension. Surface tension parameters of the probe liquids list in Supplementary Table 5.

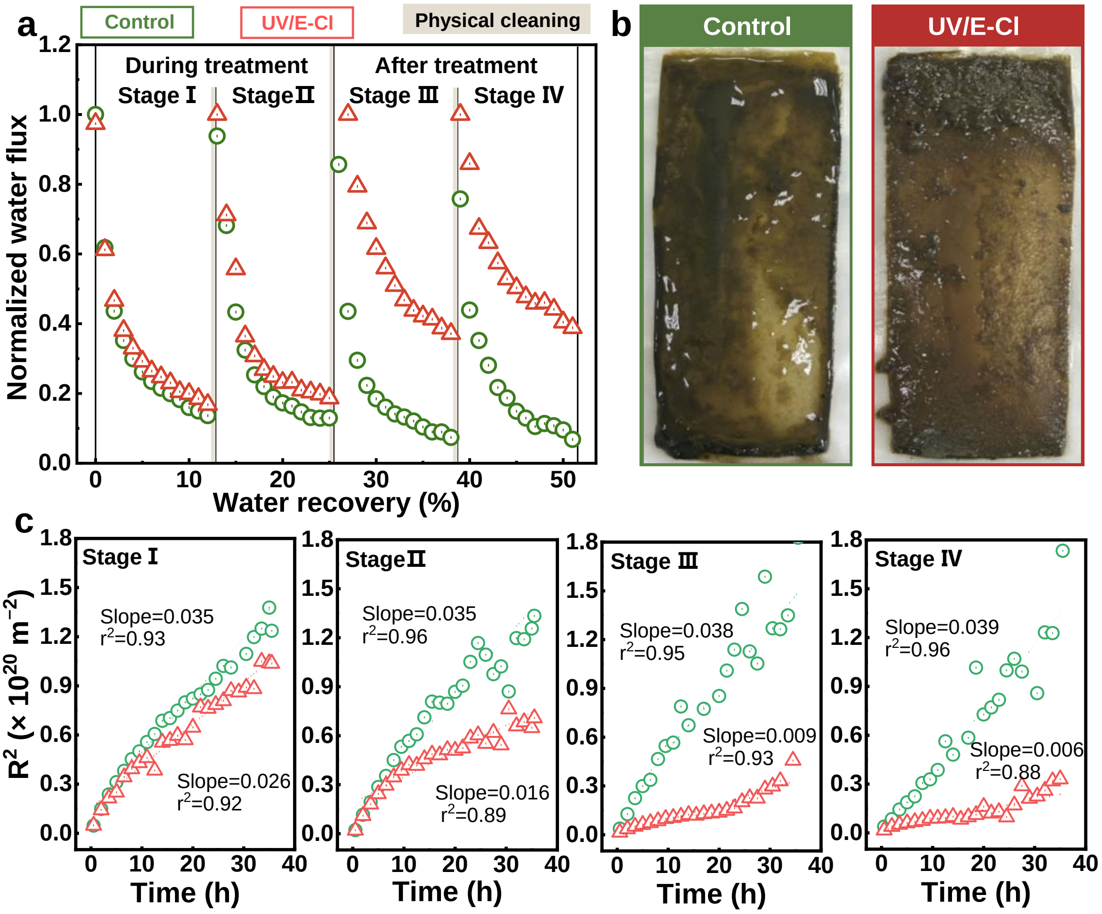

Supplementary Fig. 1. WAS dewatering performance assessment. a Normalized water flux with water recovery. **b** Direct observation of foulant layers. **c** Cake filtration model fitting for water flux, where *R* represents filtration resistance. Experimental conditions: draw solution = 1 M NaCl, current density = 7.84 mA·cm^{-2}, UV power = 10 W, UV fluence rate = 0.44 mW·cm^{-2}, and intermittent exposure mode = 5'-ON/25'-OFF.

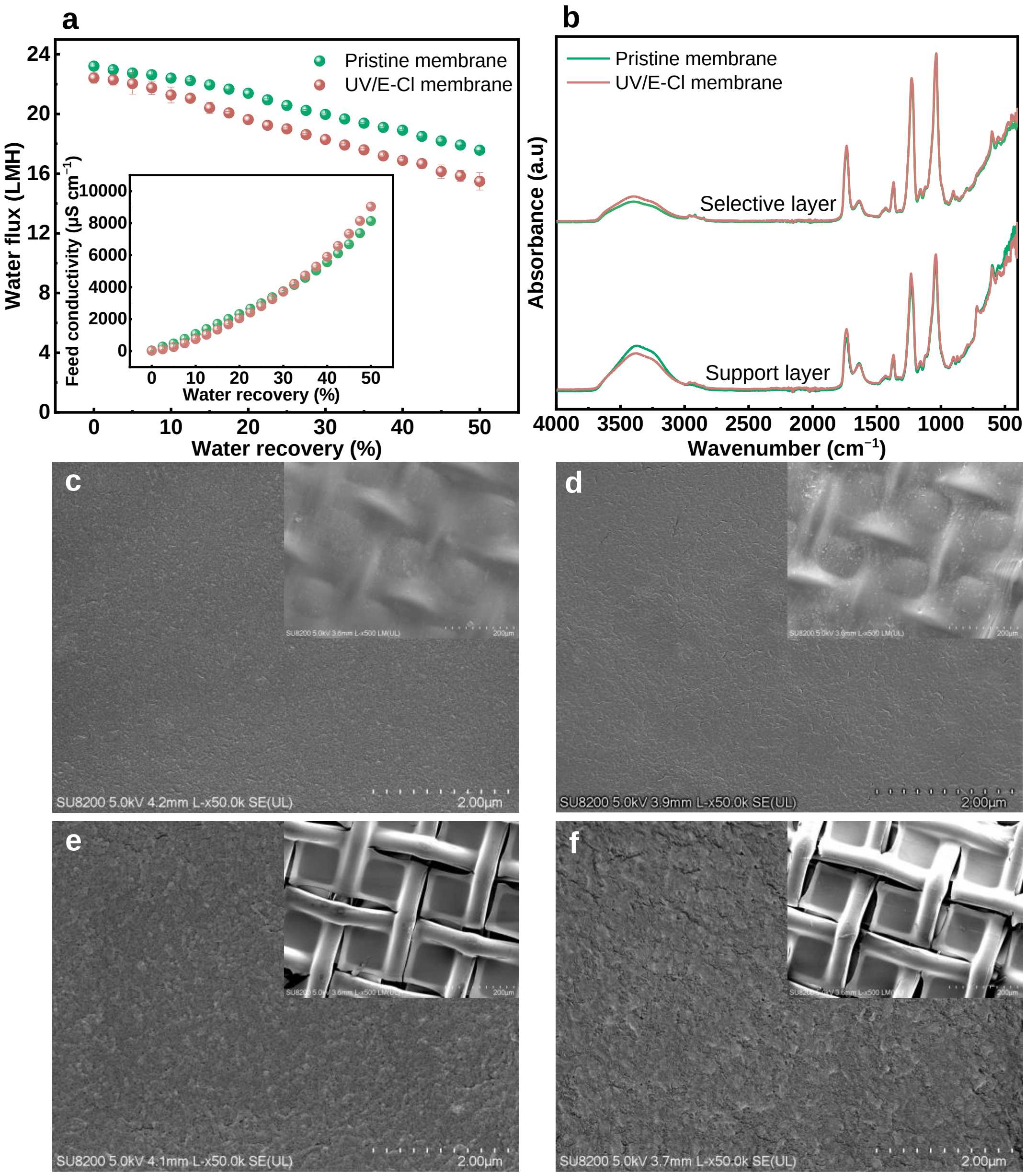

Supplementary Fig. 2. Characterization of membrane integrity after UV/E-Cl cycles. a Comparison of water flux and feed conductivity between the pristine membrane and the used membrane after 4-cycle UV/E-Cl processes using deionized water as feed solution and 4 M NaCl as draw solution. **b** FTIR absorbance spectra (4000-500 cm^{-1}) for the selective layer and the support layer of the pristine membrane and the used membrane. SEM images showing **c-d** the selective layer of a pristine membrane and the used membrane, respectively, and **e-f** the support layer of a pristine membrane and the used membrane, respectively.

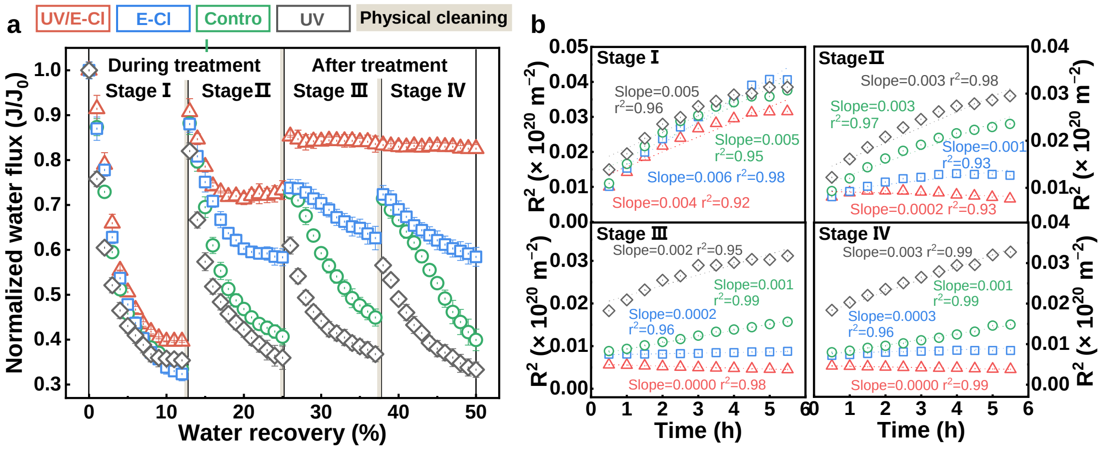

Supplementary Fig. 3. Dewatering performance validation using SA-BSA mixtures as model EPS. a Normalized water flux with water recovery. **b** Cake filtration model fitting for water flux, where *R* represents filtration resistance. Experimental conditions: feed solution = SA-BSA mixtures ($[SA]_0$ = $[BSA]_0$ = 500 mg·L^{-1}), draw solution = 4 M NaCl, current density = 7.84 mA·cm^{-2}, UV power = 10 W, UV fluence rate = 0.44 mW·cm^{-2}, and intermittent exposure mode = 5'-ON/25'-OFF.

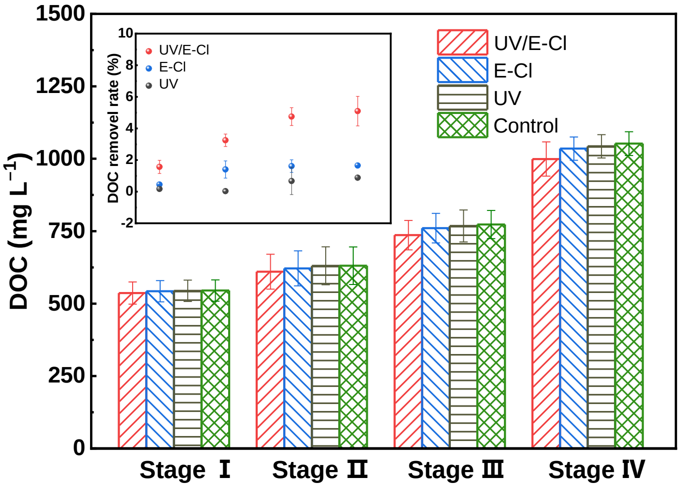

Supplementary Fig. 4. DOC removal performance for SA-BSA mixtures. Experimental conditions: feed solution = SA-BSA mixtures ($[SA]_0 = [BSA]_0 = 500$ $mg \cdot L^{-1}$), draw solution = 4 M NaCl, current density = 7.84 $mA \cdot cm^{-2}$, UV power = 10 W, UV fluence rate = 0.44 $mW \cdot cm^{-2}$, and intermittent exposure mode = 5’-ON/25’-OFF.

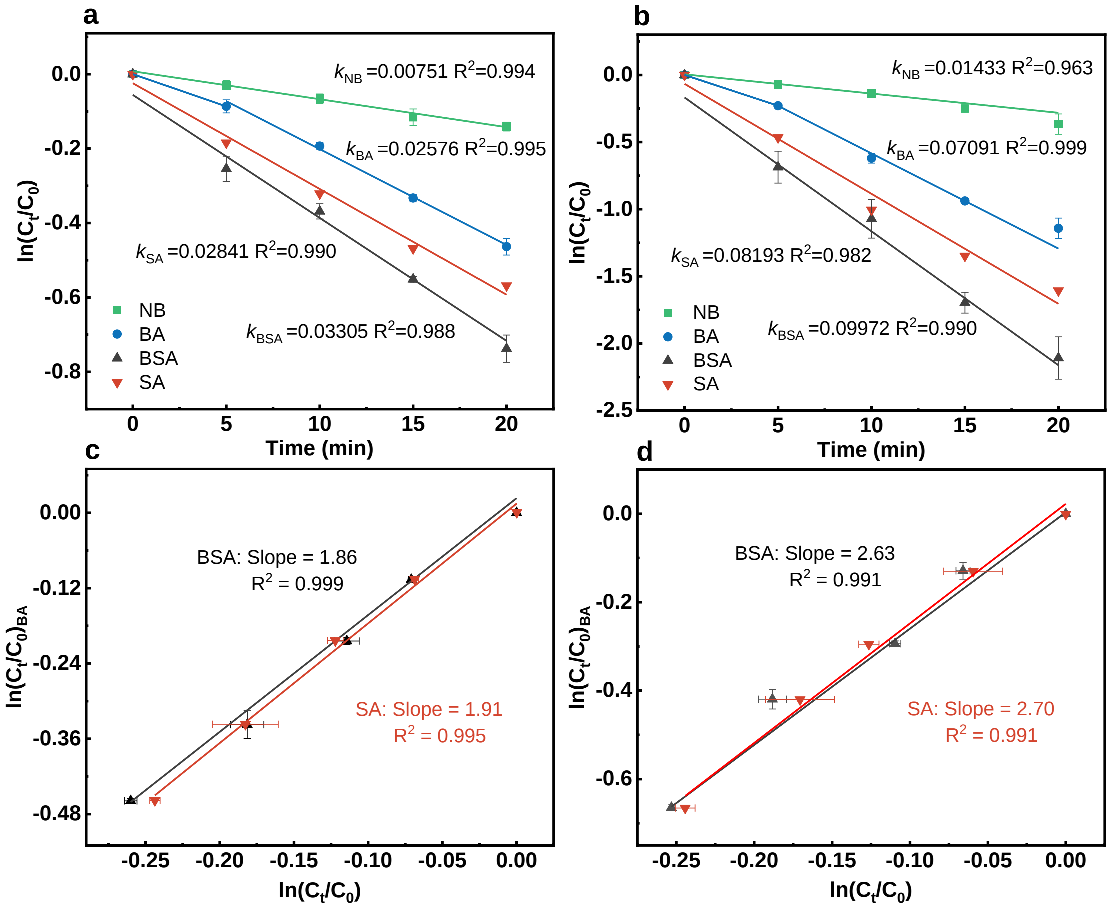

Supplementary Fig. 5. Identification of dominant reactive species during SA-BSA system. a Degradation kinetics of coexisting NB, BA, BSA and SA in the UV/E-Cl process refer to stage I. Conditions: 25 °C, $[HClO]_0$ = 0.30 mM, $[Cl^-]_0$ = 264 mg·L^{-1}, $[BSA]_0$ = $[SA]_0$ = 50 mg·L^{-1}, $[BA]_0$ = $[NB]_0$ = 0.01 mM, UV fluence rate = 0.44 mW·cm^{-2}. **b** Degradation kinetics of coexisting NB, BA, BSA and SA in the UV/E-Cl process refer to stage II. Conditions: 25 °C, $[HClO]_0$ = 0.60 mM $[Cl^-]_0$ = 823 mg·L^{-1}, $[BSA]_0$ = $[SA]_0$ = 50 mg·L^{-1}, $[BA]_0$ = $[NB]_0$ = 0.01 mM, UV fluence rate = 0.44 mW·cm^{-2}. **c** Competition kinetics between BA, BSA and SA in the UV/H_2O_2 system. Condition: 25 °C, $[H_2O_2]_0$ = 0.30 mM, $[Cl^-]_0$ = 264 mg·L^{-1}, $[BSA]_0$ = $[SA]_0$ = 50 mg·L^{-1}, $[BA]_0$ = $[NB]_0$ = 0.01 mM, UV fluence rate = 0.44 mW·cm^{-2}. **d** Competition kinetics between BA, BSA and SA in the UV/H_2O_2 system. Condition: 25 °C, $[H_2O_2]_0$ = 0.60 mM, $[Cl^-]_0$ = 823 mg·L^{-1}, $[BSA]_0$ = $[SA]_0$ = 50 mg·L^{-1}, $[BA]_0$ = $[NB]_0$ = 0.01 mM, UV fluence rate = 0.44 mW·cm^{-1}.

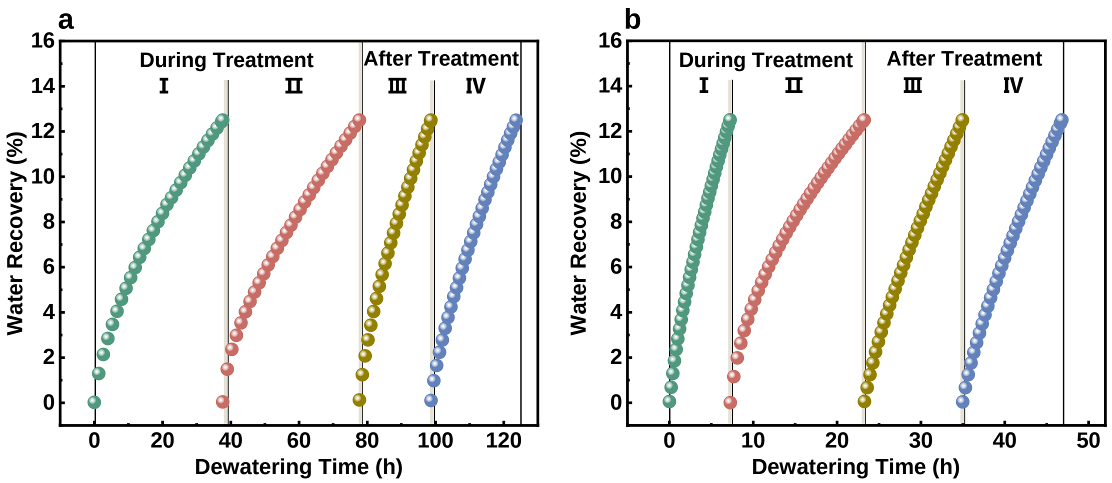

Supplementary Fig. 6. Comparison of WAS dewatering performance under different NaCl concentrations as draw solutions. Dewatering time required to achieve 50% water recovery using the UV/E-Cl process with **a** 1 M NaCl and **b** 4 M NaCl as draw solutions. Experimental conditions: current density = 7.84 mA·cm^{-2}, UV power = 10 W, UV fluence rate = 0.44 mW·cm^{-2}, and intermittent exposure mode = 5'-ON/25'-OFF.

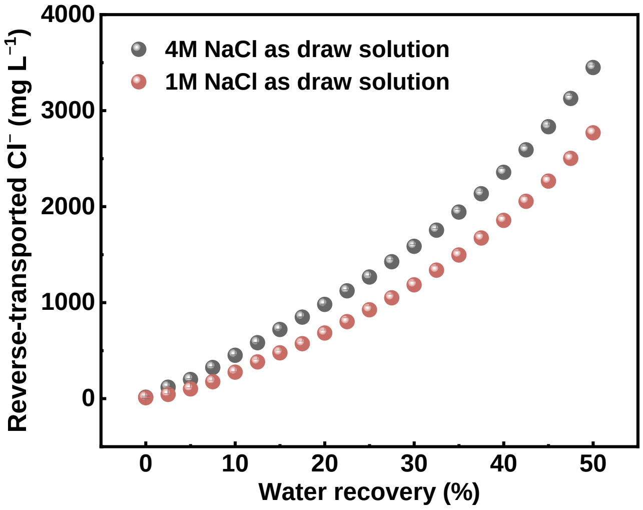

Supplementary Fig. 7. Reverse-transported Cl^- concentration during 1 M NaCl and 4 M NaCl as draw solution.

Supplementary Table 1. Key characteristics of WAS collected from the secondary sedimentation of Xiajiahe wastewater treatment plant in Dalian, China.

Mixed liquor suspended solids (MLSS) ($g \cdot L^{-1}$)	Conductivity ($mS \cdot cm^{-1}$)	pH
9.46±0.31	8.09±0.43	7.67±0.14

Supplementary Table 2. Competition kinetics results during BSA and SA degradation by UV/E-Cl using NB and BA as radical probes. Conditions: 25°C, $[BSA]_0 = [SA]_0 = 50\ mg\cdot L^{-1}$, $[BA]_0 = [NB]_0 = 0.01$ mM.

	Stage I	Stage II
k_{NB} (min^{-1})	0.00751	0.0143
k_{BA} (min^{-1})	0.0258	0.0710
k_{BSA} (min^{-1})	0.0331	0.100
k_{SA} (min^{-1})	0.0284	0.0819
$[HO\bullet]_{ss}$ (M)	3.21×10^{-14}	6.12×10^{-14}
$[Cl\bullet]_{ss}$ (M)	1.33×10^{-14}	4.56×10^{-14}
$k_{HO\bullet, BSA}$ (M^{-1} s^{-1})	3.17×10^{9}	2.24×10^{9}
$k_{Cl\bullet, BSA}$ (M^{-1} s^{-1})	3.37×10^{10}	3.34×10^{10}
$k_{HO\bullet, SA}$ (M^{-1} s^{-1})	3.09×10^{9}	2.19×10^{9}
$k_{Cl\bullet, SA}$ (M^{-1} s^{-1})	2.81×10^{10}	2.70×10^{10}
Cl• contribution on BSA degradation	91%	94%
HO• contribution on BSA degradation	9%	6%
Cl• contribution on SA degradation	90%	93%
HO• contribution on SA degradation	10%	7%

Supplementary Table 3. The contact angles and zeta potentials of membrane and SA-BSA foulants.

Material	Contact angle (°)			Zeta Potential (mV)
	Ultrapure water	Glycerol	Diiodomethane	
CTA active layer	69.39±2.78	71.16±1.43	43.26±2.90	-19.11±1.01
SA-BSA (control)	63.50±0.29	71.32±0.69	44.68±1.75	-30.89±2.85
SA-BSA (UV/E-Cl)	65.01±1.13	68.24±1.53	49.89±1.38	-39.63±1.95

Supplementary Table 4. Evaluation of the specific role of NaCl in the experiments.

$J_{4M\text{-control}}$ (LMH)	$J_{4M\text{-UVECl}}$ (LMH)	$J_{1M\text{-control}}$ (LMH)	$J_{1M\text{-UVECl}}$ (LMH)	Inherent contribution (%)	Cl• contribution (%)
0.65	4.74	0.37	2.12	10.69	89.31

$$\text{Inherent contribution }(\%) = \frac{J_{e\ (4M-control)} - J_{e\ (1M-control)}}{J_{e\ (4M-UVECl)} - J_{e\ (1M-UVECl)}} \tag{13}$$

$$\text{RCS contribution }(\%) = \frac{\left(J_{e\ (4M-UVECl)} - J_{e\ (4M-control)}\right) - \left(J_{e\ (1M-UVECl)} - J_{e\ (1M-control)}\right)}{J_{e\ (4M-UVECl)} - J_{e\ (1M-UVECl)}}$$

(14)where $J_{4M\text{-control}}$ is the water flux at the end of recovering 50% water content with no treatment and 4 M NaCl as draw solution. $J_{4M\text{-UVECl}}$ is the water flux at the end of recovering 50% water content with UV/E-Cl treatment and 4 M NaCl as draw solution. $J_{1M\text{-control}}$ is the water flux at the end of recovering 50% water content with no treatment and 1 M NaCl as draw solution. $J_{1M\text{-UVECl}}$ is the water flux at the end of recovering 50% water content with UV/E-Cl treatment and 1 M NaCl as draw solution.

Supplementary Table 5. Surface tension parameters of three probe liquids.

Probe liquids	γ^{LW}	γ^{+}	γ^{-}
Glycerol	34.0	3.9	57.4
Ultrapure water	21.8	25.5	25.5
Diiodomethane	50.8	0.0	0.0

Supplementary References

1. Rahn, R. O. Potassium iodide as a chemical actinometer for 254 nm radiation: Use of lodate as an electron scavenger. *Photochem. Photobiol.* **66**, 450-455 (1997).
2. Lv, L., Xu, J., Shan, B. & Gao, C. Concentration performance and cleaning strategy for controlling membrane fouling during forward osmosis concentration of actual oily wastewater. *J. Membr. Sci.* **523**, 15-23 (2017).
3. Aftab, B., Cho, J. & Hur, J. Intermittent osmotic relaxation: A strategy for organic fouling mitigation in a forward osmosis system treating landfill leachate. *Desalination* **482**, 114406 (2020).
4. Huisman, I. H., Dutré, B., Persson, K. M. & Trägårdh, G. Water permeability in ultrafiltration and microfiltration: Viscous and electroviscous effects. *Desalination* **113**, 95-103 (1997).
5. Ji, C.-C. et al. Fouling evolution of extracellular polymeric substances in forward osmosis based microalgae dewatering. *Water Res.* **229**, 119395 (2023).
6. Zhang, Y. et al. Integration of ultraviolet irradiation with electrochemical chlorine and hydrogen peroxide production for micropollutant abatement. *Chem. Eng. J.* **430**, 132804 (2022).
7. Hua, Z. et al. Exploring pathways and mechanisms for dichloroacetonitrile formation from typical amino compounds during UV/chlorine treatment. *Environ. Sci. Technol.* **56**, 9712-9721 (2022).
8. Bulman, D. M. & Remucal, C. K. Role of reactive halogen species in disinfection byproduct formation during chlorine photolysis. *Environ. Sci. Technol.* **54**, 9629-9639 (2020).
9. Allen, J. M. et al. Drivers of disinfection byproduct cytotoxicity in U.S. drinking water: Should other DBPs be considered for regulation? *Environ. Sci. Technol.* **56**, 392-402 (2022).
10. Fang, J., Fu, Y. & Shang, C. The roles of reactive species in micropollutant degradation in the UV/free chlorine system. *Environ. Sci. Technol.* **48**, 1859-1868 (2014).

11. Chen, L. et al. Peroxymonosulfate activated by composite ceramic membrane for the removal of pharmaceuticals and personal care products (PPCPs) mixture: Insights of catalytic and noncatalytic oxidation. *Water Res.* **229**, 119444 (2023).
12. Li, X. Y. & Yang, S. F. Influence of loosely bound extracellular polymeric substances (EPS) on the flocculation, sedimentation and dewaterability of activated sludge. *Water Res.* **41**, 1022-1030 (2007).
13. D'Abzac, P., Bordas, F., Van Hullebusch, E., Lens, P. N. L. & Guibaud, G. Extraction of extracellular polymeric substances (EPS) from anaerobic granular sludges: Comparison of chemical and physical extraction protocols. *Appl. Microbiol. Biot.* **85**, 1589-1599 (2010).
14. De Luca, G., Bisignano, F., Paone, F. & Curcio, S. Multi-scale modeling of protein fouling in ultrafiltration process. *J. Membr. Sci.* **452**, 400-414 (2014).
15. Zhang, M. et al. Mechanistic insights into alginate fouling caused by calcium ions based on terahertz time-domain spectra analyses and DFT calculations. *Water Res.* **129**, 337-346 (2018).
16. Guo, K. et al. Radical chemistry and structural relationships of PPCP degradation by UV/chlorine treatment in simulated drinking water. *Environ. Sci. Technol.* **51**, 10431-10439 (2017).
17. Huang, X. et al. Coupled Cu(II)-EDTA degradation and Cu(II) removal from acidic wastewater by ozonation: Performance, products and pathways. *Chem. Eng. J.* **299**, 23-29 (2016).
18. Huang, W. et al. The isolation, structural characterization and anti-osteosarcoma activity of a water soluble polysaccharide from Agrimonia pilosa. *Carbohyd. Polym.* **187**, 19-25 (2018).
19. Zhu, M. et al. Tyrosine residues initiated photopolymerization in living organisms. *Nat. Commun.* **14**, 3598 (2023).
20. Hu, G., Liu, X., Wang, Z., Du, X. & Wang, X. Comparison of fouling behaviors between activated sludge suspension in MBR and EPS model solutions: A new combined model. *J. Membr. Sci.* **621**, 119020 (2021).

21. Basuvaraj, M., Fein, J. & Liss, S. N. Protein and polysaccharide content of tightly and loosely bound extracellular polymeric substances and the development of a granular activated sludge floc. *Water Res.* **82**, 104-117 (2015).
22. Yi, X., Wang, Z., Zhao, P., Song, W. & Wang, X. New insights on destruction mechanisms of waste activated sludge during simultaneous thickening and digestion process via forward osmosis membrane. *Water Res.* **254**, 121378 (2024).
23. Nguyen, N. C., Chen, S.-S., Yang, H.-Y. & Hau, N. T. Application of forward osmosis on dewatering of high nutrient sludge. *Bioresour. Technol.* **132**, 224-229 (2013).
24. Hau, N. T. et al. Exploration of EDTA sodium salt as novel draw solution in forward osmosis process for dewatering of high nutrient sludge. *J. Membr. Sci.* **455**, 305-311 (2014).
25. Nguyen, N. C., Nguyen, H. T., Chen, S.-S., Nguyen, N. T. & Li, C.-W. Application of forward osmosis (FO) under ultrasonication on sludge thickening of waste activated sludge. *Water Sci. Technol.* **72**, 1301-1307 (2015).
26. Brant, J. A. & Childress, A. E. Assessing short-range membrane–colloid interactions using surface energetics. *J. Membr. Sci.* **203**, 257-273 (2002).
27. Bouchard, C. R., Jolicoeur, J., Kouadio, P. & Britten, M. Study of humic acid adsorption on nanofiltration membranes by contact angle measurements. *Can. J. Chem. Eng.* **75**, 339-345 (1997).
28. Lee, S., Kim, S., Cho, J. & Hoek, E. M. V. Natural organic matter fouling due to foulant–membrane physicochemical interactions. *Desalination* **202**, 377-384 (2007).
