# Supplementary material for: Dynamic reverse Cl− driven integration of sludge conditioning and dewatering
Source: Nat Commun. 2025 Mar 19;16:2717. doi: 10.1038/s41467-025-57878-4 (PMC11923106; doi:10.1038/s41467-025-57878-4)
Supplement: Supplementary file 2 — Transparent Peer Review file [file 41467_2025_57878_MOESM2_ESM.pdf]

Peer Review File

Dynamic reverse Cl⁻ driven integration of sludge conditioning and dewatering

Corresponding Author: Professor Hanmin Zhang

This file contains all reviewer reports in order by version, followed by all author rebuttals in order by version.

Version 0:

Reviewer comments:

Reviewer #1

(Remarks to the Author)
This manuscript integrated a novel system where dynamic reverse Cl drives in-situ UV/electrooxidation (UV/E-Cl) process in forward osmosis for simultaneous conditioning and dewatering of waste activated sludge (WAS). It was quantitatively calculated by filtration thermodynamics, which indicated that the shift of water occurrence states from bound to free was dominant in enhanced dewatering performance. However, sludge composition is far more complicated than SA-BSA. The latter cannot represent the former well. Please address the following comments.

1. The full name of SA and BSA should be provided in the manuscript.
2. There are a lot of formatting and grammar mistakes in the manuscript, which should be checked carefully. For example, (1) Please keep significant digits consistent “Except stage I where UV/E-Cl system appears R2stageI = 3.69 × 1018 similar to the blank with R2stageI = 4.3 × 1018 ……” (lines 142-146). (2) There are no a,b,c markers in Figure 7, but they appear in the main text (Lines 254-256). (3) The sentence at lines 55-61 is hard to follow, please rephrase.
3. Peptide bonds are one of the most common functional groups in the SA-BSA mixed solution. N-disinfection byproducts (N-DBPs) may be generated during UV/chlorine treatment (Environ. Sci. Technol. 2022, 56, 13, 9712–9721), which will increase the toxicity of the sludge. So, disinfection byproducts should be evaluated in UV/electrooxidation system.
4. A host of works have been reported and applied to the pretreatment of sludge dewatering (Water Res. 2020, 184, 116149; Water Res. 2023, 244, 120496). The UV/E-Cl system should be compared with the state-of-the-art to highlight its advantages.
5. The HO• and Cl• have been confirmed by EPR signal. Although HO• could be rapidly converted into RCS in an environment rich in free chlorine and Cl−, the relative contributions of HO• and Cl• to sludge dewatering should be evaluated
6. The authors stated in the manuscript that a homogeneous gel-like layer without actual connecting pores appears in the untreated condition (Fig. 8c1). Please provide additional test to demonstrate the results, such as, nitrogen adsorption-desorption isotherm.
7. Sludge composition is more complicated than model proteins and polysaccharides. The manuscript focuses on clarifying the correlation between the conditioned sludge properties and the FO membrane dewatering behaviors, however, the practical application of the UV/E-Cl system requires to be verified in actual sludge dewatering.

Reviewer #2

(Remarks to the Author)
In this study, the author attempted to elucidate the sludge conditioning and dewatering process by integrating chlorine-based AOP and the FO process. While the study itself may be of interest, my major concern is that the synthetic organic matter, such as the SA-BSA mixture used, may not accurately simulate actual waste activated sludge. Therefore, I recommend rejecting this manuscript and suggest that the paper should be rewritten and resubmitted with a focus on the organic fouling control of the FO system rather than the sludge dewatering process. Several issues and questions are raised in this manuscript, as outlined below:
1. L68-69. The author characterized the sludge by introducing organic matter, including polysaccharides and proteins. This suggests that the author's assumption is flawed, indicating a focus on investigating organic fouling in the FO system rather

than comprehensively addressing the broader aspects of sludge. Additionally, references (55 and 64) fail to establish a connection with the dewatering experiment (L343-345).
2. L345-346. Why was a higher concentration of NaCl (4 M) used as the draw solution in this experiment, considering that the NaCl concentration in natural seawater typically ranges from 0.5 M to 1.0 M? Additionally, why was a 1,000 mg/L SA-BSA mixture used for the model sludge concentration in these experiments?
3. L 125. The reverse salt (Cl) flux and free chlorine concentration are the most important factors in this experiment. Therefore, the author should provide reverse salt flux and free chlorine data for each experimental case (UV/E-Cl, E-Cl, control, UV).
4. L136. I confused the conditions with treatment and without treatment. How could higher performance be achieved without treatment in Figure 2a?
5. L159. The floc size decreased after the UV/E-Cl treatment. Generally, a smaller floc size could accelerate the formation of a more dense fouling layer compared to larger flocs. However, the experimental results contradict this observation. Why?
6. L254. I cannot find information about Figures 7a and 7b in the manuscript. Additionally, were the DFT simulations conducted by the authors, or were the results cited from other works? The author should provide a more detailed explanation about the DFT simulations.
7. L281-286. The explanation is quite abstract, and a more direct explanation should be provided based on the experimental results.
8. L292-335. The proposed mechanism illustrates changes in the formation of the organic fouling layer by the UV/E-Cl system, but it does not focus on the aspects of dewatering waste activated sludge. As I asked in Q1, the experimental concept is closer to the control of organic fouling in FO rather than the sludge dewatering system.

Version 1:

Reviewer comments:

Reviewer #1

(Remarks to the Author)
The authors have solved most of my comments and revised the manuscript. They proved the concept by using real wastewater sludge. However, there still some issues should be considered. The major aspect is the author should report the results of real sludge rather than SB-BSA in the main text.

1. The authors proved that UV/E-Cl could alleviate FO membrane fouling, and increased water flux. My major concern is whether the highly oxidative chlorine and radicals will destroy the membrane or reduce its effective time. The authors should characterize the membrane material after using several cycles.

2. The DBP formation from real wastewater sludge should be evaluated, as this case is totally different from the SA-BSA case.

3. The authors used EPR and NB to prove the effects radicals. However, EPR cannot confirm the signal of Cl*. When high concentrations of NB was used to scavenge HO*, NB was filter most UV light simutaneously. Thus, spiking NB cannot prove the roles of either HO* or Cl*.

4. The English written should be further improved.

Reviewer #2

(Remarks to the Author)
The author successfully incorporated the revisions suggested by the reviewers into the manuscript. However, one question still remains regarding the revised manuscript as follows:
- This question pertains to Q2 from the first-round review. The concentration of the draw solution is a critical operating parameter in the FO process. The author provided data using 4 M NaCl as the draw solution. However, the actual sludge dewatering experiment used 1 M NaCl as the draw solution. The difference in draw solution concentration can significantly alter the performance of the FO process, affecting parameters such as water flux, reverse salt flux, and fouling formation. Therefore, these supplementary experimental results do not correspond to the conditions under which 4 M NaCl was used.

Version 2:

Reviewer comments:

Reviewer #1

(Remarks to the Author)
The authors have well addressed most of my comments and improved the manuscript. To enhance the robustness and completeness of the article, further optimization of experimental parameters, improvement of data clarity, and additional theoretical support and future outlooks are necessary. The detailed reviews are as follows:

1. The article provides a comprehensive background on sludge dewatering, including a thorough analysis of the potential and existing challenges associated with FO technology. It is recommended to include a detailed comparison of current mainstream dewatering technologies to highlight the innovations of this study. Specifically, an analysis of the advantages and disadvantages of other membrane fouling mitigation techniques would better illustrate the unique benefits of the dynamic reverse chloride-driven UV/E-Cl process.

2. The analysis of experimental results is thorough, particularly with the innovative application of thermodynamic theories to explain membrane fouling mechanisms. However, it is advisable to include an uncertainty analysis of the experimental results, addressing factors that might affect reproducibility, such as variations in initial sludge composition and membrane aging.

3. NaCl is used as the draw solution in the experiments, but it is important to note that high-concentration sodium chloride solutions possess inherent dehydration capabilities. Please clarify how to differentiate the specific role of NaCl in the experiments—whether the RCS acts as the primary driver of dehydration or if sodium chloride itself contributes to the dewatering of the sludge. Additionally, further exploration of the impact of varying NaCl concentrations on system performance would help assess the applicability and limitations of the process.

4. Please elaborate on the advantages of using AOP for sludge dewatering. Most advanced oxidation methods disrupt the physicochemical structure of EPS, typically targeting peptide bonds in proteins and glycosidic bonds in polysaccharides. The experiments demonstrate that these technologies generate free radicals that attack EPS, but they do not fully illustrate their comparative superiority.

5. The conclusion effectively summarizes the main research findings but provides a rather brief outlook on future research. It is suggested that the authors elaborate on the practical application potential of the process in real-world sludge treatment and propose specific research directions, such as further optimization of UV/E-Cl process parameters, exploring synergistic effects with other oxidants, or assessing the technology’s applicability to different types of sludge.

Reviewer #2

(Remarks to the Author)
1. L68-69. The author characterized the sludge by introducing organic matter, including polysaccharides and proteins. This suggests that the author's assumption is flawed, indicating a focus on investigating organic fouling in the FO system rather than comprehensively addressing the broader aspects of sludge. Additionally, references (55 and 64) fail to establish a connection with the dewatering experiment (L343-345).
2. L345-346. Why was a higher concentration of NaCl (4 M) used as the draw solution in this experiment, considering that the NaCl concentration in natural seawater typically ranges from 0.5 M to 1.0 M? Additionally, why was a 1,000 mg/L SA-BSA mixture used for the model sludge concentration in these experiments?
3. L 125. The reverse salt (Cl) flux and free chlorine concentration are the most important factors in this experiment. Therefore, the author should provide reverse salt flux and free chlorine data for each experimental case (UV/E-Cl, E-Cl, control, UV).
4. L136. I confused the conditions with treatment and without treatment. How could higher performance be achieved without treatment in Figure 2a?
5. L159. The floc size decreased after the UV/E-Cl treatment. Generally, a smaller floc size could accelerate the formation of a more dense fouling layer compared to larger flocs. However, the experimental results contradict this observation. Why?
6. L254. I cannot find information about Figures 7a and 7b in the manuscript. Additionally, were the DFT simulations conducted by the authors, or were the results cited from other works? The author should provide a more detailed explanation about the DFT simulations.
7. L281-286. The explanation is quite abstract, and a more direct explanation should be provided based on the experimental results.
8. L292-335. The proposed mechanism illustrates changes in the formation of the organic fouling layer by the UV/E-Cl system, but it does not focus on the aspects of dewatering waste activated sludge. As I asked in Q1, the experimental concept is closer to the control of organic fouling in FO rather than the sludge dewatering system.

Version 3:

Reviewer comments:

Reviewer #1

(Remarks to the Author)
The authors have responded most comments. However, I find some of important concerns was not accrodingly revised in the manuscript. The major issues are:

1. The formation of chlorinated byproducts should be mentioned in the manuscript, as people always concern the toxic chlorinated byproducts formed during E/UV-Cl.

2. Even though the authors hold the opinion that SA-BSA can stand for actual sludge. The property of SA-BSA is totally different from autual sludge. For example, the former is dissoved, the latter is biological particles. The results of actual

sludge should be provided in the main text.

Version 4:

Reviewer comments:

Reviewer #1

(Remarks to the Author)
Accept as is.

Open Access This Peer Review File is licensed under a Creative Commons Attribution 4.0 International License, which permits use, sharing, adaptation, distribution and reproduction in any medium or format, as long as you give appropriate credit to the original author(s) and the source, provide a link to the Creative Commons license, and indicate if changes were made.
In cases where reviewers are anonymous, credit should be given to 'Anonymous Referee' and the source.
The images or other third party material in this Peer Review File are included in the article’s Creative Commons license, unless indicated otherwise in a credit line to the material. If material is not included in the article’s Creative Commons license and your intended use is not permitted by statutory regulation or exceeds the permitted use, you will need to obtain permission directly from the copyright holder.
To view a copy of this license, visit https://creativecommons.org/licenses/by/4.0/

Responses to Comments and Suggestions of Reviewers

Re manuscript: NCOMMS-23-55103.

Title: Dynamic reverse Cl^{-} driven integration of sludge conditioning and dewatering: Emphasis on water occurrence states.

ANSWER: The authors would like to thank the editor and the reviewers for their very positive feedback as well as critical and constructive comments on the quality and novelty of the fouling control strategy and the approach proposed in our work. The manuscript has been significantly modified to address the comments, suggestions and recommendations for improvement provided by the editor and reviewers. Changes in the revised manuscript and supplementary information are highlighted in red. The following are our point-to-point responses to all the reviewers' comments.

Responses to Reviewer #1 's comments:

General comments:

This manuscript integrated a novel system where dynamic reverse Cl drives in-situ UV/electrooxidation (UV/E-Cl) process in forward osmosis for simultaneous conditioning and dewatering of waste activated sludge (WAS). It was quantitatively calculated by filtration thermodynamics, which indicated that the shift of water occurrence states from bound to free was dominant in enhanced dewatering performance. However, sludge composition is far more complicated than SA-BSA. The latter cannot represent the former well. Please address the following comments.

ANSWER: The authors are very grateful to the reviewer for giving valuable comments and suggestions. We also fully understand your concerns and have supplemented the practical application of the UV/E-Cl assisted FO system on actual sludge dewatering to validate the core claims of our work. The manuscript has been significantly modified according to your following issues and suggestions.

Specific comments:

1. The full name of SA and BSA should be provided in the manuscript.

ANSWER: Thanks for pointing this out. The full name of SA (sodium alginate) and

BSA (bovine serum albumin) has been provided in the manuscript. (Please see lines 67-70 in the revised manuscript).

2. There are a lot of formatting and grammar mistakes in the manuscript, which should be checked carefully. For example, (1) Please keep significant digits consistent "Except stage I where UV/E-Cl system appears R2stageI = 3.69 × 1018 similar to the blank with R2stageI = 4.3 × 1018 ……" (lines 142-146). (2) There are no a,b,c markers in Figure 7, but they appear in the main text (Lines 254-256). (3) The sentence at lines 55-61 is hard to follow, please rephrase.

ANSWER: Thank you for your reminders. We have corrected the mistakes (1-3) you pointed out.

For (1), we have kept significant digits consistent as 3 (Please see lines 140-143 in the revised manuscript);

For (2), we have added a, b, c markers (Please see Fig. 6 in the revised manuscript);

For (3), "Typically, undesirable salinity accumulation occurs on the feed side (FS) due to inevitable solute reverse diffusion from the draw side (DS). Accordingly, it may be feasible to construct a green and flexible integrated module, i.e., reverse Cl^{-} driven in-situ UV/E-Cl (electrochemical oxidation of Cl^{-} combined with UV irradiation) assisted FO process. This scenario should achieve a "mutually-beneficial" goal in terms of salinity consumption, membrane fouling mitigation and simultaneous sludge dewatering enhancement." (please see lines 56-62 in the revised manuscript).

We re-checked the whole manuscript carefully and corrected the mistakes involved.

3. Peptide bonds are one of the most common functional groups in the SA-BSA mixed solution. N-disinfection byproducts (N-DBPs) may be generated during UV/chlorine treatment (Environ. Sci. Technol. 2022, 56, 13, 9712–9721), which will increase the toxicity of the sludge. So, disinfection byproducts should be evaluated in UV/electrooxidation system.

ANSWER: Thank you for your constructive comment. We totally understand your concern. We have complemented an analysis of DBPs, including nitrogenous DBPs

(N-DBPs) and carbonaceous DBPs (C-DBPs), in both feed and draw side during actual WAS dewatering with UV/chlorine treatment (Fig. 1). For N-DBPs, we targeted four haloacetonitriles (HANs, i.e., bromochloroacetonitrile (BCAN), dibromoacetonitrile (DBAN), dichloracetonitrile (DCAN), and trichloroacetonitrile (TCAN))[1,2]. For C-DBPs, four regulated trihalomethanes (THMs, i.e., chloroform (TCM), bromodichloromethane (BDCM), chlorodibromomethane (DBCM) and bromoform (TBM)) and five regulated haloacetic acids (HAAs, i.e., monochloroacetic acid (CAA), dichloroacetic acid (DCAA), trichloroacetic acid (TCAA), monobromoacetic acid (BAA), and dibromoacetic acid (DBAA)) were focused on[3]. In feed solution, the sum of HANs was 40.7 $\mu g \cdot L^{-1}$, below the World Health Organisation (WHO) guideline value for drinking water of about 90 $\mu g \cdot L^{-1}$[4]. The sum of THMs was 55.1 $\mu g \cdot L^{-1}$, far below WHO guideline value for drinking water of 560 $\mu g \cdot L^{-1}$[4]. Although the sum of HAAs was 528.2 $\mu g \cdot L^{-1}$, higher than the guideline value for drinking water of 270 $\mu g \cdot L^{-1}$[5], there was no need to overly concern about the environmental risks because subsequent anaerobic digestion could effectively remove 68.68% DBPs[6]. As expected, DBPs migration into draw side was practically insignificant due to the excellent rejection of FO membrane[7-9]. Figure and related analytical information have been supplemented (Please see lines 339-341 in the revised manuscript and Fig. S6 in the revised supplementary information).

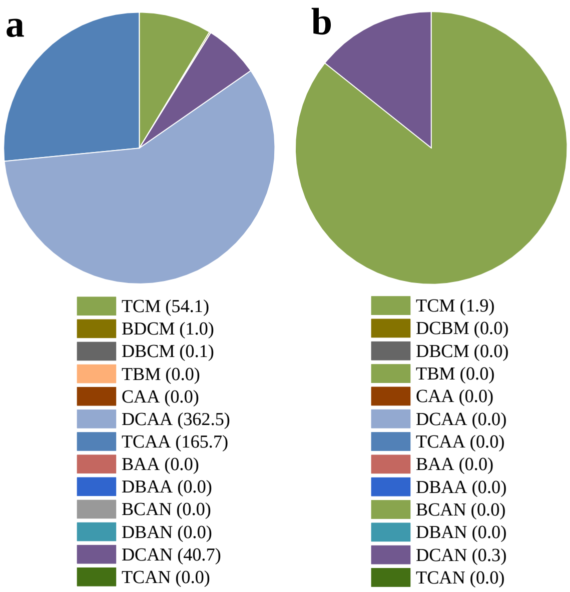

Fig. 1 DBPs analysis of **a** feed solution and **b** draw solution with UV/E-Cl treatment.

4. A host of works have been reported and applied to the pretreatment of sludge dewatering (Water Res. 2020, 184, 116149; Water Res. 2023, 244, 120496). The UV/E-Cl system should be compared with the state-of-the-art to highlight its advantages.

ANSWER: Thank you for your reminder. The two references have been supplemented and compared to highlight the advantages of the UV/E-Cl system proposed in our work. The sludge pretreatment strategy proposed in "Water Res. 2020, 184, 116149" involves hydroxyl radicals, which are non-selectively oxidized and susceptibly quenched. In contrast, reactive chlorine species generated during UV/E-Cl system can selectively attack polysaccharides and proteins, which are key components in the formation of gel-like membrane fouling and in limiting dewatering performance, at a rather high rate (~10^{10} M^{-1} s^{-1})[10]. (Please see lines 44-48 in the revised manuscript). As for "Water Res. 2023, 244, 120496", the sludge conditioning strategy is novelty. However, our focus is on the construction of a compact integrated sludge conditioning and dewatering system. At the technical level, we propose a novel in-situ UV/E-Cl system driven by unavoidable reverse-diffusion Cl^{-} during FO process without additional chemical inputs. At the theoretical level, our work provides the first quantitative thermodynamic confirmation of the dominant role of water occurrence state in mitigating membrane gel fouling and improving sludge dewatering performance, providing universal theoretical clues for the development of effective gel fouling mitigation and sludge dewatering enhancement strategies.

5. The HO• and Cl• have been confirmed by EPR signal. Although HO• could be rapidly converted into RCS in an environment rich in free chlorine and Cl^{-}, the relative contributions of HO• and Cl• to sludge dewatering should be evaluated.

ANSWER: Thank you for your thoughtful feedback. We have conducted quenching experiments by adding nitrobenzene (NB) radical scavenger reacting only with HO• for distinguishing the relative contributions of the HO• and Cl• to dewatering performance (Fig. 2). Water flux and filtration resistance remained almost unchanged with or without NB addition, confirming the dominant role of Cl•. (Please see Fig. S1

in the revised supplementary information and lines 147-151 in the revised manuscript).

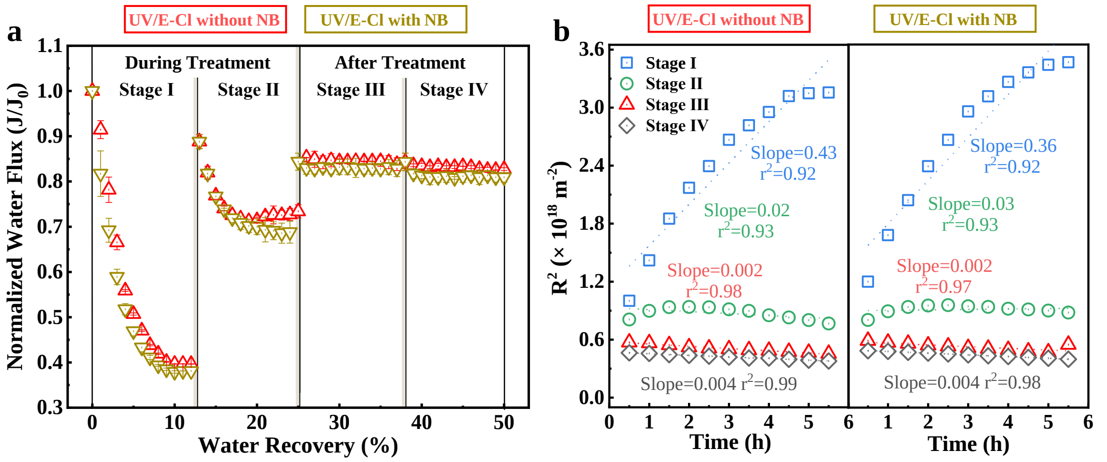

Fig. 2 a normalized water flux and **b** cake filtration model fit curves during SA-BSA mixtures dewatering with or without NB addition.

6. The authors stated in the manuscript that a homogeneous gel-like layer without actual connecting pores appears in the untreated condition (Fig. 8c1). Please provide additional test to demonstrate the results, such as, nitrogen adsorption-desorption isotherm.

ANSWER: Thank you for pointing this out. We realize that there is an expression error in the sentence "a homogeneous gel-like layer without actual connecting pores appears in the untreated condition (Fig. 8c$_1$)". It is not that there are without actual pores, but that the pores are occupied by bound water, resulting in an occlusion state, so called "without actual connecting pores". This sentence has been corrected as below: "Therefore, in the control, a homogeneous gel-like layer formed (Fig. 6a$_1$), with the pores occupied by abundant bound water (Fig. 7c$_1$)." (Please see lines 277-279 in the revised manuscript).

7. Sludge composition is more complicated than model proteins and polysaccharides. The manuscript focuses on clarifying the correlation between the conditioned sludge properties and the FO membrane dewatering behaviors, however, the practical application of the UV/E-Cl system requires to be verified in actual sludge dewatering.

ANSWER: Thank you for your constructive comment. Actual sludge dewatering tests

have been complemented to validate the core claims of our work as follows:

“**Actual WAS dewatering**. Actual WAS dewatering experiments verified that UV/E-Cl assisted FO process effectively improved dewatering flux for WAS compared with the control, as evidenced by an average 434% increase in stages III and IV (Fig. 3a). As expected, the foulant layer formed during WAS dewatering changed from a gel-like, to a cake-like state (Fig. 3b), in which water content decreased to 37.16% and bound water content dropped by 81.70% (from 37.16 to 6.80%) (Fig. 3c). Correspondingly, filtration resistance decreased by an order of magnitude to $R^2_{stageIII} = 4.57 \times 10^{19}$ and $R^2_{stageIV} = 3.31 \times 10^{19}$ (Fig. 3d), with low resistance rates of 0.009 and 0.006, respectively. UV/E-Cl was focused on destructing EPS physicochemical properties, which was initially confirmed by decreased size (Fig. 4a), apparent viscosity and zeta potential (Fig. 4b). Furthermore, EEM spectra reflected that polysaccharide and protein-like substances in EPS were targeted for degradation, as confirmed by a decrease in characteristic fluorescence intensities (Fig. 5a). FTIR spectra suggested that glycosidic and peptide bonds were the main sites attacked in polysaccharides and proteins, respectively (Fig. 5b), which led to a strong exposure of hydrophobic sites or groups inside proteins, corresponding to a reduced surface polarity (Fig. 6).” (Please see lines 323-338 in the revised manuscript).

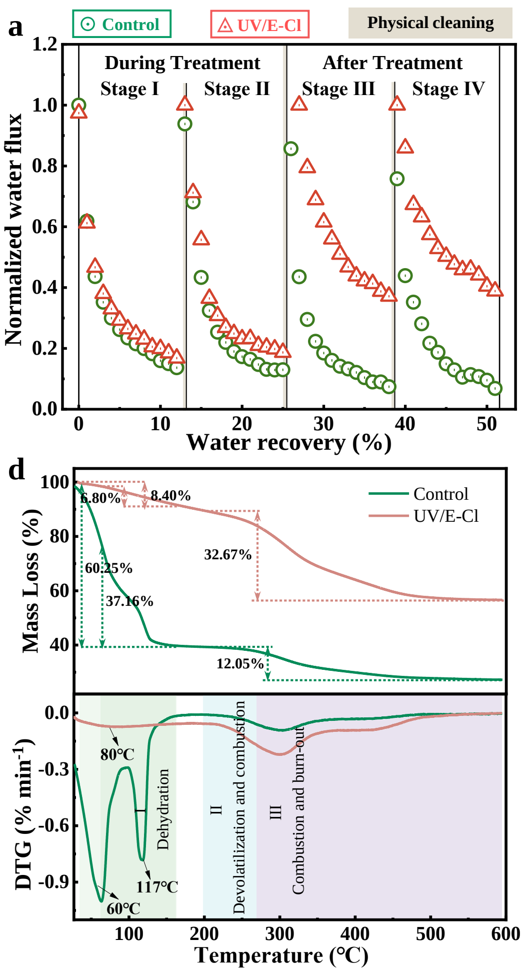

Fig. 3 Dewatering performance of UV/E-Cl assisted FO process for actual WAS: **a** normalized water flux; **b** direct observation of foulant layers; **c** thermal stabilities of foulant layers; and **d** filtration resistance. (Please see Fig. 9 in the revised manuscript).

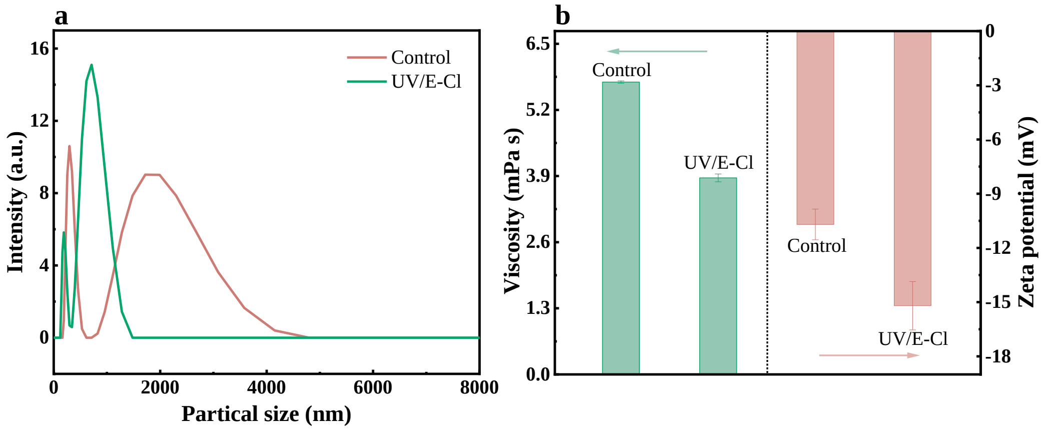

Fig. 4 UV/E-Cl effects on **a** particle size distribution and **b** apparent viscosity and zeta potential of EPS.

EPS size was significantly reduced from 1019 to 562 nm after UV/E-Cl

treatment (Fig. 4a). The apparent viscosity was reduced from 5.74 to 3.86 mPa·s, and the zeta potential of EPS solution became more negative value from −10.68 to −15.23 mV (Fig. 4b). These results indicated that UV/E-Cl treatment was efficient in breaking down the entangled network of the EPS matrix, in which the organic complexes formed by the biopolymers bridging each other were readily decomposed[11]. (Please see Fig. S3 in the revised supplementary information).

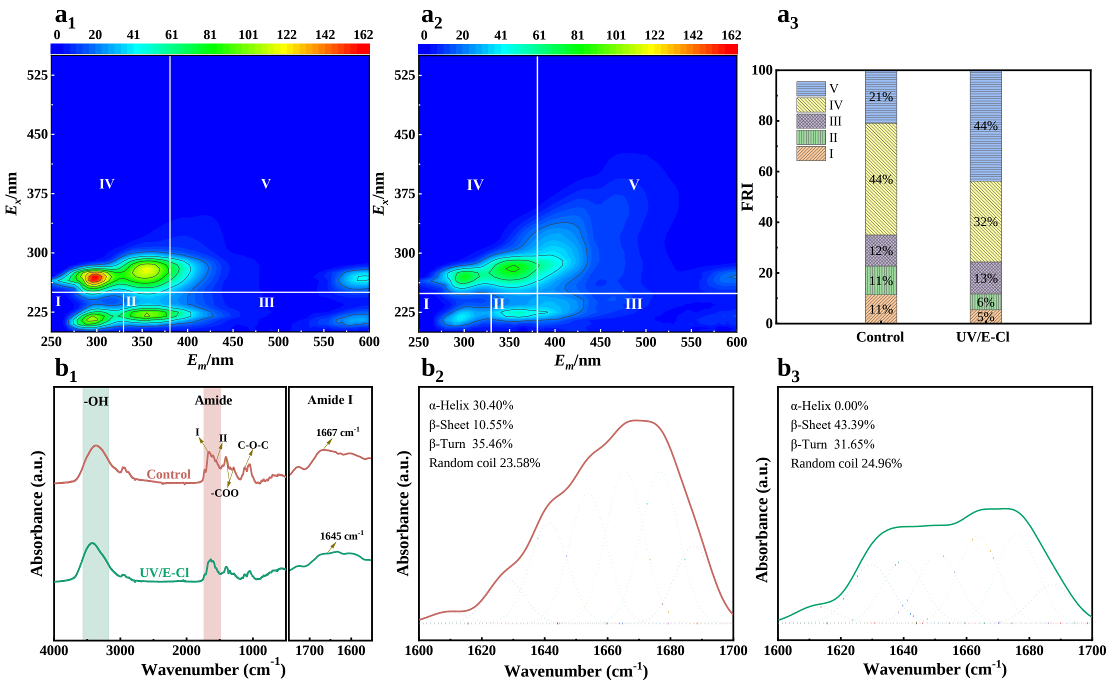

Fig. 5 **a** EEM spectra (a_1 refers to the control, a_2 refers to the UV/E-Cl and a_3 refers to FRI distribution); and **b** FTIR analysis (b_1 refers to FTIR spectra in the control and UV/E-Cl, b_2 refers to amide I fitting curves in the control and b_3 refers to amide I fitting curves in the UV/E-Cl) of EPS.

EEM spectra of EPS solution were analyzed using fluorescence regional integration (FRI) method by being divided into five excitation–emission regions (Fig. 5a_1 and a_2)[12]. Generally, regions I and II (E_x<250 nm, E_m<350 nm) are associated with simple aromatic proteins such as tyrosine and tryptophan[13]. Region III (200 nm<E_x<250 nm, E_m>380 nm) represents fulvic acid-like substances[14]. Region IV (250 nm<E_x<280 nm, E_m<380 nm) is related to soluble microbial by-product-like material (including microbial polysaccharides)[15-17]. Region V (E_x>280 nm, E_m>380 nm) is related to humic acid-like organics[14,18]. Regions I, II and III in UV/E-Cl process were

smaller than in the control (Fig. 5a3), implying that proteins and polysaccharide-like substances in EPS were targeted to be degraded.

FTIR spectra were further analyzed to acquire more characteristic information on proteins and polysaccharides in EPS (Fig. 5b1). With UV/E-Cl treatment, the intensity of amide I band decreased with blueshift from 1667 to 1645 cm^{-1} (see enlargement of amide I for details), and amide II band disappeared. Additionally, the intensity of C-O-C band significantly decreased. These results reconfirmed that UV/E-Cl effectively disrupted proteins and polysaccharides by attacking glycosidic and peptide bonds mainly, respectively, during the actual WAS dewatering process. Moreover, protein secondary structure showed that the protein helix structure was completely unfolded, as evidenced by the disappearance of α-helix and the decrease in α-helix/ (β-sheet + random coil) value from 89.07 to 0.00% (Fig. 5b2 and b3). Accordingly, the hydrophobic sites or groups inside proteins were easily exposed[19]. (Please see Fig. S4 in the revised supplementary information).

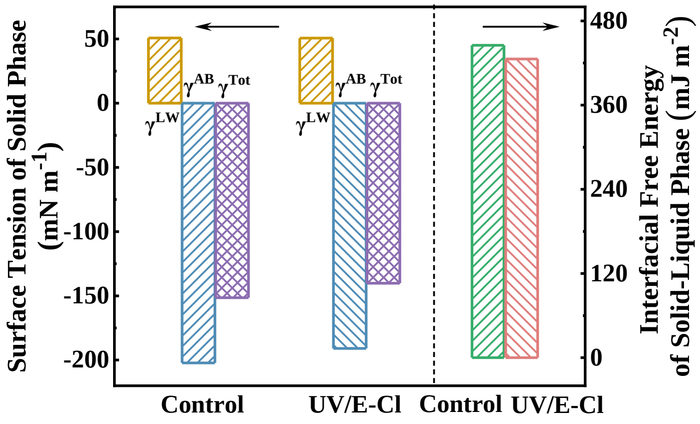

Fig. 6 EPS surface tension and interfacial free energy with water-phase.

With UV/E-Cl process, polar item (γ^{AB}) absolute value decreased from 202.24 to 190.93 $mN{\cdot}m^{-1}$, and solid-liquid phase interfacial free energy decreased from 445.29 to 425.69 $mJ{\cdot}m^{-2}$. (Please see Fig. S5 in the revised supplementary information).

Responses to Reviewer #2 's comments:

General Comments：

In this study, the author attempted to elucidate the sludge conditioning and dewatering process by integrating chlorine-based AOP and the FO process. While the study itself may be of interest, my major concern is that the synthetic organic matter, such as the SA-BSA mixture used, may not accurately simulate actual waste activated sludge. Therefore, I recommend rejecting this manuscript and suggest that the paper should be rewritten and resubmitted with a focus on the organic fouling control of the FO system rather than the sludge dewatering process. Several issues and questions are raised in this manuscript, as outlined below:

ANSWER: The authors sincerely thank the reviewer for giving approval and valuable comments for our work. The manuscript has been significantly modified according to your following issues and suggestions. We fully understand your concerns and after thorough consideration of your suggestions, we have decided to focus the manuscript on controlling organic membrane fouling during FO sludge dewatering process. And we have supplemented the practical application of the UV/E-Cl assisted FO system on actual sludge dewatering to verify the core claims of our work. Although our work was carried out with WAS, it is important to emphasize that the proposed and quantified water occurrence states mechanism is of universal significance for membrane gel fouling mitigation and membrane process operation.

Specific comments:

1. L68-69. The author characterized the sludge by introducing organic matter, including polysaccharides and proteins. This suggests that the author's assumption is flawed, indicating a focus on investigating organic fouling in the FO system rather than comprehensively addressing the broader aspects of sludge.

ANSWER: Thank you for your constructive feedback on our work. WAS dewatering/thickening is a key technical problem to be solved in the environmental field. Due to the complexity of WAS components, it is difficult to apply tools such as quantum chemistry simulations and thermodynamic analysis to gain insights into the

universal sludge dewatering mechanisms and thus provide theoretical clues for the development of WAS dewatering technologies. Extracellular polymers (EPS) in WAS have been identified as the key components limiting WAS dewatering[20-22]. Especially, proteins and polysaccharides in EPS have high molecular weights, abundant hydrophilic sites and a strong ability to trap water molecules[20,22]. Accordingly, we adopted proteins and polysaccharides as the model substances. We have reflected on the manuscript and realized that we might prefer to focus on organic membrane fouling during WAS dewatering, and in fact our manuscript confirmed the dominant role of organic fouling behaviors on WAS dewatering. We have refined the manuscript and clarified that the experimental concept of our work is to investigate the performance and underlying mechanism of novel in-situ UV/E-Cl in controlling organic membrane fouling during WAS dewatering. Subsequently, we conducted actual sludge dewatering experiments to validate the core claims of our work in improving WAS dewatering performance as follows:

"**Actual WAS dewatering**. Actual WAS dewatering experiments verified that UV/E-Cl assisted FO process effectively improved dewatering flux for WAS compared with the control, as evidenced by an average 434% increase in stages III and IV (Fig. 1a). As expected, the foulant layer formed during WAS dewatering changed from a gel-like, to a cake-like state (Fig. 1b), in which water content decreased to 37.16% and bound water content dropped by 81.70% (from 37.16 to 6.80%) (Fig. 1c). Correspondingly, filtration resistance decreased by an order of magnitude to $R^2_{stageIII}$ = 4.57 × 10^{19} and $R^2_{stageIV}$ = 3.31 × 10^{19} (Fig. 1d), with low resistance rates of 0.009 and 0.006, respectively. UV/E-Cl was focused on destructing EPS physicochemical properties, which was initially confirmed by decreased size (Fig. 2a), apparent viscosity and zeta potential (Fig. 2b). Furthermore, EEM spectra reflected that polysaccharides and protein-like substances in EPS were targeted for degradation, as confirmed by a decrease in characteristic fluorescence intensities (Fig. 3a). FTIR spectra suggested that glycosidic and peptide bonds were the main sites attacked in polysaccharides and proteins, respectively (Fig. 3b), which led to a strong exposure of hydrophobic sites or groups inside proteins, corresponding to a reduced surface

polarity (Fig. 4).” (Please see lines 323-338 in the revised manuscript).

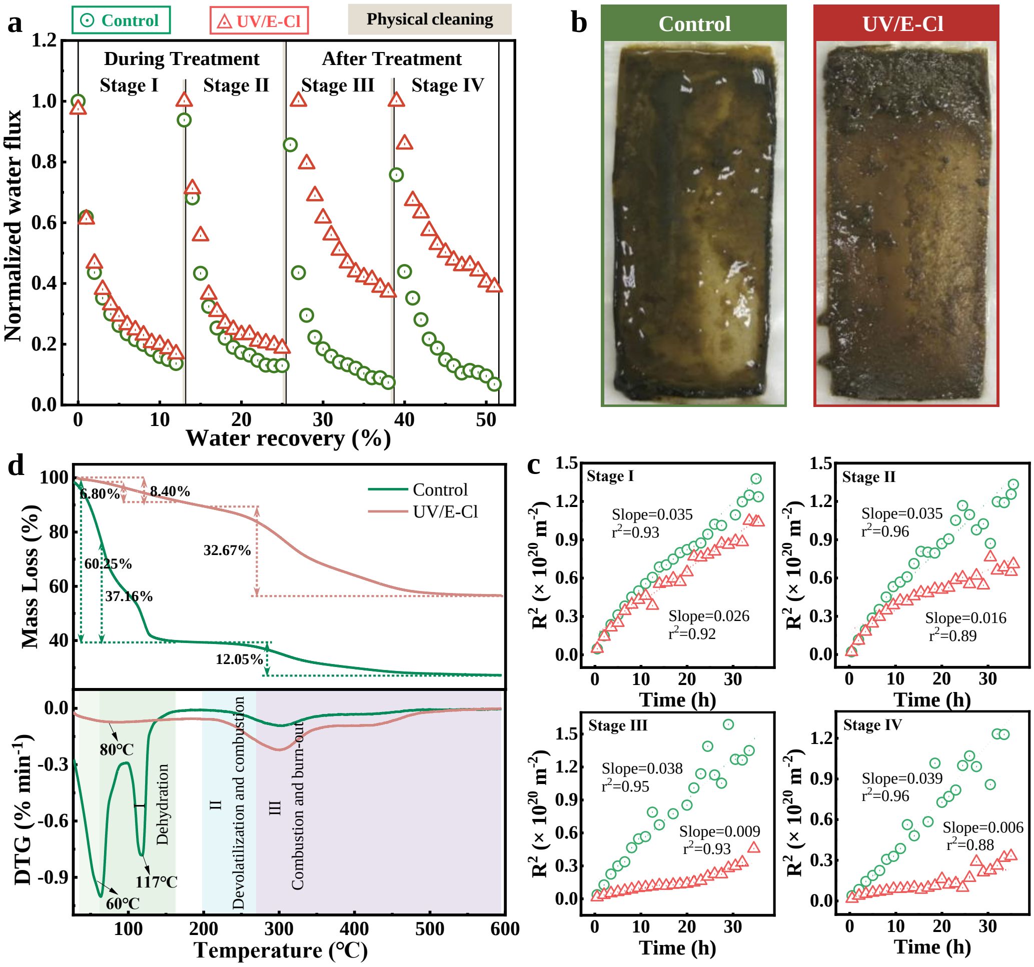

Fig. 1 Dewatering performance of UV/E-Cl assisted FO process for actual WAS: a normalized water flux; b direct observation of foulant layers; c thermal stabilities of foulant layers; and d filtration resistance. (Please see Fig. 9 in the revised manuscript).

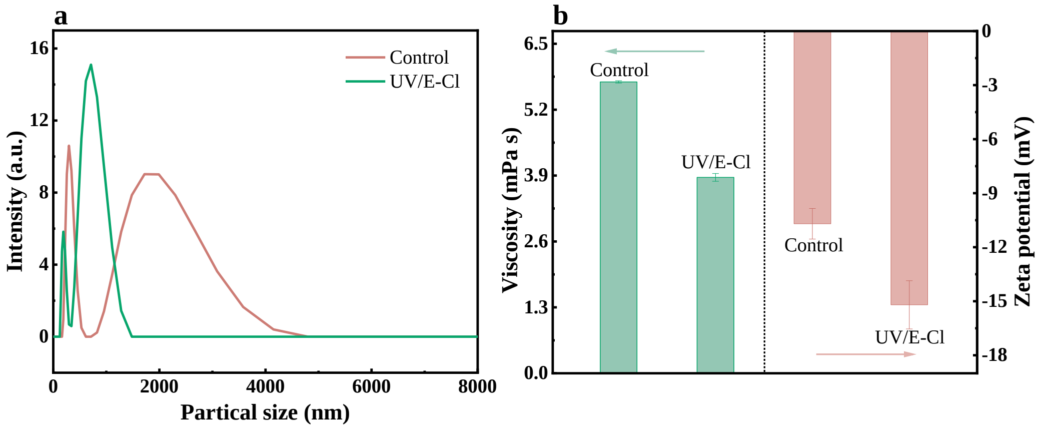

Fig. 2 UV/E-Cl effects on **a** particle size distribution and **b** apparent viscosity and zeta potential of EPS.

EPS size was significantly reduced from 1019 to 562 nm after UV/E-Cl treatment (Fig. 2a). The apparent viscosity was reduced from 5.74 to 3.86 mPa·s, and the zeta potential of EPS solution became more negative value from −10.68 to −15.23 mV (Fig. 2b). These results indicated that UV/E-Cl treatment was efficient in breaking down the entangled network of the EPS matrix, in which the organic complexes formed by the biopolymers bridging each other were readily decomposed[11]. (Please see Fig. S3 in the revised supplementary information).

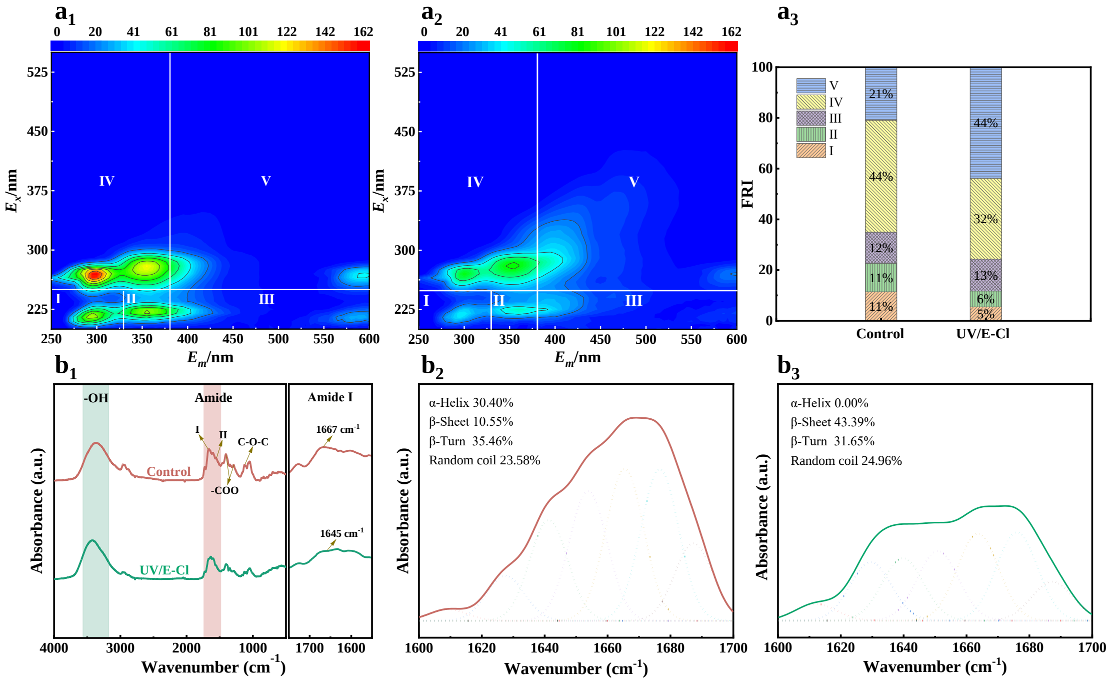

Fig. 3 a EEM spectra (a_1 refers to the control, a_2 refers to the UV/E-Cl and a_3 refers to FRI distribution); and **b** FTIR analysis (b_1 refers to FTIR spectra in the control and UV/E-Cl, b_2 refers to amide I fitting curves in the control and b_3 refers to amide I fitting curves in the UV/E-Cl) of EPS.

EEM spectra of EPS solution were analyzed using fluorescence regional integration (FRI) method by being divided into five excitation–emission regions (Fig. 3a_1 and a_2)[12]. Generally, regions I and II (E_x<250 nm, E_m<350 nm) are associated with simple aromatic proteins such as tyrosine and tryptophan[13]. Region III (200 nm<E_x<250 nm, E_m>380 nm) represents fulvic acid-like substances[14]. Region IV (250 nm<E_x<280 nm, E_m<380 nm) is related to soluble microbial by-product-like material (including microbial polysaccharides)[15-17]. Region V (E_x>280 nm, E_m>380 nm) is

related to humic acid-like organics[14,18]. Regions I, II and III in UV/E-Cl process were smaller than in the control (Fig. 3a$_3$), implying that proteins and polysaccharide-like substances in EPS were targeted to be degraded.

FTIR spectra were further analyzed to acquire more characteristic information on proteins and polysaccharides in EPS (Fig. 3b$_1$). With UV/E-Cl treatment, the intensity of amide I band decreased with blueshift from 1667 to 1645 cm^{-1} (see enlargement of amide I for details), and amide II band disappeared. Additionally, the intensity of C-O-C band significantly decreased. These results reconfirmed that UV/E-Cl effectively disrupted proteins and polysaccharides by attacking glycosidic and peptide bonds mainly, respectively, during the actual WAS dewatering process. Moreover, protein secondary structure showed that the protein helix structure was completely unfolded, as evidenced by the disappearance of α-helix and the decrease in α-helix/ (β-sheet + random coil) value from 89.07 to 0.00% (Fig. 3b$_2$ and b$_3$). Accordingly, the hydrophobic sites or groups inside proteins were easily exposed[19]. (Please see Fig. S4 in the revised supplementary information).

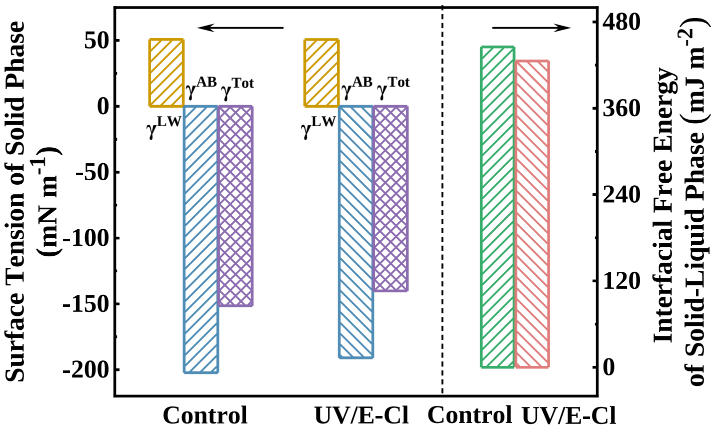

Fig. 4 EPS surface tension and interfacial free energy with water-phase.

With UV/E-Cl process, polar item (γ^{AB}) absolute value decreased from 202.24 to 190.93 mN·m^{-1}, and solid-liquid phase interfacial free energy decreased from 445.29 to 425.69 mJ·m^{-2}. (Please see Fig. S5 in the revised supplementary information).

2. L345-346. Why was a higher concentration of NaCl (4 M) used as the draw solution in this experiment, considering that the NaCl concentration in natural

seawater typically ranges from 0.5 M to 1.0 M? Additionally, why was a 1,000 mg/L SA-BSA mixture used for the model sludge concentration in these experiments?

ANSWER: Thank you for your comments. With regards to your first confusion: in order to amplify the experimental phenomena and to shorten the observation time, we chose a higher concentration of 4 M NaCl as the draw solution in these experiments, as with other studies[23,24]. We have supplemented actual sludge dewatering experiments with a low concentration of 1 M NaCl as draw solution, demonstrating the feasibility of this system over a wide range of NaCl concentrations. (Please see lines 368-374 in the revised manuscript).

Regarding your second confusion, proteins and polysaccharides are recognized as key components that induce membrane gel fouling and limit sludge dewatering performance[21,25]. SA and BSA are commonly used as model substances due to their similar properties and fouling behaviors to proteins and polysaccharides[26,27]. In general, proteins and polysaccharides account for 7%-32% of the mixed liquor suspended solids (MLSS) of sludge[28,29]. Based on the fact that sludge MLSS concentration is usually from 3000 [30,31] to 8000[31-33] $mg\cdot L^{-1}$, the total concentration of polysaccharides and proteins ranges from 210 to 2560 $mg\cdot L^{-1}$. Accordingly, the average value of 1000 $mg\cdot L^{-1}$ SA-BSA mixture was chosen as the model sludge concentration in these experiments. Above information has been added. (Please see text S1 in the revised supplementary information).

3. L 125. The reverse salt (Cl) flux and free chlorine concentration are the most important factors in this experiment. Therefore, the author should provide reverse salt flux and free chlorine data for each experimental case (UV/E-Cl, E-Cl, control, UV).

ANSWER: Thank you for your suggestion. Cl^- may be partially consumed during UV/E-Cl, E-Cl and UV operation, making it difficult to accurately assess reverse Cl^- flux. It has been reported that there is an equal amount of reverse Cl^- for the same water recovery[34]. Therefore, the reverse Cl^- concentration varied with water recovery in the control was determined as a representative indicator to assess the feasibility of constructing an in-situ UV/E-Cl process. With regards to your suggestion, we have

added free chlorine generation for all the control, UV, E-Cl and UV/E-Cl processes (Fig. 5). The control and UV process could not generate free chlorine. Compared with E-Cl process, ~60% chlorine decrease was observed during UV/E-Cl process, suggesting that ~60% free chlorine was subsequently photolyzed by UV irradiation. (Please see Fig. 1c and lines 101-104 in the revised manuscript).

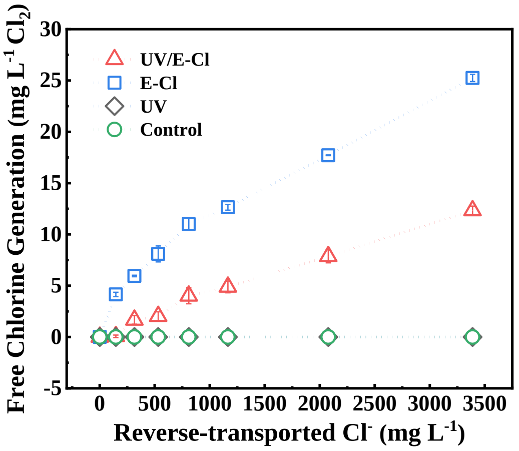

Fig. 5 Free chlorine generation during UV/E-Cl, E-Cl, control and UV processes at 7.84 mA·cm^{-2} current density. UV conditions: power = 10 W, fluence rate = 0.44 mW·cm^{-2}.

4. L136. I confused the conditions with treatment and without treatment. How could higher performance be achieved without treatment in Figure 2a?

ANSWER: Thank you for pointing this out. There is an inaccurate expression here. "With treatment" and "without treatment" should be corrected more accurately to "during treatment" and "after treatment", respectively (Please see Fig. 2a in the revised manuscript). From stage I to stage IV was a continuous process with treatment carried out in stages I and II ("during treatment"). After successive stages I and II treatments, more membrane fouling precursor substances were destroyed, resulting in better performance shown in stages III and IV ("after treatment"). The clarification has been added to avoid confusion (Please see lines 128-130 in the revised manuscript).

5. L159. The floc size decreased after the UV/E-Cl treatment. Generally, a smaller

floc size could accelerate the formation of a more dense fouling layer compared to larger flocs. However, the experimental results contradict this observation. Why?

ANSWER: Thank you for your comment. We strongly recognize the general view “a smaller floc size could accelerate the formation of a denser fouling layer compared to larger flocs”, especially in dead-end filtration system, where flocs inevitably end up trapped/deposited on membrane surface, with smaller flocs filling the voids and accelerating the formation of a denser fouling layer. However, unlike dead-end filtration process, deposition of flocs on membrane surface in a cross-flow FO filtration system may face probabilistic problem[35]. In this study, XDLVO calculations revealed that smaller flocs had a lower adhesion energy to membrane surface compared with larger flocs, which suggested that smaller flocs were less likely to contact with membrane surface (Please see Fig. 5 in the revised manuscript). This result is consistent with previous studies which claimed that large flocs approach the membrane surface more easily[36,37]. In addition, decrease in flocs viscosity further weakened the adhesion tendency to membrane surface. As a result, insufficient small flocs were deposited on membrane surface after UV/E-Cl treatment, so that a denser fouling layer was not observed.

6. L254. I cannot find information about Figures 7a and 7b in the manuscript. Additionally, were the DFT simulations conducted by the authors, or were the results cited from other works? The author should provide a more detailed explanation about the DFT simulations.

ANSWER: Thank you for your reminder. a and b have been labeled. (Please see Fig. 6 in the revised manuscript).

DFT simulations were conducted by ourselves. The details of the simulation are as follows: “DFT simulations were performed to explore the interactions between SA and BSA for providing molecular level clues to membrane fouling behaviors. A single alginate chain consisting of 5 uronic rings and an amino acid fragment with a trimer of lysine, aspartic acid and glycine were constructed by GaussView 6.0 as the SA and BSA models, respectively[38,39]. Molecular geometries between two alginate chains and

a BSA fragment were optimized by B3LYP hybrid functional combined with 6-31G(d,p) basis set to obtain more accurate interaction energies. DFT-D3 dispersion correction was taken into account in all calculations." (Please see "Text S2. 1.3 Density functional theory (DFT) calculation" in the revised supplementary information).

More detailed explanation has been added as follows: "Density functional theory (DFT) simulations were further performed to explain this specific cross-linked network structure. Simulation results showed three optimized binding models between SA and BSA with relative energies. In detailed, the NH_3^+ of BSA could strongly coordinate with terminal COO^- of SA (BS1), or with terminal and non-terminal COO^- (BS2), and or with non-terminal COO^- (BS3). It is clear that the relative energies follow the order BS1 < BS2 < BS3, suggesting BS1 configuration is more likely to occur. Energetically, BS1 shows higher stability because the SA chains are as dispersed as possible to minimize the electrostatic repulsion interactions[39]" (Please see lines 251-259 in the revised manuscript).

7. L281-286. The explanation is quite abstract, and a more direct explanation should be provided based on the experimental results.

ANSWER: Thank you for pointing this out. The sentences have been rewritten based on the experimental results as follows: "Therefore, in the control, a homogeneous gel-like layer formed (Fig. 6a_1), with the pores occupied by abundant bound water (Fig. 7c_1). Conversely, after UV/E-Cl treatment, a loose and dried cake-like foulant layer (Fig. 6a_2) with unfilled pores (Fig. 7c_2) was formed.". (Please see lines 277-280 in the revised manuscript).

8. L292-335. The proposed mechanism illustrates changes in the formation of the organic fouling layer by the UV/E-Cl system, but it does not focus on the aspects of dewatering waste activated sludge. As I asked in Q1, the experimental concept is closer to the control of organic fouling in FO rather than the sludge dewatering system.

ANSWER: Thank you for this constructive comment. We have realized that the experimental concept of this work might be closer to controlling organic membrane fouling during dewatering of waste activated sludge by the FO process. The manuscript has been refined to investigate the performance and underlying mechanism of the novel in-situ UV/E-Cl in controlling typical organic fouling caused by polysaccharides and proteins during WAS dewatering by FO process. Furthermore, we have conducted actual sludge dewatering experiments to validate the core claims of our work to improve WAS dewatering performance as shown in Q1. It should be noted that although our work was carried out with WAS, the proposed mechanism that water occurrence states dominate membrane gel fouling is of universal significance for gel fouling mitigation and membrane process operation, independent of wastewater type, membrane model type and reactor scale.

Reference

1. Hua, Z. et al. Exploring pathways and mechanisms for dichloroacetonitrile formation from typical amino compounds during UV/chlorine treatment. *Environ. Sci. Technol.* **56**, 9712-9721 (2022).
2. Bulman, D. M. & Remucal, C. K. Role of reactive halogen species in disinfection byproduct formation during chlorine photolysis. *Environ. Sci. Technol.* **54**, 9629-9639 (2020).
3. Allen, J. M. et al. Drivers of disinfection byproduct cytotoxicity in U.S. drinking water: Should other DBPs be considered for regulation? *Environ. Sci. Technol.* **56**, 392-402 (2022).
4. WHO. Guidelines for drinking-water quality, 3rd edition: Volume 1-Recommendations incorporating the first and second addenda, Geneva (2008).
5. Wang, X., Mao, Y., Tang, S., Yang, H. & Xie, Y. F. Disinfection byproducts in drinking water and regulatory compliance: A critical review. *Front. Environ. Sci. Eng.* **9**, 3-15 (2015).
6. Guo, Y. et al. Variations of heavy metals, nutrients, POPs and particle size distribution during "sludge anaerobic digestion-solar drying-land utilization process": Case study in China. *Sci. Total. Environ.* **801**, 149609 (2021).
7. Xu, J., Tran, T. N., Lin, H. & Dai, N. Modeling the transport of neutral disinfection byproducts in forward osmosis: Roles of reverse salt flux. *Water Res.* **185**, 116255 (2020).
8. Coday, B. D., Yaffe, B. G. M., Xu, P. & Cath, T. Y. Rejection of trace organic compounds by forward osmosis membranes: A literature review. *Environ. Sci. Technol.* **48**, 3612-3624 (2014).
9. Lee, H., Im, S.-J., Park, J. H. & Jang, A. Removal and transport behavior of trace organic compounds and degradation byproducts in forward osmosis process: Effects of operation conditions and membrane properties. *Chem. Eng. J.* **375**, 122030 (2019).
10. Wang, H. et al. Improved understanding of dissolved organic matter

transformation in concentrated leachate induced by hydroxyl radicals and reactive chlorine species. *J. Hazard. Mater.* **387**, 121702 (2020).

11. Zhang, W. et al. Influence of wastewater sludge treatment using combined peroxyacetic acid oxidation and inorganic coagulants re-flocculation on characteristics of extracellular polymeric substances (EPS). *Water Res.* **88**, 728-739 (2016).
12. Chen, W., Westerhoff, P., Leenheer, J. A. & Booksh, K. Fluorescence excitation−emission matrix regional integration to quantify spectra for dissolved organic matter. *Environ. Sci. Technol.* **37**, 5701-5710 (2003).
13. Ahmad, S. R. & Reynolds, D. M. Monitoring of water quality using fluorescence technique: Prospect of on-line process control. *Water Res.* **33**, 2069-2074 (1999).
14. Mounier, S., Patel, N., Quilici, L., Benaim, J. Y. & Benamou, C. Fluorescence 3D de la matière organique dissoute du fleuve amazone: (Three-dimensional fluorescence of the dissolved organic carbon in the Amazon river). *Water Res.* **33**, 1523-1533 (1999).
15. Coble, P. G. Characterization of marine and terrestrial DOM in seawater using excitation-emission matrix spectroscopy. *Mar. Chem.* **51**, 325-346 (1996).
16. Reynolds, D. M. & Ahmad, S. R. Rapid and direct determination of wastewater BOD values using a fluorescence technique. *Water Res.* **31**, 2012-2018 (1997).
17. Wang, B.-B. et al. A new classification paradigm of extracellular polymeric substances (EPS) in activated sludge: Separation and characterization of exopolymers between floc level and microcolony level. *Water Res.* **64**, 53-60 (2014).
18. Artinger, R. et al. Characterization of groundwater humic substances: Influence of sedimentary organic carbon. *Appl. Geochem.* **15**, 97-116 (2000).
19. You, G. et al. Insights into the short-term effects of CeO_2 nanoparticles on sludge dewatering and related mechanism. *Water Res.* **118**, 93-103 (2017).
20. Duc Viet, N., Im, S.-J. & Jang, A. Characterization and control of membrane fouling during dewatering of activated sludge using a thin film composite forward osmosis membrane. *J. Hazard. Mater.* **396**, 122736 (2020).

21. Yi, X., Zhong, H., Xie, M. & Wang, X. A novel forward osmosis reactor assisted with microfiltration for deep thickening waste activated sludge: Performance and implication. *Water Res.* **195**, 116998 (2021).

22. Yi, X. et al. Novel insights on fouling mechanism of forward osmosis membrane during deep thickening waste activated sludge. *J. Membr. Sci.* **660**, 120894 (2022).

23. Mi, B. & Elimelech, M. Gypsum scaling and cleaning in forward osmosis: Measurements and mechanisms. *Environ. Sci. Technol.* **44**, 2022-2028 (2010).

24. Li, L. et al. In situ extracting organic-bound calcium: A novel approach to mitigating organic fouling in forward osmosis treating wastewater via gradient diffusion thin-films. *Water Res.* **156**, 102-109 (2019).

25. Wu, B., Dai, X. & Chai, X. Critical review on dewatering of sewage sludge: Influential mechanism, conditioning technologies and implications to sludge re-utilizations. *Water Res.* **180**, 115912 (2020).

26. Lin, Y., de Kreuk, M., van Loosdrecht, M. C. M. & Adin, A. Characterization of alginate-like exopolysaccharides isolated from aerobic granular sludge in pilot-plant. *Water Res.* **44**, 3355-3364 (2010).

27. You, X. et al. Metal-coordinated sub-10 nm membranes for water purification. *Nat. Commun.* **10**, 4160 (2019).

28. Hu, G., Liu, X., Wang, Z., Du, X. & Wang, X. Comparison of fouling behaviors between activated sludge suspension in MBR and EPS model solutions: A new combined model. *J. Membr. Sci.* **621**, 119020 (2021).

29. Basuvaraj, M., Fein, J. & Liss, S. N. Protein and polysaccharide content of tightly and loosely bound extracellular polymeric substances and the development of a granular activated sludge floc. *Water Res.* **82**, 104-117 (2015).

30. Yi, X., Wang, Z., Zhao, P., Song, W. & Wang, X. New insights on destruction mechanisms of waste activated sludge during simultaneous thickening and digestion process via forward osmosis membrane. *Water Res.* **254**, 121378 (2024).

31. Nguyen, N. C., Chen, S.-S., Yang, H.-Y. & Hau, N. T. Application of forward osmosis on dewatering of high nutrient sludge. *Bioresour. Technol.* **132**, 224-229 (2013).

32. Hau, N. T. et al. Exploration of EDTA sodium salt as novel draw solution in forward osmosis process for dewatering of high nutrient sludge. *J. Membr. Sci.* **455**, 305-311 (2014).
33. Nguyen, N. C., Nguyen, H. T., Chen, S.-S., Nguyen, N. T. & Li, C.-W. Application of forward osmosis (FO) under ultrasonication on sludge thickening of waste activated sludge. *Water Sci. Technol.* **72**, 1301-1307 (2015).
34. Tang, C. Y., She, Q., Lay, W. C. L., Wang, R. & Fane, A. G. Coupled effects of internal concentration polarization and fouling on flux behavior of forward osmosis membranes during humic acid filtration. *J. Membr. Sci.* **354**, 123-133 (2010).
35. Liu, J. et al. Stochastic collision–attachment-based Monte Carlo simulation of colloidal fouling: Transition from foulant–clean-membrane interaction to foulant–fouled-membrane interaction. *Environ. Sci. Technol.* **54**, 12703-12712 (2020).
36. Shen, L.-g. et al. Membrane fouling in a submerged membrane bioreactor: Impacts of floc size. *Chem. Eng. J.* **269**, 328-334 (2015).
37. Yu, Z., Song, Z., Wen, X. & Huang, X. Using polyaluminum chloride and polyacrylamide to control membrane fouling in a cross-flow anaerobic membrane bioreactor. *J. Membr. Sci.* **479**, 20-27 (2015).
38. De Luca, G., Bisignano, F., Paone, F. & Curcio, S. Multi-scale modeling of protein fouling in ultrafiltration process. *J. Membr. Sci.* **452**, 400-414 (2014).
39. Zhang, M. et al. Mechanistic insights into alginate fouling caused by calcium ions based on terahertz time-domain spectra analyses and DFT calculations. *Water Res.* **129**, 337-346 (2018).

Responses to Comments and Suggestions of Reviewers

Re manuscript: NCOMMS-23-55103A-Z.

Title: Dynamic reverse Cl^- driven integration of sludge conditioning and dewatering: Emphasis on water occurrence states.

ANSWER: The authors would like to thank the editor and the reviewers for their kind consideration on our revised manuscript. We greatly value the critical and constructive comments from the reviewers and agree that the suggested revisions will significantly enhance the quality and depth of our research. The manuscript has been modified to address the comments and the changes in the revised manuscript and supplementary information are highlighted in red. The following are our point-to-point responses to all the reviewers' comments.

Responses to Reviewer #1 's comments:

General comments:

The authors have solved most of my comments and revised the manuscript. They proved the concept by using real wastewater sludge. However, there still some issues should be considered. The major aspect is the author should report the results of real sludge rather than SB-BSA in the main text.

ANSWER: We sincerely appreciate your positive feedback on the revised manuscript and greatly value your critical and constructive comments. We agree that the suggested revisions will significantly enhance the quality and depth of our research. The manuscript has been significantly modified according to your following issues and suggestions.

We understand your perspective that the results of real sludge should be reported in the main text. While we agree that the results from the real sludge experiments are essential to demonstrate the practical applicability of our approach, we believe that reporting the results from the SA-BSA model system in the main text remains crucial for several reasons. Firstly, the compositional complexity of real sludge makes it challenging to isolate and definitively identify the specific mechanisms driving the mitigated gel fouling and enhanced dewatering performance. By utilizing the

simplified and typical SA-BSA model system, we were able to gain a deeper understanding of the underlying mechanisms through quantum mechanical calculations and filtration thermodynamic theory. These insights provide a foundation for understanding the more complex real sludge system. Secondly, the detailed analysis of the SA-BSA model system provides valuable insights into the fundamental interactions between the membrane, draw solution, and sludge components, which are not readily observable in the real sludge system. These fundamental insights are critical for developing more effective and targeted solutions for sludge dewatering.

Although SA-BSA results are reported in the main text, we highly value the real sludge results. We have added real sludge dewatering experiments in UV/E-Cl assisted FO process using higher NaCl draw solution (4 M) to verify the high feasibility of this system to alleviate gel-like fouling and improve sludge dewatering performance over a wide range of NaCl concentrations. We hope that our explanations will satisfy your concerns and gain your understanding and support.

Specific comments:

1. The authors proved that UV/E-Cl could alleviate FO membrane fouling, and increased water flux. My major concern is whether the highly oxidative chlorine and radicals will destroy the membrane or reduce its effective time. The authors should characterize the membrane material after using several cycles.

ANSWER: Thank you for your crucial comment. We totally understand your concern about the membrane performance after using in UV/E-Cl processes. We have complemented the comparison of deionized water flux, reverse salt diffusion, membrane chemistry and surface morphology of a new pristine membrane and the used membrane after 4-cycle UV/E-Cl processes. Fig. 1a shows that initial water flux and reverse salt accumulation of the two membranes were comparable. FTIR spectra and SEM images revealed no tangible changes in functional groups and surface morphology on pristine membrane and used membrane (Fig. 1b-f). These findings suggest that membrane performances of water flux, reverse salt and microstructural integrity were not significantly compromised by UV/E-Cl treatment. As for the

overall relatively lower water flux of the used membrane, it was reasonable and attributed to membrane fouling, as confirmed by the foulants remaining on the selective layer in the SEM image (inset figure in Fig. 1d). (Please see lines 359-369 in the revised manuscript).

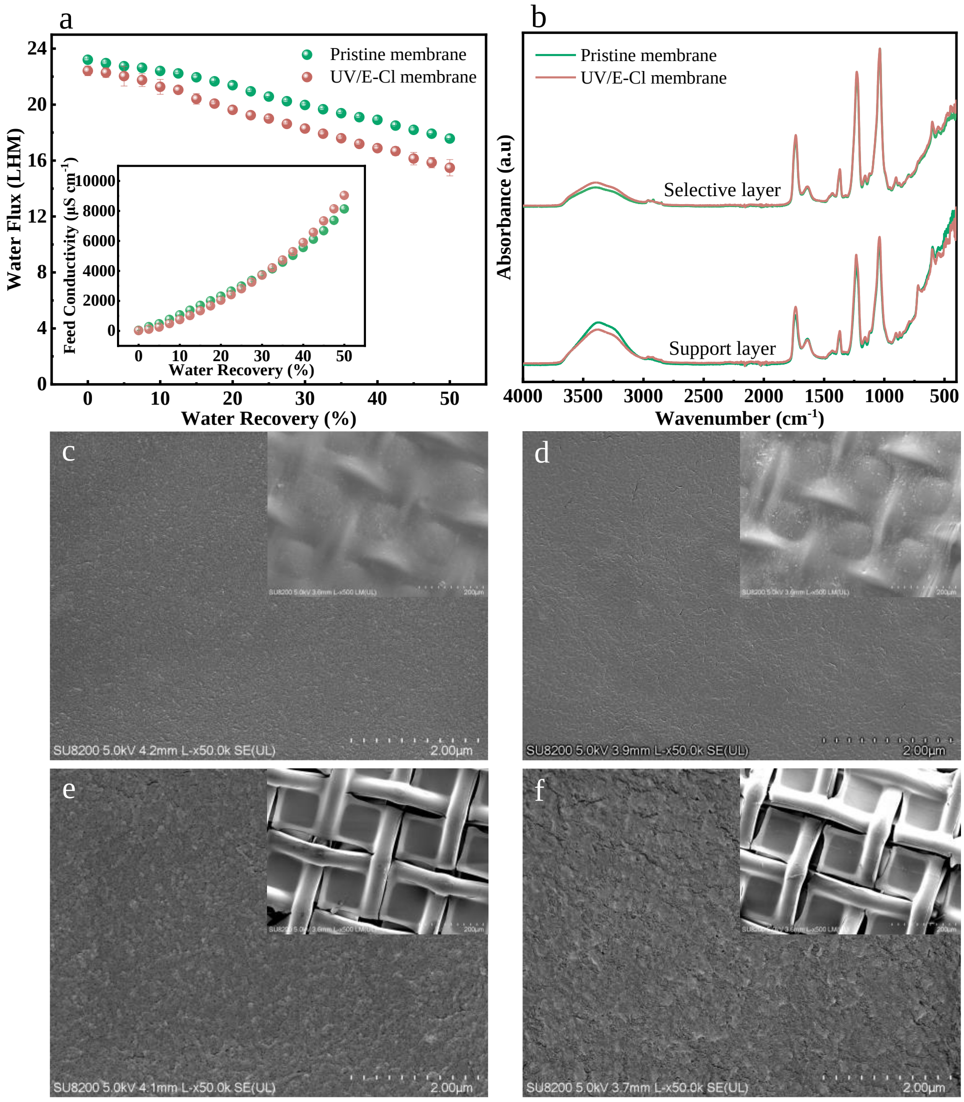

Fig. 1 **a** comparison in water flux and feed conductivity between the pristine membrane and the used membrane after 4-cycle UV/E-Cl processes using deionized water as feed solution and 4 M NaCl as draw solution; **b** FTIR absorbance spectra at wavenumbers of 4000-500 cm^{-1} for the selective layer and the support layer of the pristine membrane and the used membrane; The SEM images showing **c-d.** the selective layer of a pristine membrane and the used membrane, respectively; **e-f.** the support layer of a pristine membrane and the used membrane, respectively.

2. The DBP formation from real wastewater sludge should be evaluated, as this case is totally different from the SA-BSA case.

ANSWER: Thank you for pointing this out. We sincerely apologize for the lack of clarity in the presentation leading to your misunderstanding. We have strengthened the explicit statement to make it easier to get that DBP formation was evaluated from real wastewater sludge. The following statements have been rewritten and supplemented: "The DBPs formation in the feed and draw solutions during actual WAS dewatering process with UV/E-Cl treatment was further investigated." (Please see lines 369-372 in the revised manuscript). "Analysis of DBPs in **a** feed solution and **b** draw solution of actual WAS treated with UV/E-Cl process." (Please see Fig. S9 in the revised supplementary information).

3. The authors used EPR and NB to prove the effects radicals. However, EPR cannot confirm the signal of Cl*. When high concentrations of NB was used to scavenge HO*, NB was filter most UV light simutaneously. Thus, spiking NB cannot prove the roles of either HO* or Cl*.

ANSWER: Thank you so much for your valuable and thoughtful comment. It has been reported that Cl• can be observed with a characteristic nine-line EPR spectra[1]. The same characteristic EPR spectrum was observed, demonstrating the possible presence of Cl• during UV/E-Cl process (Fig. 2). Moreover, to further confirmed the presence of Cl•, we have conducted subsequent probe experiments. The reference has been incorporated into manuscript (Please see reference [35] in the revised manuscript) and EPR spectra have been revised to accurately label the characteristic peak shapes of Cl• (Fig. 2) (Please see Fig. 1d in the revised manuscript).

Additionally, we understand your concern and have recognized the limitations of using high concentration of NB as a quencher in UV system. We have modified the experiment to study the competition kinetics of coexisting BSA, SA and low concentration of benzoic acid (BA) and NB (0.01 mM) to distinguish the respective contributions of HO• and Cl•[2]. BA reacts rapidly with both HO• and Cl•, with rate constants of $k_{HO•,BA} = 5.9 \times 10^{9}$ M^{-1} s^{-1} and $k_{Cl•,BA} = 1.8 \times 10^{10}$ M^{-1} s^{-1}, respectively.

In contrast, NB reacts rapidly with HO• ($k_{HO•,NB}$ = 3.9 × 10^9 M^{-1} s^{-1}) but very slowly with Cl• ($k_{Cl•,NB}$ < 10^6 M^{-1} s^{-1})[3,4]. Considering that UV/E-Cl process involves stage I and stage II with different ratios of free chlorine and Cl^-, we conducted two simulated degradation processes regarding stage I and stage II. Following the steady-state assumption, the pseudo-first-order rate constants of the degradation of NB (k_{NB}), BA (k_{BA}), BSA (k_{BSA}) and SA (k_{SA}) along with the steady-state concentrations of HO• ($[HO•]_{ss}$) and Cl• ($[Cl•]_{ss}$) can be given by Eqs. 1-4, respectively[2] (Fig. 3a-b, Table 1). The rate constants of the HO• reaction with BSA ($k_{HO•, BSA}$) and SA ($k_{HO•, SA}$) were estimated by the competition kinetics of BSA, SA and BA degradation in the UV/H_2O_2 system (Eqs. 5-6 and Fig. 3c-d). Accordingly, the secondary-order rate constants between Cl• and BSA ($k_{Cl•, BSA}$) and between Cl• and SA ($k_{Cl•, SA}$) were obtained (Eqs. 3-4). The relative contributions of Cl• to BSA and SA degradation were estimated to be more than 90% in both stage I and stage II, demonstrating that Cl• is the dominant reactive species, almost fully accounting for the decomplexation of SA and BSA in the process. The figures and related information have been added. (Please see lines 147-152 in the revised manuscript and Text S2, Fig. S1 and Table S2 in the revised supplementary information).

It is important to note that the complexity of the sludge makes it difficult to reasonably and accurately assess the relative contributions of HO• and Cl• to sludge dewatering. Considering that proteins and polysaccharides are recognized as key components in the formation of membrane gel layer and in limiting sludge dewatering, we conducted a degradation kinetic analysis related to polysaccharides and proteins. Based on the results, it is evident that HO• in the UV/E-Cl system have almost negligible degradation capacity for proteins and polysaccharides, laterally confirming the limited contribution of HO• to sludge dewatering. We desire the above analyses will address your concerns and get your approval.

$$k_{NB} = k_{HO•,NB}[HO•]_{ss} \quad (1)$$

$$k_{BA} = k_{HO•,BA}[HO•]_{ss} + k_{Cl•,BA}[Cl•]_{ss} \quad (2)$$

$$k_{BSA} = k_{HO•,BSA}[HO•]_{ss} + k_{Cl•,BSA}[Cl•]_{ss} \quad (3)$$

$$k_{\mathrm{SA}} = k_{\mathrm{HO\bullet,SA}}\left[\mathrm{HO\bullet}\right]_{ss} + k_{\mathrm{Cl\bullet,SA}}\left[\mathrm{Cl\bullet}\right]_{ss} \tag{4}$$

$$\ln\frac{[BSA]}{[BSA]_0} = \frac{k_{\mathrm{HO\bullet,BSA}}}{k_{\mathrm{HO\bullet,BA}}} \times \ln\frac{[BA]}{[BA]_0} \tag{5}$$

$$\ln\frac{[SA]}{[SA]_0} = \frac{k_{\mathrm{HO\bullet,SA}}}{k_{\mathrm{HO\bullet,BA}}} \times \ln\frac{[BA]}{[BA]_0} \tag{6}$$

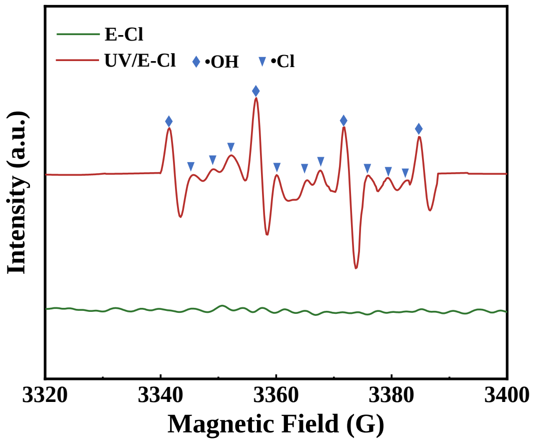

Fig. 2 EPR spectra of different radicals during E-Cl and UV/E-Cl processes (◊ and ▽ represent the typical peak profile of HO• and Cl•, respectively).

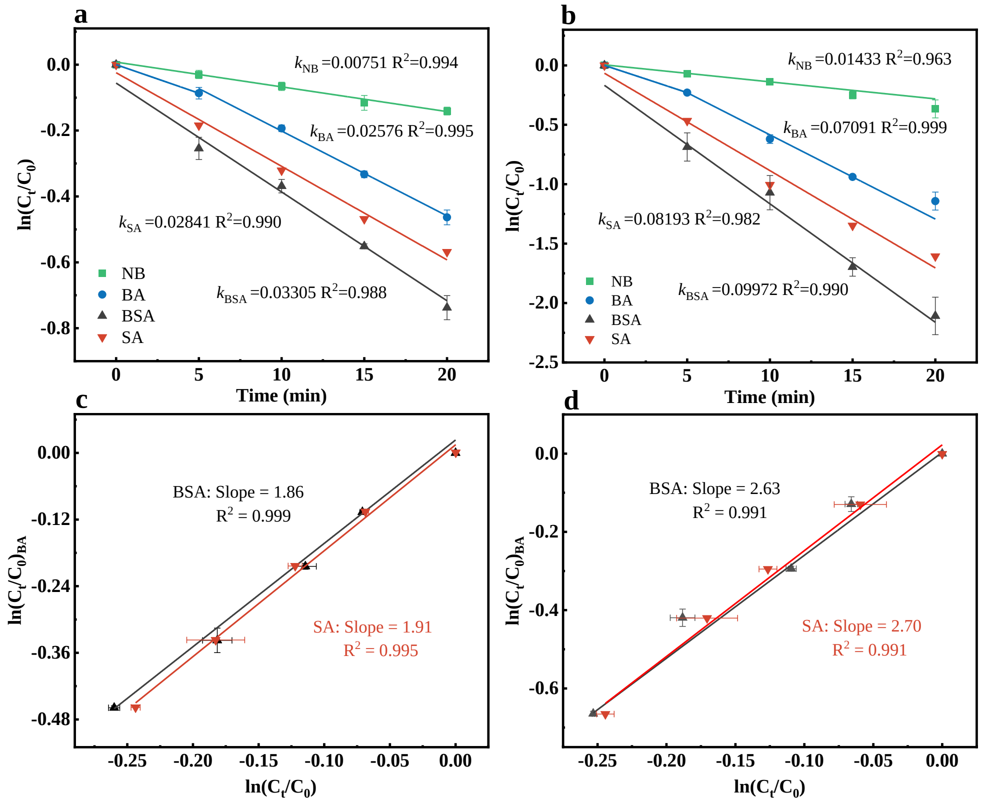

Fig. 3 a degradation kinetics of the coexisting NB, BA, BSA and SA in the UV/E-Cl process refer to stage I. Conditions: 25 °C, $[HClO]_0$ = 0.30 mM, $[Cl^-]_0$ = 264 mg·L^{-1},

$[BSA]_0$ = $[SA]_0$ = 50 mg·L^{-1}, $[BA]_0$ = $[NB]_0$ = 0.01 mM, UV fluence rate = 0.44 mW·cm^{-2}; **b** degradation kinetics of the coexisting NB, BA, BSA and SA in the UV/E-Cl process refer to stage II. Conditions: 25 °C, $[HClO]_0$ = 0.6 mM $[Cl^-]_0$ = 823 mg·L^{-1}, $[BSA]_0$ = $[SA]_0$ = 50 mg·L^{-1}, $[BA]_0$ = $[NB]_0$ = 0.01 mM, UV fluence rate = 0.44 mW·cm^{-2}; **c** competition kinetics between BA, BSA and SA in the UV/H_2O_2 system. Condition: 25 °C, $[H_2O_2]_0$ = 0.30 mM, $[Cl^-]_0$ = 264 mg·L^{-1}, $[BSA]_0$ = $[SA]_0$ = 50 mg·L^{-1}, $[BA]_0$ = $[NB]_0$ = 0.01 mM, UV fluence rate = 0.44 mW·cm^{-2}; **d** competition kinetics between BA, BSA and SA in the UV/H_2O_2 system. Condition: 25 °C, $[H_2O_2]_0$ = 0.60 mM, $[Cl^-]_0$ = 823 mg·L^{-1}, $[BSA]_0$ = $[SA]_0$ = 50 mg·L^{-1}, $[BA]_0$ = $[NB]_0$ = 0.01 mM, UV fluence rate = 0.44 mW·cm^{-1}.

Table 1. The competition kinetics results during BSA and SA degradation by UV/E-Cl using NB and BA as radical probes. Conditions: 25°C, $[BSA]_0$ = $[SA]_0$ = 50 mg·L^{-1}, $[BA]_0$ = $[NB]_0$ = 0.01 mM.

	Stage I	Stage II
k_{NB} (min^{-1})	0.00751	0.0143
k_{BA} (min^{-1})	0.0258	0.0710
k_{BSA} (min^{-1})	0.0331	0.100
k_{SA} (min^{-1})	0.0284	0.0819
$[HO•]_{ss}$ (M)	3.21×10^{-14}	6.12×10^{-14}
$[Cl•]_{ss}$ (M)	1.33×10^{-14}	4.56×10^{-14}
$k_{HO•, BSA}$ (M^{-1} s^{-1})	3.17×10^{9}	2.24×10^{9}
$k_{Cl•, BSA}$ (M^{-1} s^{-1})	3.37×10^{10}	3.34×10^{10}
$k_{HO•, SA}$ (M^{-1} s^{-1})	3.09×10^{9}	2.19×10^{9}
$k_{Cl•, SA}$ (M^{-1} s^{-1})	2.81×10^{10}	2.70×10^{10}
Cl• contribution on BSA degradation	91%	94%
HO• contribution on BSA degradation	9%	6%
Cl• contribution on SA degradation	90%	93%
HO• contribution on SA degradation	10%	7%

4. The English written should be further improved.

ANSWER: Thank you for your reminder. The manuscript has been re-polished by a professional agency and has been thoroughly revised.

EDITSPRINGS

EDITORIAL CERTIFICATE

This is to certify that the manuscript detailed below was edited by one or more of our highly qualified, native English speakers at EditSprings, to assure compliance with Anglophone academic standards in terms of style, punctuation, grammar, and spelling.

Manuscript title:

Dynamic reverse Cl driven integration of sludge conditioning and dewatering: Emphasis on water occurrence states

Authors:

Xiujia You 1, Hanmin Zhang 1*, Hongjun Lin 2, Linhua Rao 1

Date Issued:

Aug 17 2024

Certificate Number:

ES-202404191139394543

This certificate can be verified on https://www.editsprings.com/QueryCertificate.html . EditSprings hereby certifies that neither content nor the author's intentions were altered in any way during the editing process. Documents in receipt of this certification should be ready for publication as far as style and language are concerned, provided that the author(s) accepted our suggestions and changes (which remains their right and responsibility).

EditSprings offers a wide range of editing, translation, for researchers and publishers across the world. Our highly skilled editors are all established academics based in Anglophone Higher Education institutions across the world (U.K., U.S.A., Canada, Australia, and elsewhere), are experts in their respective fields, and are qualified to edit research papers authored by non-Anglophone scholars.

Responses to Reviewer #2 's comments:

General Comments:

The author successfully incorporated the revisions suggested by the reviewers into the manuscript. However, one question still remains regarding the revised manuscript as follows:

ANSWER: We are very grateful for your approval of the revised manuscript and your valuable comment. We greatly value your concern about the concentration of NaCl draw solutions, and have conducted additional experiments and characterizations to improve the rigor of our work.

Specific comments:

This question pertains to Q2 from the first-round review. The concentration of the draw solution is a critical operating parameter in the FO process. The author provided data using 4 M NaCl as the draw solution. However, the actual sludge dewatering experiment used 1 M NaCl as the draw solution. The difference in draw solution concentration can significantly alter the performance of the FO process, affecting parameters such as water flux, reverse salt flux, and fouling formation. Therefore, these supplementary experimental results do not correspond to the conditions under which 4 M NaCl was used.

ANSWER: Thank you for your insightful and constructive comment. We have carefully considered your comment and quite understand your concern. While we initially chose 1 M NaCl to align with the typical salinity of seawater, we recognize the need to evaluate the actual WAS dewatering performance by FO process using the same draw solution concentration (4 M NaCl) as the main study to increase the rigor of our discussion. Additionally, we retained the results of sludge dewatering experiments using 1 M NaCl as draw solution and incorporated it into the revised manuscript to adequately validate the feasibility of this system to improve FO performance over a wide range of NaCl concentrations. The results of actual sludge dewatering experiments using 4 M NaCl as draw solution are as follows:

"Actual WAS dewatering. Actual WAS dewatering experiments verified that UV/E-

Cl assisted FO process using 4 M NaCl as draw solution effectively improved WAS dewatering flux compared with the control, as evidenced by an average 543% increase in stages III and IV (Fig. 4a). As expected, the foulant layer formed during WAS dewatering changed from a gel-like to a cake-like state (Fig. 4b), in which water content decreased from 64.06% to 9.88% and bound water content dropped by 85.48% (from 44.29% to 6.43%) (Fig. 4c). Correspondingly, filtration resistance decreased by an order of magnitude from R^2_{stageIII} = 2.59×10^{19} and R^2_{stageIV} = 3.36×10^{19} in the control to R^2_{stageIII} = 3.57×10^{18} and R^2_{stageIV} = 2.48×10^{18} (Fig. 4d), with resistance rates dropping from 0.040 and 0.048 to 0.004 and 0.002 m^{-1}, respectively. UV/E-Cl was focused on destructing EPS physicochemical properties, which was initially confirmed by decreased size (Fig. 5a), apparent viscosity and zeta potential (Fig. 5b). Furthermore, EEM spectra indicated that polysaccharides and protein-like substances in EPS were targeted for degradation, as confirmed by a decrease in characteristic fluorescence intensities (Fig. 6a). FTIR spectra suggested that glycosidic and peptide bonds were the main attacked sites in polysaccharides and proteins, respectively, which led to a strong exposure of hydrophobic sites or groups inside proteins (Fig. 6b), corresponding to a reduced surface polarity (Fig. 7).” (Please see lines 324-341 in the revised manuscript).

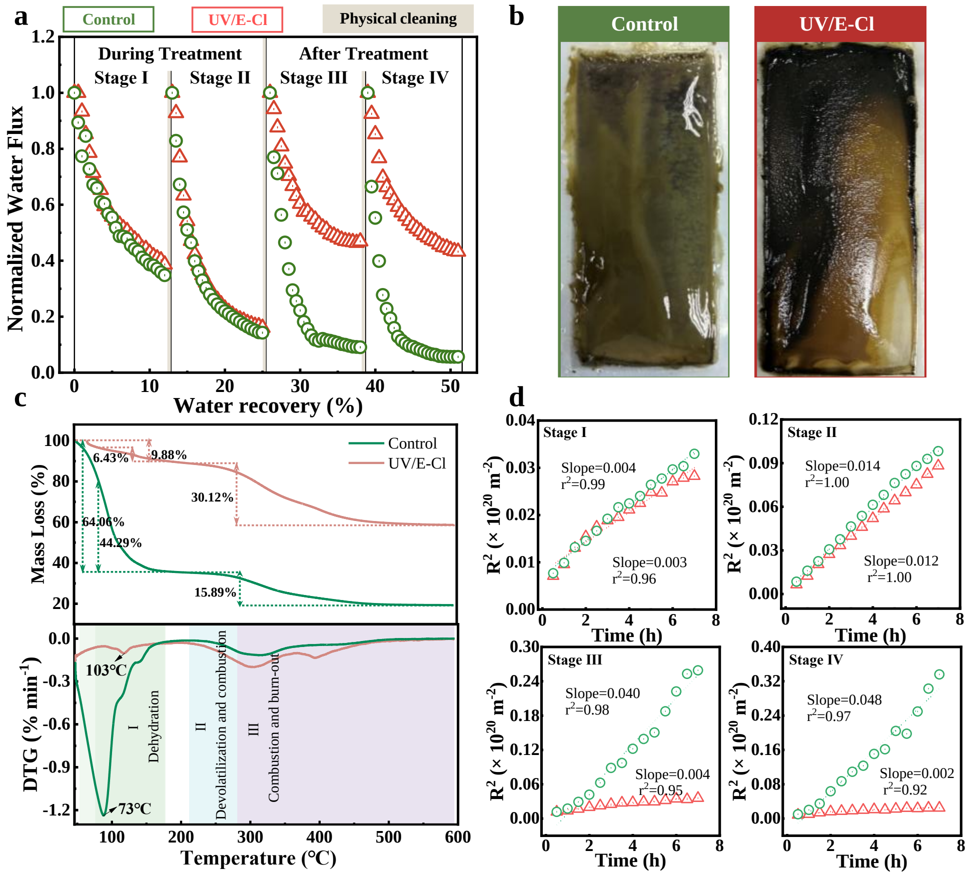

Fig. 4 Dewatering performance of UV/E-Cl assisted FO process for actual WAS: **a** normalized water flux; **b** direct observation of foulant layers; **c** thermal stabilities of foulant layers; and **d** filtration resistance. Conditions: draw solution = 4 M NaCl, current density = 7.84 mA·cm^{-2} and UV fluence rate = 0.44 mW·cm^{-2}. (Please see Fig. 9 in the revised manuscript).

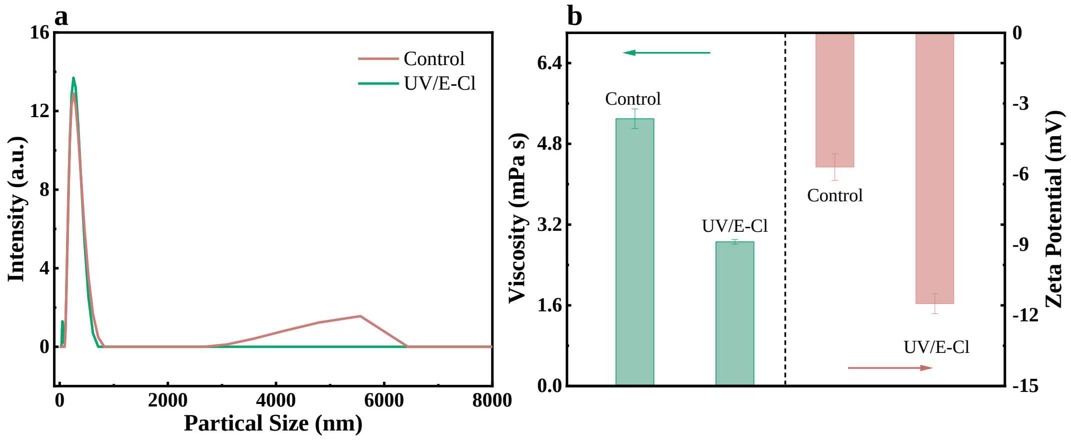

Fig. 5 Effects on **a** particle size distribution and **b** apparent viscosity and zeta potential of EPS. Conditions: draw solution = 4 M NaCl, current density = 7.84

mA·cm^{-2} and UV fluence rate = 0.44 mW·cm^{-2}. (Please see Fig. S3 in the revised supplementary information).

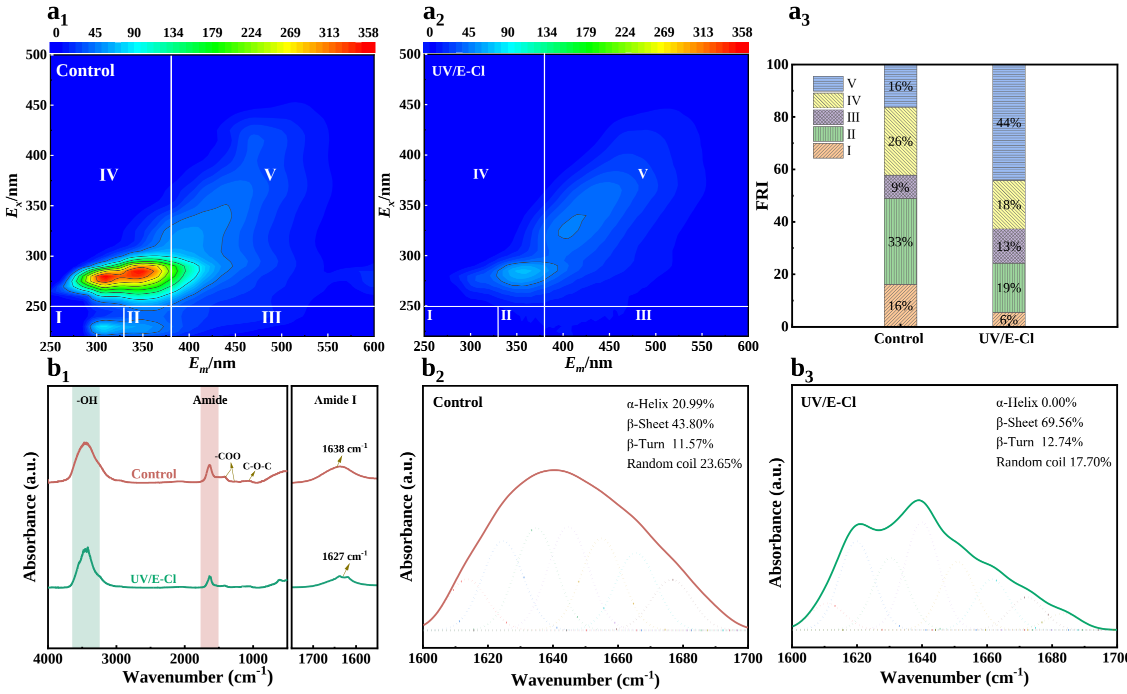

Fig. 6 **a** EEM spectra (a_1 refers to the control, a_2 refers to UV/E-Cl and a_3 refers to FRI distribution); and **b** FTIR analysis (b_1 refers to FTIR spectra in the control and UV/E-Cl, b_2 refers to amide I fitting curves in the control and b_3 refers to amide I fitting curves in UV/E-Cl) of EPS. Conditions: draw solution = 4 M NaCl, current density = 7.84 mA·cm^{-2} and UV fluence rate = 0.44 mW·cm^{-2}. (Please see Fig. S4 in the revised supplementary information).

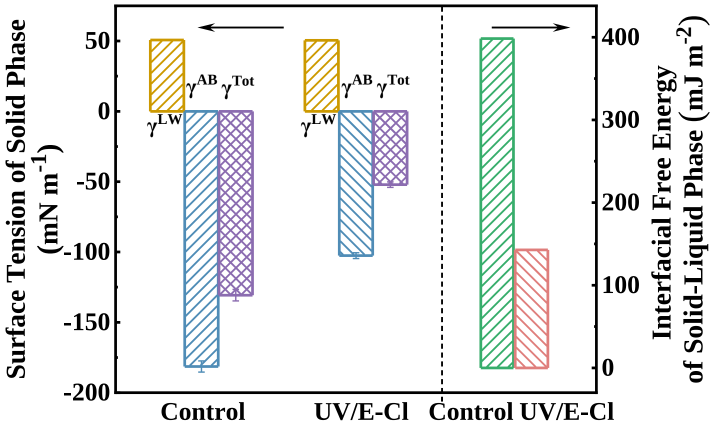

Fig. 7 EPS surface tension and interfacial free energy with water-phase. Conditions: draw solution = 4 M NaCl, current density = 7.84 mA·cm^{-2} and UV fluence rate = 0.44 mW·cm^{-2}. (Please see Fig. S5 in the revised supplementary information).

Additionally, the results of sludge dewatering experiments using 1 M NaCl as draw solution was retained and integrated into the revised manuscript to adequately validate the feasibility of this system to mitigate gel-like membrane fouling and improve sludge dewatering performance over a wide range of NaCl concentrations. (Fig. 8) (Please see lines 342-347 in the revised manuscript and Fig. S6 in the revised supplementary information). Notably, the higher draw solution concentration has a higher draw capacity, as evidenced by the 62% less time to recover 50% of the water content for the system using 4 M NaCl as the draw solution compared to the system using 1 M NaCl (Fig. 9). Overall, in-situ UV/E-Cl significantly improves the performance of FO process over a wide range of NaCl concentrations, demonstrating its high practical applicability in actual sludge dewatering. (Please see lines 347-353 in the revised manuscript and Fig. S7 in the revised supplementary information).

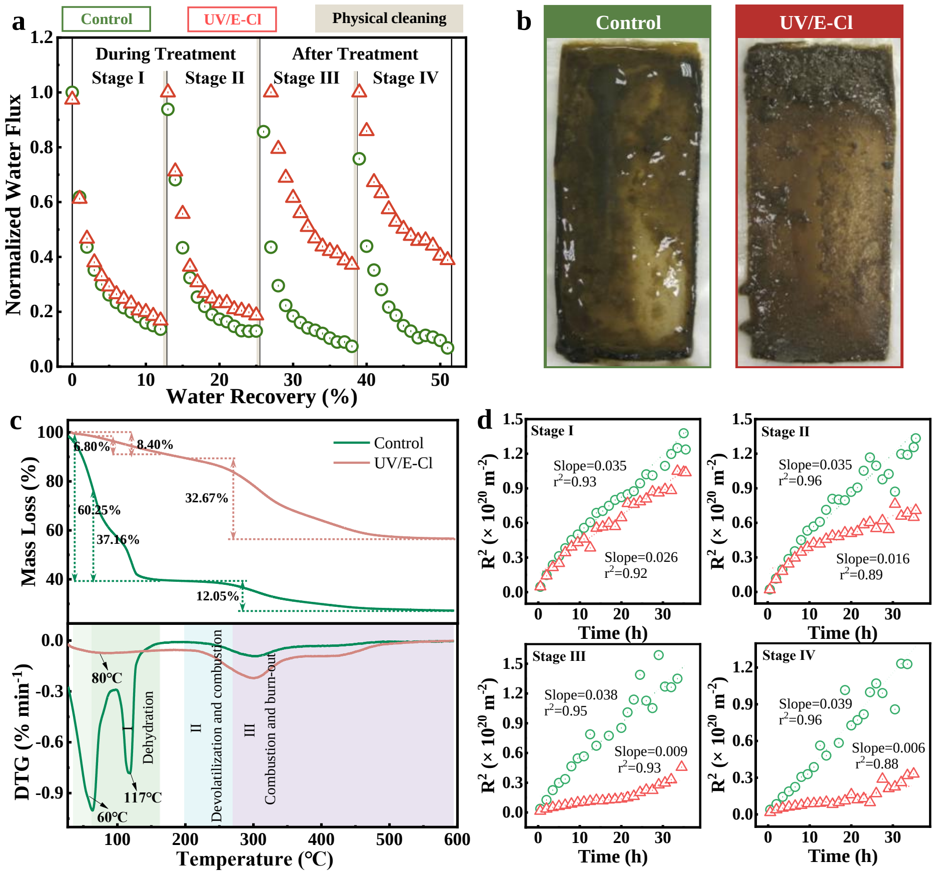

Fig. 8 Dewatering performance of UV/E-Cl assisted FO process for actual WAS: **a** normalized water flux; **b** direct observation of foulant layers; **c** thermal stabilities of

foulant layers; and **d** filtration resistance. Conditions: draw solution = 1 M NaCl, current density = 7.84 mA·cm^{-2} and UV fluence rate = 0.44 mW·cm^{-2}.

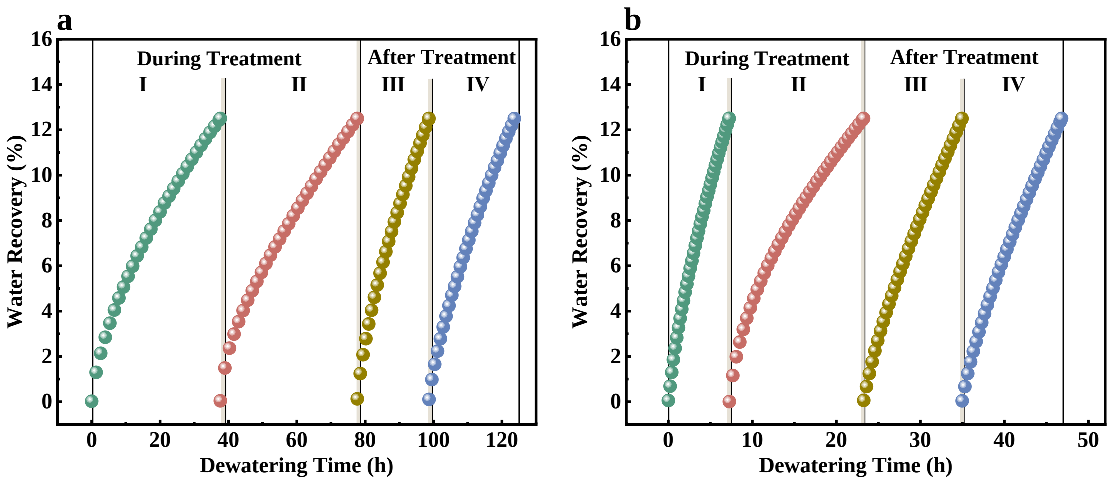

Fig. 9 Dewatering time required to recover 50% of the water recovery by the UV/E-Cl process using **a** 4 M NaCl and **b** 1 M NaCl as draw solution.

References

1. Shen, Z. et al. Exhaustive denitrification via chlorine oxide radical reactions for urea based on a novel photoelectrochemical cell. *Water Res.* **170**, 115357 (2020).
2. Huang, X. et al. Autocatalytic Decomplexation of Cu(II)–EDTA and Simultaneous Removal of Aqueous Cu(II) by UV/Chlorine. *Environ. Sci. Technol.* **53**, 2036-2044 (2019).
3. Fang, J., Fu, Y. & Shang, C. The Roles of Reactive Species in Micropollutant Degradation in the UV/Free Chlorine System. *Environ. Sci. Technol.* **48**, 1859-1868 (2014).
4. Guo, K. et al. Radical Chemistry and Structural Relationships of PPCP Degradation by UV/Chlorine Treatment in Simulated Drinking Water. *Environ. Sci. Technol.* **51**, 10431-10439 (2017).

Responses to Comments and Suggestions of Reviewers

Re manuscript: NCOMMS-23-55103B.

Title: Dynamic reverse Cl^- driven integration of sludge conditioning and dewatering: Emphasis on water occurrence states.

ANSWER: The authors are deeply grateful to the editor and reviewers for their thoughtful consideration of our revised manuscript. We highly value the insightful and constructive feedback provided by the reviewers, and we wholeheartedly agree that the suggested revisions will significantly enhance the robustness and completeness of our study. We have meticulously addressed each of the reviewer comments and incorporated the proposed changes into the revised manuscript and supplementary information. The modifications made in the revised manuscript and supplementary information are highlighted in red. Below, we provide detailed point-by-point responses to all the reviewer comments, outlining the specific changes we have made to address their concerns.

Responses to Reviewer #1 's comments:

General comments:

The authors have well addressed most of my comments and improved the manuscript. To enhance the robustness and completeness of the article, further optimization of experimental parameters, improvement of data clarity, and additional theoretical support and future outlooks are necessary. The detailed reviews are as follows:

ANSWER: We are particularly grateful for your high approval of our revised manuscript and for the time and effort you have dedicated to providing us with constructive comments. We understand your desire to enhance the robustness and completeness of the article. We will address each of the issues and suggestions carefully to further improve the quality of our manuscript.

Specific comments:

1. The article provides a comprehensive background on sludge dewatering, including a thorough analysis of the potential and existing challenges associated with FO technology. It is recommended to include a detailed comparison of current

mainstream dewatering technologies to highlight the innovations of this study. Specifically, an analysis of the advantages and disadvantages of other membrane fouling mitigation techniques would better illustrate the unique benefits of the dynamic reverse chloride-driven UV/E-Cl process.

ANSWER: We value your insightful and constructive comments. To date, reports on FO membrane fouling controlling strategies in WAS dewatering processes remain limited. Several studies have explored strategies such as ultrasound[1,2], membrane modification[3,4] and sludge conditioning[5] for mitigating membrane fouling in WAS treatment using FO technology. Nevertheless, there is no doubt that ultrasound complicates sludge treatment processes and increase operational energy consumption. Membrane modification is a complex and expensive process, and even cause the release of hazardous waste. Chemical additives increase treatment costs. Moreover, these techniques may not be as efficient as desired due to their non-specific targeting of proteins and polysaccharides, limiting their effectiveness in mitigating gel-like membrane fouling.

In contrast to these approaches, the dynamic reverse chloride-driven UV/E-Cl process proposed in this study offers a more targeted, cost-efficient, and environmentally friendly solution. Chlorine radical (Cl•) is noted for high reduction potential (~2.47 V) and could react rapidly with electron-rich molecules at nearly diffusion-controlled rates (~10^{10} M^{-1} s^{-1})[6-8]. The process utilizes chlorine radical (Cl•) to selectively attack electron-rich molecules such as glycosidic (C-O-C) bonds in polysaccharides and peptide (CO-NH) bonds in proteins, effectively mitigating gel-like membrane fouling during sludge dewatering. Furthermore, the process harnesses the inevitable and unwanted reverse Cl^{-} phenomenon during FO processes, converting it into a driving force for in-situ Cl• generation. This eliminates the need for additional chemical dosing, is less material-restricted, more flexible and continuous in operation.

The above information has been succinctly integrated into the revised manuscript to better illustrate the unique benefits of the dynamic reverse chloride-driven UV/E-Cl process. (Please see lines 42-46 and 51-58 in the revised manuscript).

2. The analysis of experimental results is thorough, particularly with the innovative application of thermodynamic theories to explain membrane fouling mechanisms. However, it is advisable to include an uncertainty analysis of the experimental results, addressing factors that might affect reproducibility, such as variations in initial sludge composition and membrane aging.

ANSWER: Thank you for your approval of our analysis of the experimental results. We are grateful for your insightful suggestion regarding uncertainty analysis, and we strongly agree that this is important to improve the reproducibility of our experimental results. Specifically, we recognize the following factors could influence the outcomes:

1. Sludge composition. Our experimental results demonstrated that the effectiveness of the UV/E-Cl process in controlling FO membrane gel fouling during sludge dewatering is due to the transformation of water occurrence states in the system, especially by attacking proteins and polysaccharides to release bound water. Accordingly, variations in the initial sludge composition, particularly in macromolecular organic loads, proteins and polysaccharides proportions, and bound water content, may affect sludge fouling trends and the overall performance of UV/E-Cl process for sludge dewatering. The uncertainty analysis regarding sludge composition have been added to the revised manuscript. (Please see lines 347-351 in the revised manuscript).

2. Membrane aging. Our experiments verified the integrity of the membrane performance after 4-cycle UV/E-Cl processes, possibly due to the high organic loading of the sludge acting as a free radical shield for the membrane. Nevertheless, the possibility of membrane aging after long-term operation may not be ignored. This may lead to an increase in reverse salt diffusion and a loss of membrane permeation performance. To address this concern, we plan to investigate the feasibility of applying antioxidant-modified membranes or inorganic membranes in future studies, aiming to mitigate membrane aging and enhance the system long-term stability. The uncertainty analysis regarding membrane aging have been added to the revised manuscript. (Please see lines 381-387 in the revised manuscript).

3. Sludge external conditions. Other parameters, e.g., pH and temperature may also impact experimental results. For example, variations in pH can affect the efficiency of Cl^{-} electro-oxidation and the type of activated chlorine. Similarly, solution temperature affects the electrochemical reaction rate and even the solubility of chlorine gas. In future work, it is important to investigate the impacts of different water quality parameters, such as pH and temperatures, on the formation of chlorine radicals and the overall dewatering efficiency. (Please see lines 396-398 in the revised manuscript).

3. NaCl is used as the draw solution in the experiments, but it is important to note that high-concentration sodium chloride solutions possess inherent dehydration capabilities. Please clarify how to differentiate the specific role of NaCl in the experiments—whether the RCS acts as the primary driver of dehydration or if sodium chloride itself contributes to the dewatering of the sludge. Additionally, further exploration of the impact of varying NaCl concentrations on system performance would help assess the applicability and limitations of the process.

ANSWER: Thank you for your insightful comment. We recognize that differentiating whether the RCS is the primary driver of dehydration or if NaCl itself contributes to the sludge dewatering is crucial. To address this, we have added the following estimates (based on Eqs. 1-2) and presented the results in Table 1. Our findings show that RCS acts as the primary driver in the improved dewatering performance of 4 M NaCl compared to 1 M NaCl, accounting for 89.31% of the enhancement. Fig. 1 illustrates that 4 M NaCl as the draw solution induces a relatively higher reverse salt flux, contributing to the production of more RCS. While high-concentration NaCl solutions theoretically possess inherent dehydration capabilities, our results suggest that their contribution to dewatering performance may not be absolutely dominant. As reported in the literatures[9-11], higher draw-off fluxes instead lead to increased deposition of foulants on the membrane surface, accelerating membrane fouling. This phenomenon was also observed in our study, as evidenced by the filtration resistance analysis ($slope_{4M\text{-}stageIII} = 0.040 > slope_{1M\text{-}stageIII} = 0.038$, $slope_{4M\text{-}stageIV} = 0.048 > slope_{1M\text{-}stageIV}$

= 0.039) presented in Fig. 2. Overall, high-concentration NaCl possess relatively stronger inherent dehydration capabilities, on top of which more reverse Cl^{-} significantly drives the improvement in dewatering performance. The above information has been integrated into the revised manuscript (Please see lines 360-363 in the revised manuscript, Fig. 8 and Table 5 in the revised supplementary information).

While, high-concentration NaCl also pose significant resource requirements. For practical applications, utilizing readily available and more practical concentrations like 1M NaCl (comparable to seawater) offers a cost-effective and sustainable approach. In scenarios where high-salinity sources are readily accessible, such as reverse osmosis concentrates, these can be strategically employed to maximize dewatering efficiency while minimizing resource consumption. The specific recommendations for choosing appropriate NaCl concentrations based on factors like resource availability and dewatering performance have been incorporated in the revised manuscript with a view to providing more comprehensive guidance for practical application, optimizing both cost-effectiveness and efficiency. (Please see lines 363-369 in the revised manuscript).

$$\text{Inherent contribution }(\%) = \frac{J_{\text{e (4M-control)}} - J_{\text{e (1M-control)}}}{J_{\text{e (4M-UVECl)}} - J_{\text{e (1M-UVECl)}}} \quad (1)$$

$$\text{RCS contribution }(\%) = \frac{\left(J_{\text{e (4M-UVECl)}} - J_{\text{e (4M-control)}}\right) - \left(J_{\text{e (1M-UVECl)}} - J_{\text{e (1M-control)}}\right)}{J_{\text{e (4M-UVECl)}} - J_{\text{e (1M-UVECl)}}} \quad (2)$$

where $J_{4M\text{-control}}$ is the water flux at the end of recovering 50% water content with no treatment and 4 M NaCl as draw solution. $J_{4M\text{-UVECl}}$ is the water flux at the end of recovering 50% water content with UV/E-Cl treatment and 4 M NaCl as draw solution. $J_{1M\text{-control}}$ is the water flux at the end of recovering 50% water content with no treatment and 1 M NaCl as draw solution. $J_{1M\text{-UVECl}}$ is the water flux at the end of recovering 50% water content with UV/E-Cl treatment and 1 M NaCl as draw solution.

Table 1. Evaluation of the specific role of NaCl in the experiments.

$J_{4M\text{-}control}$ (LMH)	$J_{4M\text{-}UVECl}$ (LMH)	$J_{1M\text{-}control}$ (LMH)	$J_{1M\text{-}UVECl}$ (LMH)	Inherent contribution (%)	RCS contribution (%)
0.65	4.74	0.37	2.12	10.69	89.31

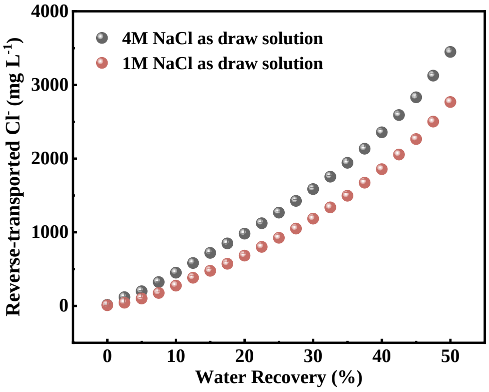

Fig. 1 Reverse-transported Cl⁻ concentration during 4 M NaCl and 1 M NaCl as draw solution.

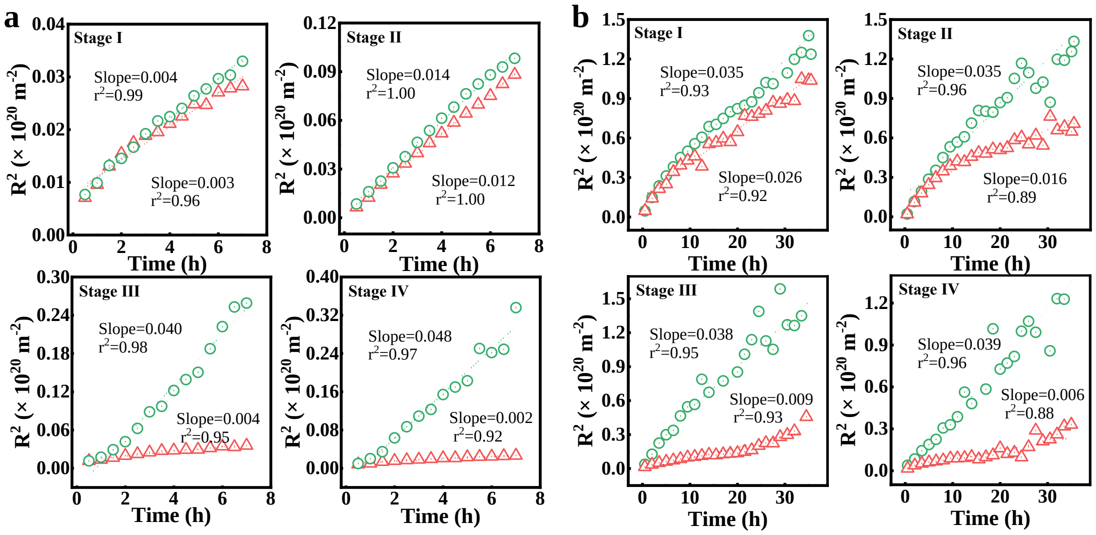

Fig. 2 Filtration resistance analysis during 4 M NaCl (a) and 1 M NaCl (b) as draw solution.

4. Please elaborate on the advantages of using AOP for sludge dewatering. Most advanced oxidation methods disrupt the physicochemical structure of EPS, typically targeting peptide bonds in proteins and glycosidic bonds in polysaccharides. The experiments demonstrate that these technologies generate free radicals that attack EPS, but they do not fully illustrate their comparative superiority.

ANSWER: Thank you for your thoughtful feedback. The presence of organic phase,

particularly EPS in sludge leads to a portion of water being embedded within bio-polymeric networks through chemical binding (hydrogen bonding), physical adsorption and mechanical capture. Notably, proteins and polysaccharides in EPS exhibit a strong ability to mechanically trap interstitial bound water through specific cross-linked network structures and adsorb interfacial bound water through abundant hydrophilic sites. Bound water is difficult to remove from the solid phase using mechanical dewatering methods. It significantly limits sludge dewatering and is a key contributor to induce gel-like membrane fouling during membrane-associated sludge dewatering processes. Substantial lysis and release of EPS contribute to the release of bound water from sludge flocs. Accordingly, AOP approaches aimed at enhancing EPS depolymerization and eliminating its water-holding capacity have been widely reported as effective strategies for improving sludge dewaterability. The above information has been elaborated in the revised manuscript to highlight the advantages of employing AOP for sludge dewatering (Please see lines 37-42 and 47-49 in the revised manuscript).

Nevertheless, HO• and SO_4^{-}• are non-selective and readily quenched by dissolved organic matter and inorganic salts (e.g., CO_3^{2-}, Cl^{-}), potentially limiting their targeted attack on EPS and subsequent improvement in sludge dewaterability. Reactive chlorine species (e.g., Cl•, ClO• and Cl_2•$^{-}$) are note for their selective attack on electron-rich molecules at near diffusion-controlled rates (~10^{10} M^{-1} s^{-1}). It is anticipated that chlorine-based AOP could preferentially react with electron-rich sites, such as glycosidic bonds (C-O-C) in polysaccharides and peptide bonds (CO-NH) in proteins, thereby disrupting the structural integrity of EPS and diminishing its hydration capacity. Therefore, we propose to investigate the applicability of chlorine-based AOP in improving sludge dewaterability. As expected, chlorine-based AOP significantly disrupts the cross-linked network structure of organics, as evidenced by smaller particle size, structural fragmentation (SEM images), and a reduction in glycosidic and amide bonds (FTIR analysis). This is apparently effective in releasing mechanically trapped interstitial bound water. Additionally, chlorine-based AOP significantly reduces hydrophilic sites and increases the exposure of hydrophobic sites,

as confirmed by reduced surface polarity, reduced O-H, -COO and N-H groups, and disruption of protein secondary structure. This effectively reduces adsorbed interfacial bound water. Ultimately, thermogravimetric analysis reveals a reduction of over 80% in the total amount of bound water in both simulated and actual sludge. The above synopsis from the article could clearly illustrate the comparative superiority of chlorine-based AOP in controlling gel fouling and improving sludge dewaterability.

5. The conclusion effectively summarizes the main research findings but provides a rather brief outlook on future research. It is suggested that the authors elaborate on the practical application potential of the process in real-world sludge treatment and propose specific research directions, such as further optimization of UV/E-Cl process parameters, exploring synergistic effects with other oxidants, or assessing the technology's applicability to different types of sludge.

ANSWER: We are sincerely grateful for your positive feedback on the effectiveness of our conclusion in summarizing the main research findings. We have recognized the importance of providing a more comprehensive outlook on future research directions. This study validates the excellent performance of the proposed dynamic reverse Cl^--driven in-situ UV/E-Cl assisted FO process in mitigating membrane gel fouling and enhancing WAS dewatering at the laboratory scale. Nevertheless, to further enhance the process performance and facilitate its translation to larger-scale practical applications, it is important in future work to focus on optimizing reactor conditions and parameters to better mimic realistic scenarios. This includes investigating the effects of electrode material and distance, UV intensity, and total effective treatment time, or assessing the impact of sludge composition, pH, and temperature. Exploring the potential synergistic effects of in-situ UV/E-Cl with other oxidants, such as hydrogen peroxide cathodogenesis and free chlorine anodogenesis for the co-generation of free radicals, would also be valuable. Notably, although this work was carried out with WAS system, the novel finding of "water occurrence states-dominated membrane gel fouling" holds universal significance for gel fouling mitigation and membrane processes operation, regardless of wastewater type,

membrane model type, or reactor scale. Consequently, future applications of the UV/E-Cl assisted FO process could be expanded to various types of sludges, such as agricultural sludge and aquaculture sludge with high organic loads and substantial bound water content. Furthermore, the process could be explored for its potential in other systems, including algae solutions dewatering, waste leachate treatment, and agricultural wastewater treatment. (Please see lines 393-409 in the revised manuscript).

Responses to Reviewer #2 's comments:

Specific Comments：

1. L68-69. The author characterized the sludge by introducing organic matter, including polysaccharides and proteins. This suggests that the author's assumption is flawed, indicating a focus on investigating organic fouling in the FO system rather than comprehensively addressing the broader aspects of sludge. Additionally, references (55 and 64) fail to establish a connection with the dewatering experiment (L343-345).

2. L345-346. Why was a higher concentration of NaCl (4 M) used as the draw solution in this experiment, considering that the NaCl concentration in natural seawater typically ranges from 0.5 M to 1.0 M? Additionally, why was a 1,000 mg/L SA-BSA mixture used for the model sludge concentration in these experiments?

3. L 125. The reverse salt (Cl) flux and free chlorine concentration are the most important factors in this experiment. Therefore, the author should provide reverse salt flux and free chlorine data for each experimental case (UV/E-Cl, E-Cl, control, UV).

4. L136. I confused the conditions with treatment and without treatment. How could higher performance be achieved without treatment in Figure 2a?

5. L159. The floc size decreased after the UV/E-Cl treatment. Generally, a smaller floc size could accelerate the formation of a more dense fouling layer compared to larger flocs. However, the experimental results contradict this observation. Why?

6. L254. I cannot find information about Figures 7a and 7b in the manuscript. Additionally, were the DFT simulations conducted by the authors, or were the results cited from other works? The author should provide a more detailed explanation about the DFT simulations.

7. L281-286. The explanation is quite abstract, and a more direct explanation should be provided based on the experimental results.

8. L292-335. The proposed mechanism illustrates changes in the formation of the organic fouling layer by the UV/E-Cl system, but it does not focus on the aspects of dewatering waste activated sludge. As I asked in Q1, the experimental concept is closer to the control of organic fouling in FO rather than the sludge dewatering system.

ANSWER: We appreciate the reviewer's repeated comments, but we believe all these points have been raised and addressed in the previous round of review.

References

1. Nguyen, N. C., Nguyen, H. T., Chen, S.-S., Nguyen, N. T. & Li, C.-W. Application of forward osmosis (FO) under ultrasonication on sludge thickening of waste activated sludge. *Water Sci. Technol.* **72**, 1301-1307 (2015).
2. Lee, S., Shon, H. K. & Hong, S. Dewatering of activated sludge by forward osmosis (FO) with ultrasound for fouling control. *Desalination* **421**, 79-88 (2017).
3. Yi, X. et al. A novel fouling control strategy for forward osmosis membrane during sludge thickening via self-forming protective layer. *Sep. Purif. Technol.* **319**, 124069 (2023).
4. Ng, D. Y. F., Wu, B., Chen, Y., Dong, Z. & Wang, R. A novel thin film composite hollow fiber osmotic membrane with one-step prepared dual-layer substrate for sludge thickening. *J. Membr. Sci.* **575**, 98-108 (2019).
5. Sun, F., Lu, D., Ho, J. S., Chong, T. H. & Zhou, Y. Mitigation of membrane fouling in a seawater-driven forward osmosis system for waste activated sludge thickening. *J. Clean. Prod.* **241**, 118373 (2019).
6. Fang, J., Fu, Y. & Shang, C. The Roles of Reactive Species in Micropollutant Degradation in the UV/Free Chlorine System. *Environ. Sci. Technol.* **48**, 1859-1868 (2014).
7. Guo, K. et al. Radical Chemistry and Structural Relationships of PPCP Degradation by UV/Chlorine Treatment in Simulated Drinking Water. *Environ. Sci. Technol.* **51**, 10431-10439 (2017).
8. Wu, Z. et al. Roles of reactive chlorine species in trimethoprim degradation in the UV/chlorine process: Kinetics and transformation pathways. *Water Res.* **104**, 272-282 (2016).
9. Zou, S., Gu, Y., Xiao, D. & Tang, C. Y. The role of physical and chemical parameters on forward osmosis membrane fouling during algae separation. *J. Membr. Sci.* **366**, 356-362 (2011).
10. Wang, Y., Wicaksana, F., Tang, C. Y. & Fane, A. G. Direct Microscopic Observation of Forward Osmosis Membrane Fouling. *Environ. Sci. Technol.* **44**, 7102-7109 (2010).

11. Tang, C. Y., She, Q., Lay, W. C. L., Wang, R. & Fane, A. G. Coupled effects of internal concentration polarization and fouling on flux behavior of forward osmosis membranes during humic acid filtration. *J. Membr. Sci.* **354**, 123-133 (2010).

Responses to Comments and Suggestions of Reviewers

Re manuscript: NCOMMS-23-55103C-Z.

Title: Dynamic reverse Cl^{-} driven integration of sludge conditioning and dewatering: Emphasis on water occurrence states.

ANSWER: The authors would like to thank the editor and reviewer#1 for their time and effort, and for their acknowledgment of our manuscript. We highly value the critical and constructive comments provided by reviewer#1, and we agree that the suggested revisions will enhance the robustness and logical flow of our manuscript. We have thoroughly addressed each of the reviewer#1 comments and incorporated the suggested changes into the revised manuscript and supplementary information. All modifications are highlighted in red. The detailed point-by-point responses are provided below.

Responses to Reviewer #1 's comments:

General comments:

The authors have responded most comments. However, I find some of important concerns was not accrodingly revised in the manuscript. The major issues are:

ANSWER: We appreciate for your high approval of our revised manuscript and for identifying the remaining concerns in our revised manuscript. We have recognized that the previous revision was not sufficient to address your concerns. We are fully committed to revising the manuscript again to address each of these issues and to further strengthen the quality of our work.

Specific comments:

1. The formation of chlorinated byproducts should be mentioned in the manuscript, as people always concern the toxic chlorinated byproducts formed during E/UV-Cl.

ANSWER: Thank you for your valuable comment. We fully understand your concern about the formation of the toxic chlorinated byproducts during E/UV-Cl. Based on your reminder, we have now moved the detailed data on the formation of chlorinated byproducts from the supplementary information to the manuscript to enhance the readability and clarity of the manuscript. (Please see Fig. 2b in the revised manuscript). Additionally, we have refined and supplemented the brief overview of

chlorinated byproducts formation, including both carbonaceous DBPs (primarily THMs and HAAs) and nitrogenous DBPs (primarily HANs), as follows: “The chlorinated disinfection byproducts (DBPs) formation during WAS dewatering with UV/E-Cl was investigated (detailed methods in Supplementary Text 2.3). The negligible DBPs in draw solution underscored the high rejection efficiency of FO membrane (Fig. 2b draw solution)[1-3]. Dewatered sludge contained 55.1 $\mu g \cdot L^{-1}$ regulated trihalomethanes (THMs), 528.2 $\mu g \cdot L^{-1}$ regulated haloacetic acids (HAAs), and 40.7 $\mu g \cdot L^{-1}$ haloacetonitriles (HANs) (Fig. 2b feed solution). THMs and HANs were below WHO drinking water guidelines of 560 $\mu g \cdot L^{-1}$ and 90 $\mu g \cdot L^{-1}$, respectively[4]. Despite HAAs surpassed the 270 $\mu g \cdot L^{-1}$ drinking water guideline[5], subsequent anaerobic digestion (removing 68.68% DBPs) is deemed sufficient to mitigate environmental risks[6]”. (Please see lines 145-155 in the revised manuscript).

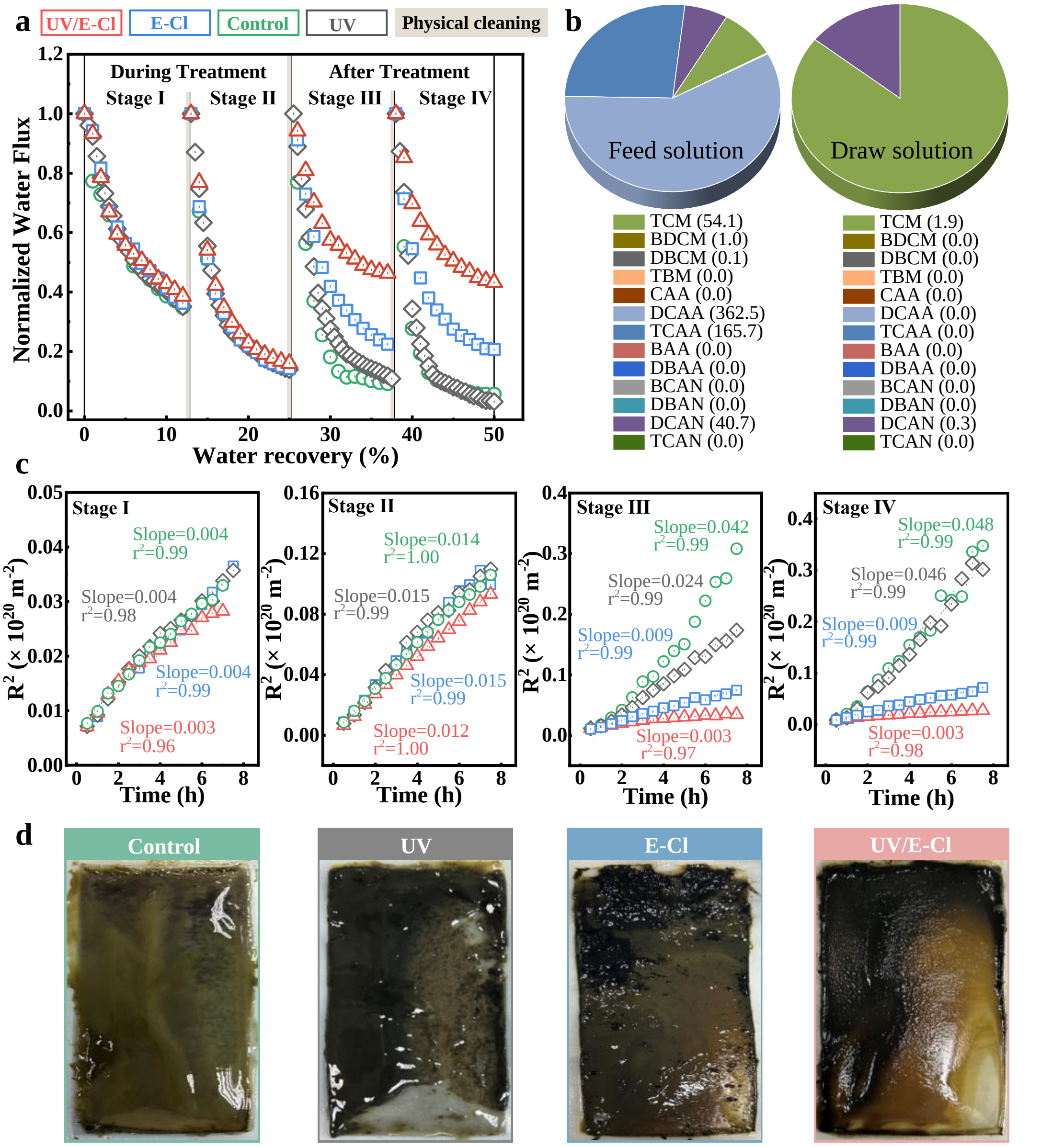

"Fig. 2. WAS dewatering performance assessment. a Normalized water flux with water recovery. Note that physical membrane cleaning was performed after each stage due to severe water flux decline in the control and UV processes. **b** DBPs determination in the feed and draw solutions. HANs: bromochloroacetonitrile (BCAN), dibromoacetonitrile (DBAN), dichloracetonitrile (DCAN), and trichloroacetonitrile (TCAN)). THMs: chloroform (TCM), bromodichloromethane (BDCM), chlorodibromomethane (DBCM), and bromoform (TBM). HAAs: monochloroacetic acid (CAA), dichloroacetic acid (DCAA), trichloroacetic acid (TCAA), monobromoacetic acid (BAA), and dibromoacetic acid (DBAA). **c** Cake filtration model fitting for water flux. **d** Direct observation of foulant layers.

Experimental conditions: draw solution = 4 M NaCl, current density = 7.84 mA·cm^{-2}, UV power = 10 W, UV fluence rate = 0.44 mW·cm^{-2}, and exposure mode = 5'-ON/25'-OFF."

2. Even though the authors hold the opinion that SA-BSA can stand for actual sludge. The property of SA-BSA is totally different from autual sludge. For example, the former is dissoved, the latter is biological particles. The results of actual sludge should be provided in the main text.

ANSWER: Thanks for your reiteration of this concern, which has prompted us to recognize the necessity to include actual sludge results in the main manuscript text. We understand that the precedence of actual sludge results is crucial for the integrity and logic of the manuscript. Accordingly, the manuscript has been modified to incorporate the actual sludge dewatering performance and characterization results into the main text for discussion. (please see the revised manuscript).

After a detailed discussion of the actual sludge dewatering performance, we identified that, UV/E-Cl process effectively disrupted the physical structure and chemical composition of extracellular polymeric substances (EPS) by targeting its major components—proteins and polysaccharides, primarily mitigated membrane fouling (please see Fig. 2c-d and Fig. 3 in the revised manuscript). This result aligns with the general consensus in the literature[7-11] that proteins and polysaccharides in sludge are the primary components responsible for membrane fouling. To unravel the detailed membrane fouling influential mechanisms, a thorough understanding of these protein and polysaccharide fractions should be further investigated. However, the inherent structural complexity and variability of these components[12] have severely limited our ability to gain insights into the membrane fouling behaviors at a more microscopic level, including their molecular-level interactions, their synergistic membrane fouling behavior, their functional role in hydration state differences, and thermodynamic simulations of whole-membrane fouling processes. Given the current limitations in accurately resolving the precise structures of proteins and polysaccharides, the use of model pollutants has emerged as an effective strategy for

revealing general mechanisms[13-15]. Bovine serum albumin (BSA) and sodium alginate (SA) are frequently used as models for proteins and polysaccharides in EPS, respectively, due to their comparable structural properties and fouling behaviors[16-18].

Accordingly, we conducted the dewatering experiments on a combined solution of SA and BSA (SA-BSA mixtures). The similar dewatering performance and order of magnitude of filtration resistance observed with both the SA-BSA mixture and actual sludge under UV/E-Cl (please see supplementary Fig. 3 in the revised supplementary information) suggests the dominant role of protein and polysaccharide in sludge dewatering behaviors, and demonstrates the relevance of SA-BSA model in adequately simulating the complex behavior of EPS under realistic dewatering scenarios. By utilizing the typical SA-BSA model system, we were able to gain a deeper understanding of the underlying mechanisms through quantum mechanical calculations (please see Fig. 4h in the revised manuscript) to provide molecular-level clues for protein-polysaccharide interactions, and filtration thermodynamic simulation (please see Table 1 and Fig. 7. in the revised manuscript) to provide the quantitative evidence for dominance of water occurrence states. These insights provide a foundation for understanding the more complex real sludge system and are critical for developing more effective and targeted solutions for sludge dewatering.

1. Xu, J., Tran, T. N., Lin, H. & Dai, N. Modeling the transport of neutral disinfection byproducts in forward osmosis: Roles of reverse salt flux. *Water Res.* **185**, 116255 (2020).
2. Coday, B. D., Yaffe, B. G. M., Xu, P. & Cath, T. Y. Rejection of trace organic compounds by forward osmosis membranes: A literature review. *Environ. Sci. Technol.* **48**, 3612-3624 (2014).
3. Lee, H., Im, S.-J., Park, J. H. & Jang, A. Removal and transport behavior of trace organic compounds and degradation byproducts in forward osmosis process: Effects of operation conditions and membrane properties. *Chem. Eng. J.* **375**, 122030 (2019).
4. WHO. Guidelines for drinking-water quality, 3rd edition: Volume 1-Recommendations incorporating the first and second addenda, Geneva (2008).
5. Wang, X., Mao, Y., Tang, S., Yang, H. & Xie, Y. F. Disinfection byproducts in drinking water and regulatory compliance: A critical review. *Front. Environ. Sci. Eng.* **9**, 3-15 (2015).
6. Guo, Y. et al. Variations of heavy metals, nutrients, POPs and particle size distribution during "sludge anaerobic digestion-solar drying-land utilization process": Case study in China. *Sci. Total. Environ.* **801**, 149609 (2021).
7. Wu, M. et al. High propensity of membrane fouling and the underlying mechanisms in a membrane bioreactor during occurrence of sludge bulking. *Water Res.* **229**, 119456 (2023).
8. Teng, J., Zhang, H., Tang, C. & Lin, H. Novel molecular level insights into forward osmosis membrane fouling affected by reverse diffusion of draw solutions based on thermodynamic mechanisms. *J. Membr. Sci.* **620**, 118815 (2021).
9. Duc Viet, N., Im, S.-J. & Jang, A. Characterization and control of membrane fouling during dewatering of activated sludge using a thin film composite forward osmosis membrane. *J. Hazard. Mater.* **396**, 122736 (2020).
10. Yi, X. et al. Novel insights on fouling mechanism of forward osmosis membrane during deep thickening waste activated sludge. *J. Membr. Sci.* **660**, 120894 (2022).
11. Yi, X., Zhong, H., Xie, M. & Wang, X. A novel forward osmosis reactor assisted

with microfiltration for deep thickening waste activated sludge: performance and implication. *Water Res.* **195**, 116998 (2021).

12. Yu, H.-Q. Molecular Insights into Extracellular Polymeric Substances in Activated Sludge. *Environ. Sci. Technol.* **54**, 7742-7750 (2020).
13. Wheeler, K. E. et al. Environmental dimensions of the protein corona. *Nature Nanotechnology* **16**, 617-629 (2021).
14. Huang, X. et al. Vibrio cholerae biofilms use modular adhesins with glycan-targeting and nonspecific surface binding domains for colonization. *Nat. Commun.* **14**, 2104 (2023).
15. Qin, D., Liu, Z., Liu, Z., Bai, H. & Sun, D. D. Superior Antifouling Capability of Hydrogel Forward Osmosis Membrane for Treating Wastewaters with High Concentration of Organic Foulants. *Environ. Sci. Technol.* **52**, 1421-1428 (2018).
16. Ang, W. S. & Elimelech, M. Protein (BSA) fouling of reverse osmosis membranes: Implications for wastewater reclamation. *J. Membr. Sci.* **296**, 83-92 (2007).
17. Li, Q. & Elimelech, M. Synergistic effects in combined fouling of a loose nanofiltration membrane by colloidal materials and natural organic matter. *J. Membr. Sci.* **278**, 72-82 (2006).
18. Ang, W. S., Lee, S. & Elimelech, M. Chemical and physical aspects of cleaning of organic-fouled reverse osmosis membranes. *J. Membr. Sci.* **272**, 198-210 (2006).
